# Metavirome of 31 tick species provides a compendium of 1,801 RNA virus genomes

**DOI:** 10.1038/s41564-022-01275-w

**Published:** 2023-01-05

**Authors:** Xue-Bing Ni, Xiao-Ming Cui, Jin-Yue Liu, Run-Ze Ye, Yu-Qian Wu, Jia-Fu Jiang, Yi Sun, Qian Wang, Marcus Ho-Hin Shum, Qiao-Cheng Chang, Lin Zhao, Xiao-Hu Han, Ke Ma, Shi-Jing Shen, Ming-Zhu Zhang, Wen-Bin Guo, Jin-Guo Zhu, Lin Zhan, Liang-Jing Li, Shu-Jun Ding, Dai-Yun Zhu, Jie Zhang, Luo-Yuan Xia, Xiang-Yong Oong, Xiang-Dong Ruan, Hong-Ze Shao, Teng-Cheng Que, Guang-Yuan Liu, Chun-Hong Du, En-Jiong Huang, Xin Wang, Li-Feng Du, Chong-Cai Wang, Wen-Qiang Shi, Yu-Sheng Pan, Yu-Hao Zhou, Jiang-Li Qu, Jiang Ma, Cai-Wei Gong, Qi-Qing Chen, Qian Qin, Tick Genome and Microbiome Consortium (TIGMIC), Tommy Tsan-Yuk Lam, Na Jia, Wu-Chun Cao

**Affiliations:** 1 State Key Laboratory of Pathogen and Biosecurity, Beijing Institute of Microbiology and Epidemiology, Beijing, P. R. China; 2 State Key Laboratory of Emerging Infectious Diseases and Centre of Influenza Research, School of Public Health, The University of Hong Kong, Hong Kong SAR, P. R. China; 3 Laboratory of Data Discovery for Health Limited, Hong Kong SAR, P. R. China; 4 Research Unit of Discovery and Tracing of Natural Focus Diseases, Chinese Academy of Medical Sciences, Beijing, P. R. China; 5 Institute of EcoHealth, School of Public Health, Shandong University, Jinan, P. R. China; 6 School of Public Health, Shantou University, Shantou, Guangdong Province P. R. China; 7 Shenyang Agriculture University, Shenyang, P. R. China; 8 ManZhouLi Customs District, Manzhouli, Inner Mongolia P. R. China; 9 Central Laboratory, Guizhou Provincial People’s Hospital, Guiyang, P. R. China; 10 Shandong Center for Disease Control and Prevention, Shandong Provincial Key Laboratory of Communicable Disease Control and Prevention, Jinan, P. R. China; 11 Academy of Forest Inventory and Planning, State Forestry and Grassland Administration, Beijing, P. R. China; 12 Animal Husbandry and Veterinary Science Research Institute of Jilin Province, Changchun, P. R. China; 13 Guangxi Zhuang Autonomous Region Terrestrial Wildlife Medical-aid and Monitoring Epidemic Diseases Research Center, Nanjing, P. R. China; 14 State Key Laboratory of Veterinary Etiological Biology, Key Laboratory of Veterinary Parasitology of Gansu Province, Lanzhou Veterinary Research Institute, Chinese Academy of Agricultural Science, Lanzhou, P. R. China; 15 Yunnan Institute for Endemic Diseases Control and Prevention, Dali, P. R. China; 16 Fuzhou International Travel Healthcare Center, Fuzhou, Fujian, P. R. China; 17 Qingjiangpu District Center for Disease Control and Prevention, Huai’an, P. R. China; 18 Hainan International Travel Healthcare Center, Haikou, P. R. China; 19 Development of Xinjiang Yili Gongliu County Animal Husbandry and Veterinary Medicine Center, Urumqi, P. R. China; 20 Animal Husbandry Development Center of Qiannan Buyei and Miao Autonomous Prefecture, Qian’nan, P. R. China; 21 Animal Husbandry Development Center of Huishui County, Guiyang, P. R. China; 22 Animal Husbandry and Aquatic Product Development Promotion Center of Libo County, Qian’nan, P. R. China; 23 Centre for Immunology and Infection Limited, Hong Kong SAR, P. R. China; 24 Guangdong-Hongkong Joint Laboratory of Emerging Infectious Diseases, Joint Institute of Virology (Shantou University/The University of Hong Kong), Shantou, P. R. China; 25 EKIH (Gewuzhikang) Pathogen Research Institute, Shenzhen, P. R. China; 26 The representative of Tick Genome and Microbiome Consortium (TIGMIC), Beijing, P. R. China

**Keywords:** Viral vectors, Metagenomics, Phylogenetics

## Abstract

The increasing prevalence and expanding distribution of tick-borne viruses globally have raised health concerns, but the full repertoire of the tick virome has not been assessed. We sequenced the meta-transcriptomes of 31 different tick species in the *Ixodidae* and *Argasidae* families from across mainland China, and identified 724 RNA viruses with distinctive virome compositions among genera. A total of 1,801 assembled and complete or nearly complete viral genomes revealed an extensive diversity of genome architectures of tick-associated viruses, highlighting ticks as a reservoir of RNA viruses. We examined the phylogenies of different virus families to investigate virome evolution and found that the most diverse tick-associated viruses are positive-strand RNA virus families that demonstrate more ancient divergence than other arboviruses. Tick-specific viruses are often associated with only a few tick species, whereas virus clades that can infect vertebrates are found in a wider range of tick species. We hypothesize that tick viruses can exhibit both ‘specialist’ and ‘generalist’ evolutionary trends. We hope that our virome dataset will enable much-needed research on vertebrate-pathogenic tick-associated viruses.

## Abstract (web summary)

The metavirome of 31 tick species sheds light on the diversity and distinct evolution of tick-associated RNA viruses, and lays the foundation for better understanding of tick–virus interaction.

## Main

Arthropods, such as ticks, mosquitos and fleas, are among the most abundant animals on Earth and can transmit a variety of viruses (arbovirus) to humans and animals^1^. The arboviral transmission cycle always involves the intricate interactions among viruses, arthropod vectors and animal hosts, inherently shaping the evolution and diversification of the viruses. Ticks (*Acari*: *Ixodida*) are obligate blood-feeding vectors and an ideal model for studying arbovirus transmission owing to their three life stages (larvae, nymphs and adults), blood-feeding of each life stage on various animal hosts, and adaptation to diverse ecological environments. Compared with other haematophagous arthropods, ticks maintain and transmit the widest array of human and animal pathogens^2^.

Tick-borne viruses are a global health concern due to the increasing prevalence and geographical coverage of ticks, and the potential for them to vector emerging and re-emerging viruses^3,4^. Although the viromes of several tick species have been reported^5–13^, the complement of the tick virome remains to be reported. Notably, arboviruses are transmitted horizontally by blood-feeding invertebrate vectors but can also be transmitted vertically from an infected female tick to its offspring. Both types of transmission have shaped the evolutionary trajectory of arboviruses^14–16^. The evolutionary and ecological characteristics of tick-associated viruses, either as multiple-host generalists or single-host specialists, remain unclear.

Here we carried out a large-scale meta-transcriptomic study of 31 tick species from mainland China to characterize the tick virome, identify factors shaping viral composition and investigate the evolutionary history of tick-associated viruses. Our analyses provide resources for research into arbovirus transmission from ticks to humans or animals.

## Results

### Analysing the tick virome

**Fig. 1:**
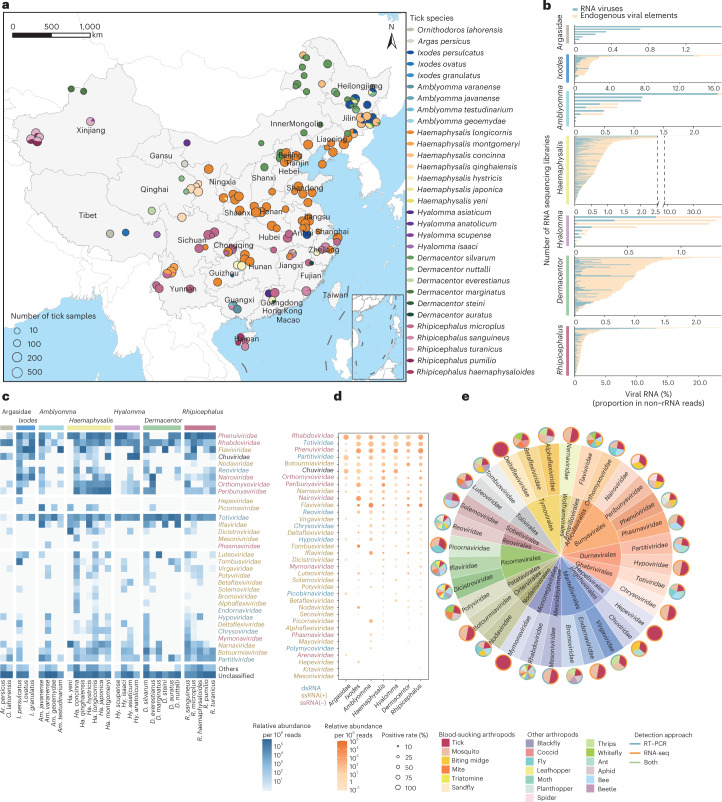
Overview of the tick virome. **a**, Geographic map of sample collections. The size of the circle represents the number of tick samples collected in the area. Each sampling site was geo-referenced to the Chinese map on the basis of its latitude and longitude. **b**, The proportion of viral RNA reads in non-ribosome reads among ticks in family *Argasidae* and six genera of the family *Ixodidae*. **c**, The relative abundance of the top 32 viral families (>1 per ten thousand viral reads in any tick species) detected in each tick species. **d**, The relative virus abundances (viral read counts per million non-rRNA reads in each library) and positive rates of the top 20 viral families among ticks in the family *Argasidae* and six genera of the family *Ixodidae*. **e**, Comparison between viral families detected in our ticks and those previously reported in other arthropods. The detection approaches for viral families in other arthropods include RT–PCR, RNA-seq or both.

We analysed 31 tick species from six genera (*Ixodes*, *Amblyomma*, *Haemaphysalis*, *Hyalomma*, *Dermacentor* and *Rhipicephalus*) in the *Ixodidae* family and two genera (*Ornithodoros* and *Argas*) in the *Argasidae* family, which were sampled from 148 sites in 30 provinces, metropolises or autonomous regions of mainland China (Fig. 1a). The geographic range and distribution of the ticks varied from species to species (Supplementary Tables 1–4). The most dominant species was *Haemaphysalis longicornis*, which accounted for 39.2% and were collected from 20 of 30 provinces (Fig. 1a). Five tick species were collected from two provinces, that is, Guangxi (a southern province) and Jilin (a northeastern province). Two to three tick species were usually collected from most provinces. Tick samples were pooled according to species, sex, collection site and blood-feeding status, and the total RNA was extracted from each pool. As a result, we included 8,182 adult ticks in the study, and successfully constructed 607 RNA libraries for Illumina HiSeq sequencing.

**Extended Data Fig. 1:**
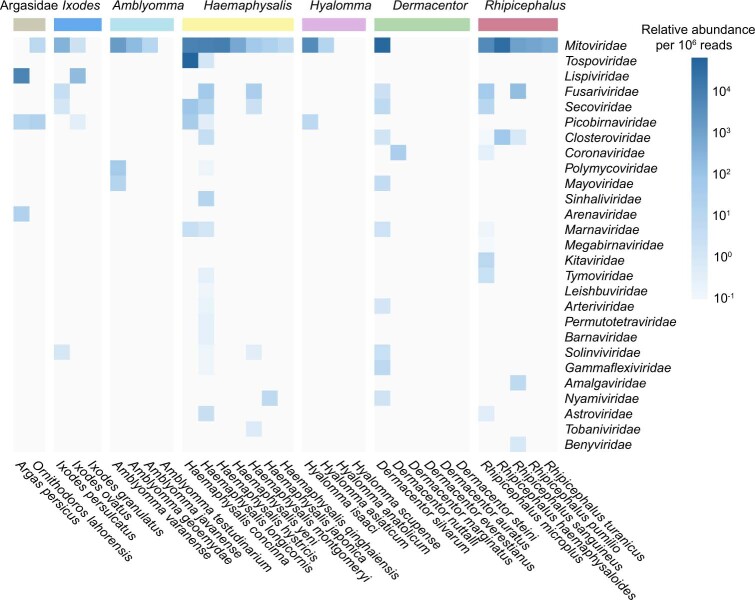
The heatmap of relative abundance for the remaining viral families.

From 46 billion 100–150 bp paired-end reads generated in the study, we de novo assembled 207 million contigs after removing rRNA reads, using previously described methods with slight modifications (Online Methods)^17^. We excluded endogenous virus elements, bacteriophage, retrotransposons and DNA viruses from our study. Viral RNA reads accounted for 3.5 × 10^–7^–13.6% of the total reads within each library, with an interquartile range (IQR) of 0.03%–0.3% (Fig. 1b). We subsequently had a total of 116,359 contigs assigned to RNA viruses, from which a total of 1,801 complete or nearly complete viral genomes were assembled. The completeness of viral genomes is listed in Supplementary Table 6. These contigs shared identities either with 59 established RNA viral families, which are known to infect vertebrates, invertebrates, plants and fungi, or with a group of unclassified RNA viruses (Fig. 1c and Extended Data Fig. 1). It is possible that some viral families were from undigested blood, vegetation or body surface of animal hosts; however, the co-occurrence of many families in different tick species from various geographical locations provided strong evidence for their association with ticks. The reads belonging to the nine common viral families, including *Rhabdoviridae*, *Totiviridae*, *Phenuiviridae*, *Partitiviridae*, *Botourmiaviridae*, *Chuviridae*, *Orthomyxoviridae*, *Peribunyaviridae* and *Narnaviridae*, were highly abundant, accounting for 25%–42% of total viral reads in the eight tick genera. Five viral families (*Nairoviridae*, *Flaviviridae*, *Reoviridae*, *Chrysoviridae* and *Deltaflexiviridae*) were common only in hard (ixodid) ticks, while *Rhabdoviridae*, *Partitiviridae* and *Chuviridae* were abundant in soft (argasid) ticks (Fig. 1d). Soft ticks differ from hard ticks in their endophilic behaviour and usually live near their favourite host (in its burrows, nests or resting areas)^18^, which may correlate with their different virome composition. Among the top 32 RNA viral families identified in ticks here, 23 (78%) had also been reported in mosquitos, nine (28%) in biting midges and seven (21%) in mites, indicating the similarity in virome structures among blood-sucking arthropods (Fig. 1e).

### Factors shaping the tick virome

**Fig. 2:**
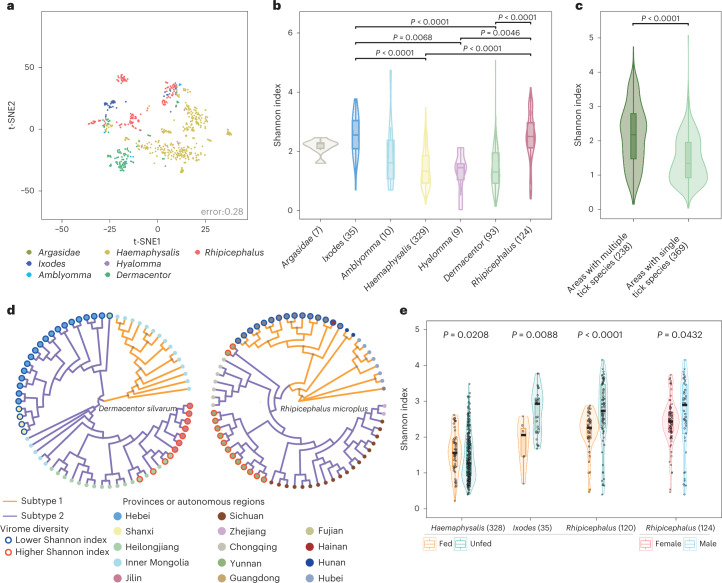
Factors shaping the tick virome. **a**, Between-group clustering of viromes among ticks in the family *Argasidae* and six genera of the family *Ixodidae* by t-SNE analysis. **b**, Shannon indexes of α diversity of tick viromes. Statistical significance was determined by two-sided Kruskal–Wallis test for multiple comparison, with *P* values adjusted by the Bonferroni method. **c**, Shannon indexes of tick viromes were compared between areas with two or more tick species and those with single tick species within a radius of 50 km. The *P* value was determined by two-sided Mann–Whitney U test. **d**, Maximum-likelihood phylogeny of genetic populations for *Dermacentor silvarum* and *Rhipicephalus microplus*. Lineages of each tick species were classified on the basis of nuclear single-nucleotide polymorphisms from the transcriptome data. Bootstrap values above 0.8 are indicated by grey asterisks. The outer colour of dots represents virome diversity: red, higher Shannon index; blue, lower Shannon index. The inner colour of dots indicates the location of the tick collection. **e**, Shannon indexes of tick viromes were compared between fed and unfed ticks of *Haemaphysalis*, *Ixodes* and *Rhipicephalus* genera. The indexes were compared between female and male ticks of the genus *Rhipicephalus*. The *P* value was determined by two-sided Mann–Whitney U test. Sample numbers are marked in brackets. Boxplot elements: centre line, median; box limits, upper and lower quartiles; whiskers (error bars), the highest and lowest points within 1.5× interquartile range of the upper and lower quartiles. Source data

**Extended Data Fig. 2:**
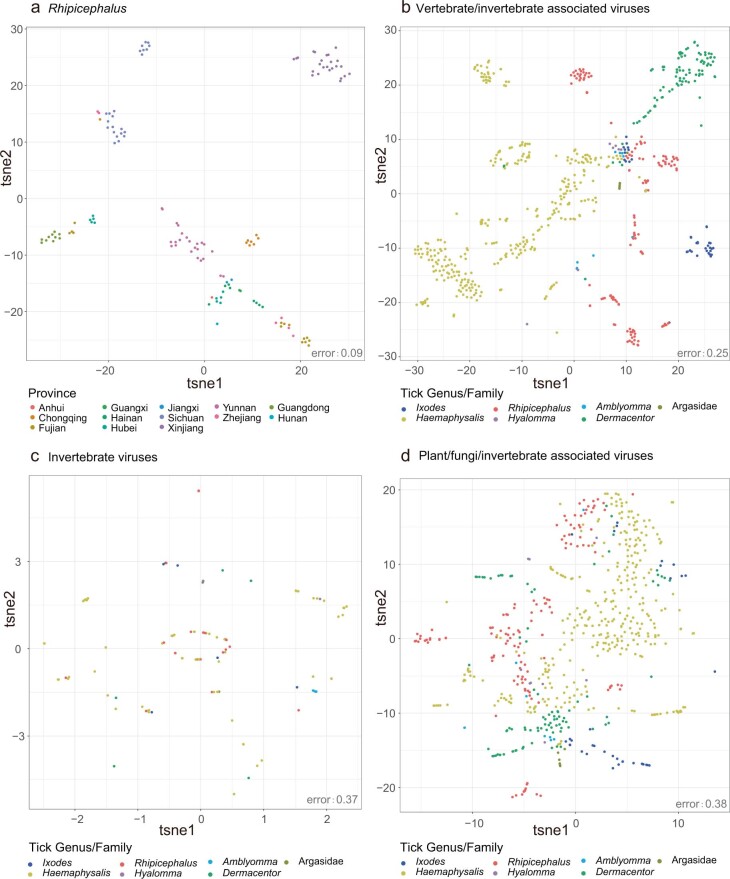
tsne analysis of (a) Rhipicephalus; (b) vertebrate/invertebrate associated viruses, invertebrate specific viruses, and plant/fungi/invertebrate associated viruses.

**Extended Data Fig. 3:**
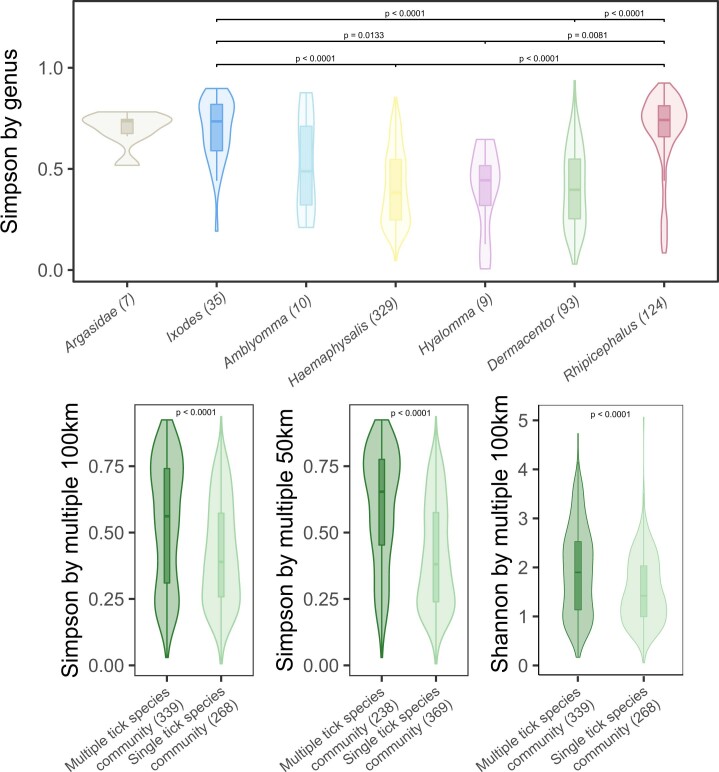
Alpha diversity of tick virome among different genera and multiple/single communities. **a**, Statistical significance was determined by two-sided Kruskal–Wallis test for multiple comparison with p values adjusted by the Bonferroni method. **b**, The p value was determined by two-sided Mann–Whitney U test. Boxplot elements: centre line, median; box limits, upper and lower quartiles; whiskers (error bars), the highest and lowest points within 1.5 interquartile range of the upper and lower quartiles. Source data

**Extended Data Fig. 4:**
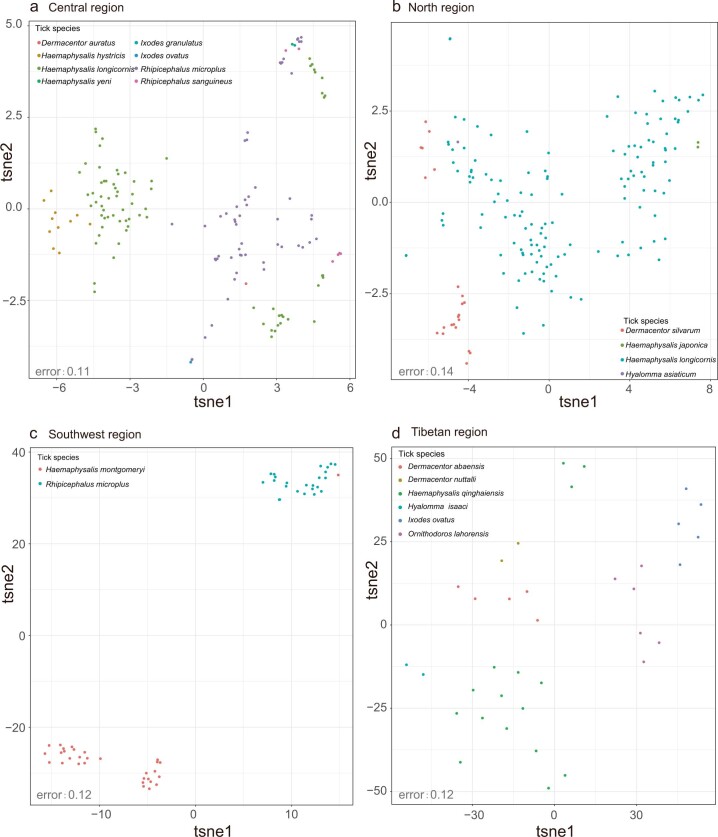
tsne analysis of tick virome of same ecogeographical regions.

The large-scale meta-transcriptomes from a wide range of tick species across various geographic locations allowed us to investigate potential vector-associated evolutionary and ecological factors contributing to the diversity of tick viromes, which were largely unknown. We used operational taxonomic units (OTUs) to characterize the viral diversity of our tick samples, considering a large amount of unclassified novel viruses. We showed that the tick virome was distinctive among different genera of hard ticks using t-SNE (t-distributed stochastic neighbour embedding) (Fig. 2a). The disparate virome configurations within the genus *Rhipicephalus* could be further clustered by ecogeographical distribution of the tick samples (Extended Data Fig. 2a). An in-depth investigation revealed that the different tick genera had different compositions of vertebrate-associated viruses, but they shared more similar compositions of either invertebrate-associated, or plant/fungi-associated viruses (Extended Data Fig. 2b). This implies that the community structures of vertebrate-associated viruses are constrained at the tick genus level. Furthermore, ticks in the genus *Ixodes* and *Rhipicephalus* had significantly higher α diversity of the tick virome than other tick genera (Kruskal–Wallis test, *P* < 0.05) (Fig. 2b and Extended Data Fig. 3). *Ixodes* is the largest genus of hard ticks with over two hundred species. This implies that the number of viruses maintained by animals increases with animal species richness^19^. The high species number of *Ixodes* might partially explain its high virome diversity. *Rhipicephalus* includes species with global distribution, such as *R. microplus* and *R. sanguineus*^20^. Their high virus diversity might be associated with their invasive behaviour and extensive infestation^21^. However, the virome pattern between different tick species was not discriminable unless under the same ecogeographic regions (Extended Data Fig. 4), suggesting unknown factors complicating compositional variations in viromes across tick species.

Interestingly, when the ecogeographical distribution of ticks was studied, we found significantly higher virome diversities in areas with two or more tick species within the radius from 10 to 200 kilometers (km) than those with only single tick species (Mann–Whitney U test, *P* < 0.001) (Fig. 2c: 50 km radius; Extended Data Fig. 3). One possibility is that a region with more species of animals and plants might have more species of ticks, leading to more species of tick-associated viruses. Another possibility is that the greater viral diversity observed in areas with two or more tick species might be due to the inclusion of a tick species with a more diverse virome. It warrants further study whether this phenomenon is a plausible result of intricate ecological interaction between hosts, vectors and viruses in shared habitats of different tick species.

Our previous genomic investigation of six major tick species reported that genetic lineages of ticks had different bacterial loads^22^, but little is known about their association with viral diversity. On the basis of nuclear single-nucleotide polymorphisms called from the meta-transcriptome data^22^, both *Dermacentor silvarum* and *R. microplus* were classified into two lineages in this study. One lineage of *D. silvarum*, mainly from Hebei province, had lower virome diversity than the other from Jilin province (Mann–Whitney U test, *P* < 0.01). A similar pattern was observed in *R. microplus*, where lineage 1 ticks sampled from the Guangdong province had lower virus diversity than lineage 2 ticks from Yunnan province (Mann–Whitney U test, *P* < 0.01) (Fig. 2d**)**. Despite the significant difference in virome diversity between the two genetic lineages of a tick species, it should be noted that the genetic difference of a tick species doesn’t necessarily determine its virome diversity. The vertebrate host distribution in different regions or provinces has not only contributed to the presence of tick lineages, but also plays an indispensable role in the tick virome. We also detected differential gene expression between the lineages with significantly different virome diversity and found that the expression of genes involved in immune pathways, such as complement activation, were significantly lower in ticks with greater virome diversity (Kolmogorov–Smirnov test, *P* < 0.01). Complement-like molecules are known to play an important role in immune responses against bacteria and viruses in ticks^23^. These findings indicate that the tick lineages with suppressed innate immunity might better tolerate various viruses, thus leading to more diverse viromes. Further studies are required to validate the association between the tick immune system and virome diversity, which may pave a broad avenue for virus inference via genetic manipulation.

**Extended Data Fig. 5:**
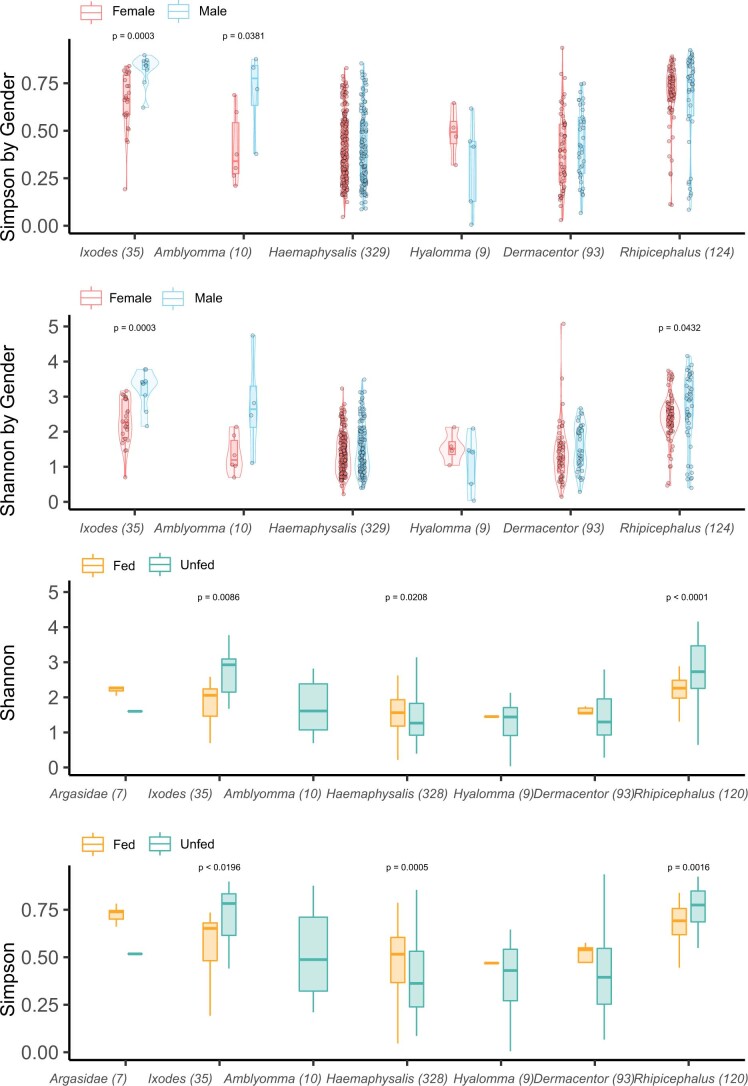
Alpha diversity of tick virome of each genus by gender and feeding status. The p value was determined by two-sided Mann–Whitney U test. Boxplot elements: centre line, median; box limits, upper and lower quartiles; whiskers (error bars), the highest and lowest points within 1.5 interquartile range of the upper and lower quartiles. Source data

We subsequently explored individual factors that possibly contribute to the virome component of ticks (Extended Data Fig. 5). Fed ticks in the genus *Haemaphysalis* had significantly higher virome diversity than unfed ticks (Mann–Whitney U test, *P* < 0.05). By contrast, fed ticks in the genus *Ixodes* and *Rhipicephalus* had significantly lower virome diversity than unfed ticks (Mann–Whitney U test, *P* < 0.01). Theoretically, fed ticks should at least have similar diversity as unfed ticks because fed ticks can also acquire additional viruses during the blood meal. The impact of blood-feeding on virome diversity seemed to vary between tick genera. Whether the lower virome diversity of fed *Ixodes* and *Rhipicephalus* ticks may be due to some kind of inhibition from the host blood, such as antimicrobial peptides (AMP) and complement, deserves further investigation. The virome diversity of male ticks in the genus *Rhipicephalus* was significantly higher than those of females (Mann–Whitney U test, *P* < 0.05) (Fig. 2e).

### Evolutionary history of tick-associated RNA viruses

**Fig. 3:**
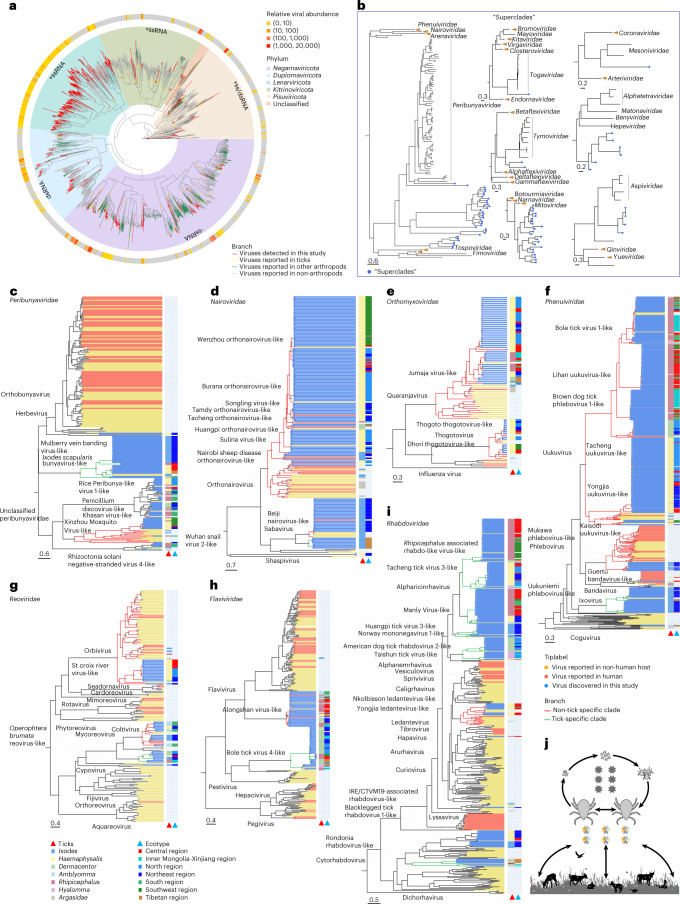
Phylogenetic analysis of tick-associated viruses. **a**, The maximum-likelihood phylogeny of RNA viruses of five phyla. The outside circle represents the relative abundance estimated by read counts per million non-rRNA reads. Parental nodes of viral families are marked with yellow asterisks, while families in which tick viruses possessed a more ancestral position than other arthropod viruses are marked with red asterisks . **b**, Order-level phylogenic trees of seven RNA virus ‘superclades’ distinct from the defined viral families. **c**–**i**, Family-level phylogenic trees of seven arboviral families. Scale bar, amino acid substitutions per site. Bootstrap values above 0.8 are shown with grey asterisks. The phylogenetic clades of viruses only found in ticks (‘tick-specific clades’) are coloured green, while those of viruses whose host range includes vertebrates and ticks (‘non-tick-specific clades’) are highlighted in red. **j**, The ecosystem of the tick’s life cycle and transmission of tick-associated viruses. The grey viruses inside the tick life cycle represent tick-specific viruses. The multicolour viruses between tick and animal hosts or living in the environment indicate non-tick-specific clades of viruses.

The phylogeny of five major RNA virus phyla was constructed on the basis of RNA-dependent RNA polymerase (RdRp) domain to understand the evolution of tick-associated viruses (Fig. 3a). The overall tree topology showed that the viruses detected in this study dispersed in all five phyla including *Pisuviricota* (double-stranded (ds)RNA and positive single-stranded (+ss)RNA), *Kitrinoviricota* (+ssRNA), *Lenarviricota* (+ssRNA), *Negarnaviricota* (negative single-stranded (−ss)RNA) and *Duplornaviricota* (dsRNA). In particular, 41.5% of the viruses were +ssRNA viruses, 28.5% were −ssRNA viruses, 1.6% were dsRNA and 28.3% were unclassified viruses, suggesting the specific preference for +ssRNA in the tick virome.

Majority (46.5%) of tick-associated viruses among all virus species in the phylogenetic tree (Fig. 3a) belonged to the *Lenarviricota* phylum, within which there was extensive viral diversity. *Lenarviricota* is a direct descendant of the bacteriophage, Leviviridae-like viruses, and diverged into three families (*Narnaviridae*, *Botourmiaviridae* and *Mitoviridae)*^24^. Horizontal virus transfer among distinct hosts including plants, fungi and invertebrates was proposed to play a key role in the evolutionary pathway of *Lenarviricota*^25^. Notably some tick-associated viruses formed a separate clade that was sandwiched between *Mitoviridae* and *Narnaviridae* (Fig. 3a), indicating the involvement of ticks during horizontal virus transfer. We also observed that tick-associated viruses formed the ancestry clade to *Aspiviridae* and *Peribunyaviridae* families of −ssRNA viruses.

To further study the evolutionary history of tick-associated viruses, we included viral RdRp sequences from other arthropods (Fig. 1e) and compared their phylogenetic distances to the root of families that they were derived from. We found that tick viruses branched off from more ancient positions compared with other arthropod viruses (11 out of 19 viral families), including 5 of 6 +ssRNA virus families, 3 of 9 −ssRNA virus families and 3 of 4 dsRNA virus families (Fig. 3a and Supplementary Table 5). These findings indicate that tick-associated viruses possess a basal position in the evolutionary history of RNA viruses, especially for +ssRNA viruses. More importantly, we provide further evidence that arthropods play a largely unnoticed role in the early evolution of viruses among terrestrial animals, although it is believed that RNA viruses originated from the ocean ecosystem^26^.

From the meta-transcriptome data, we identified a total of 724 RNA viruses (Supplementary Table 6). Among them, 223 viruses shared over 90% amino acid similarities of the RdRp domain with known viruses, around 70% of which had never been identified in ticks before. Notably, about 8% of the viruses in ticks had been reported in other arthropods, including moths, bees, flies, spiders and mosquitos (Supplementary Table 7). It is usually thought that the viral species in one kind of arthropod have rarely been detected in other kinds. The presence of a few other arthropod-associated viruses in ticks is interesting and warrants further exploration. The remaining 501 RNA viruses share 22.4–89.9% (mean: 59.1%) amino acid identities to known viruses and form separate monophyletic lineages that diverged at different viral taxonomic levels. Among those putative novel viruses, 467 fell into 59 currently defined families, while the other 34 divergent RNA viruses were sufficiently distinct from other known family or order lineages in the phylogenies, and were collectively grouped into 7 putative ‘superclades’ with the currently defined virus orders, families and floating genera. These novel viruses were mostly sibling to known families (for example, *Mitoviridae*, *Togaviridae, Tymovoridae*, *Mesoniviridae, Hepeviridae*), except for two groups that were ancestral to *Aspiviridae* and *Perbunyaviridae* (Fig. 3b). The discovery of the divergent viruses fills in the missing pieces of RNA virus diversity and highlights the role of ticks in virus divergence.

We then constructed family-level phylogenies on the basis of the RdRp domain to better characterize tick-associated viruses (Supplementary Fig. 1). We found that most of the highly abundant viruses were phylogenetically dispersed in the well-known arbovirus families (including *Peribunyaviridae, Nairoviridae, Phenuiviridae, Orthomyxoviridae, Flaviviridae, Reoviridae* and *Rhabdoviridae*) that contained zoonotic or epizootic pathogens (Fig. 3c–i). It was particularly interesting that tick-associated viruses occupied the ancestral position among the classical arbovirus genera, as exemplified by orthobunyavirus, phlebovirus, sabavirus and coltivius (Fig. 3c–f).

Importantly, the above phylogenies revealed that the tick-associated viruses in each family (except for family *Orthomyxoviridae*) could generally be classified into two categories (Fig. 3c–i). A category of viruses had so far only been detected in ticks and form separate phylogenetic clades (denoted as ‘tick-specific clades’) in each of the viral families. The other category of viruses, clustering in distinct clades, was not only identified in ticks but was also previously reported in various animals (denoted as ‘non-tick-specific clades’). We found that the tick viruses in non-tick-specific clades had a higher Shannon index of tick species than the viruses in tick-specific clades in 4 out of 6 virus families including *Flaviviridae, Nairoviridae*, *Peribuynaviridae* and *Phenuiviridae*, and a higher Shannon index of ecotypes in 3 out of 6 virus families (Supplementary Table 8). In addition, the viruses in tick-specific clades had a lower phylogenetic association index with tick species than those in non-tick-specific clades (*P* < 0.05), indicating significant clustering and stronger co-divergence between viruses and tick species (Supplementary Table 8).

### Genomic characteristics of tick-associated RNA viruses

**Fig. 4:**
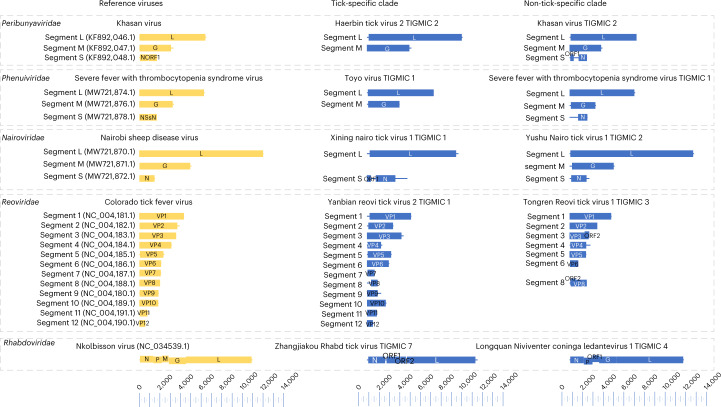
Genome structure of tick-associated viruses. The genomes are drawn to a unified length scale. Reference viruses and viruses discovered in this study are highlighted in yellow and blue, respectively. The bottom scale indicates the length of nucleotides. L, RNA-dependent RNA polymerase; G, glycoprotein; M, matrix protein; N, nucleoprotein; VP, viral protein; P, phosphoprotein.

**Extended Data Fig. 6:**
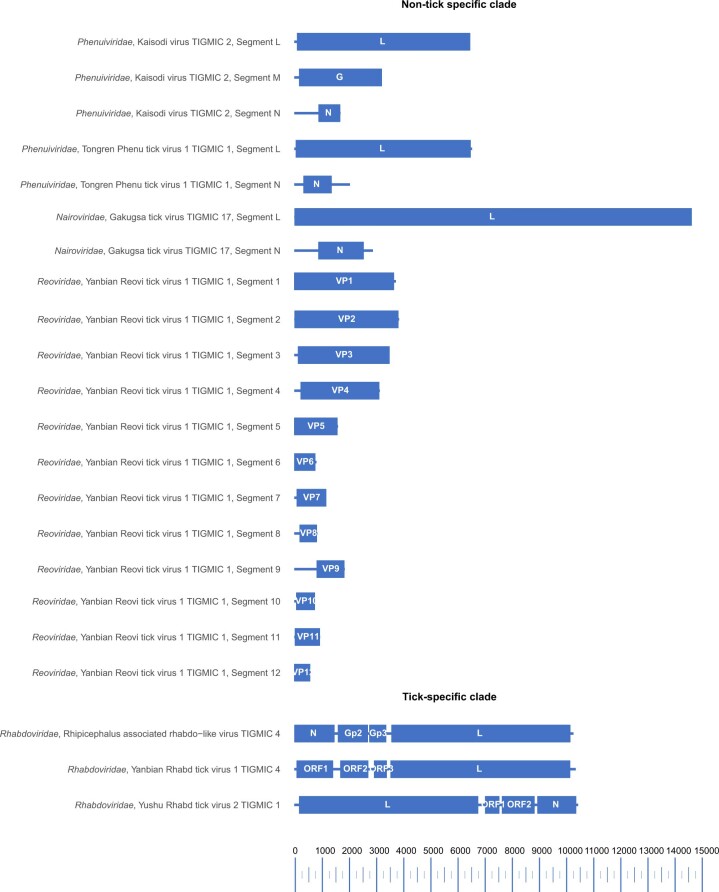
Genome structure of ‘non-tick-specific clades viruses’ and ‘tick-specific viruses’ detected in this study. The genomes are drawn to a unified length scale. Reference viruses and viruses discovered in this study are highlighted with yellow and blue respectively. The bottom scale indicates the length of nucleotides. L: RNA-dependent RNA polymerase; G: glycoprotein; M: matrix protein; N: nucleoprotein; VP, viral protein; P: phosphoprotein; C: coat/capsid protein.

**Extended Data Fig. 7:**
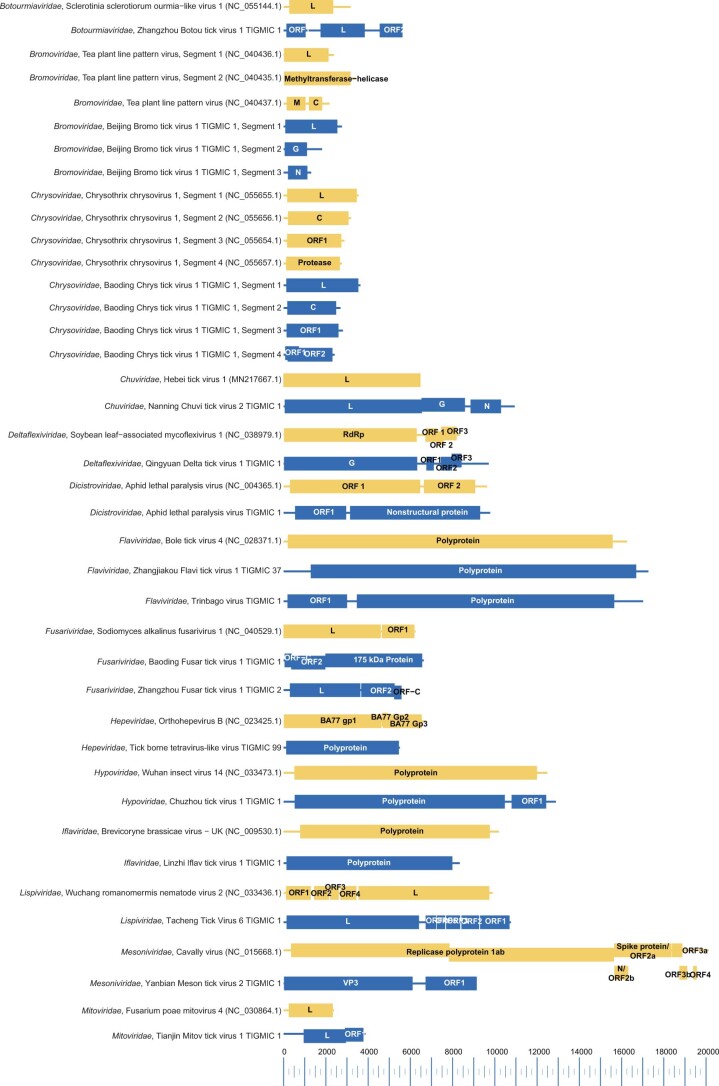
Genome structure of representative viruses for viral families detected in this study. The genomes are drawn to a unified length scale. Reference viruses and viruses discovered in this study are highlighted with yellow and blue respectively. The bottom scale indicates the length of nucleotides. L: RNA-dependent RNA polymerase; G: glycoprotein; M: matrix protein; N: nucleoprotein; VP: viral protein; P: phosphoprotein; C: coat/capsid protein.

**Extended Data Fig. 8:**
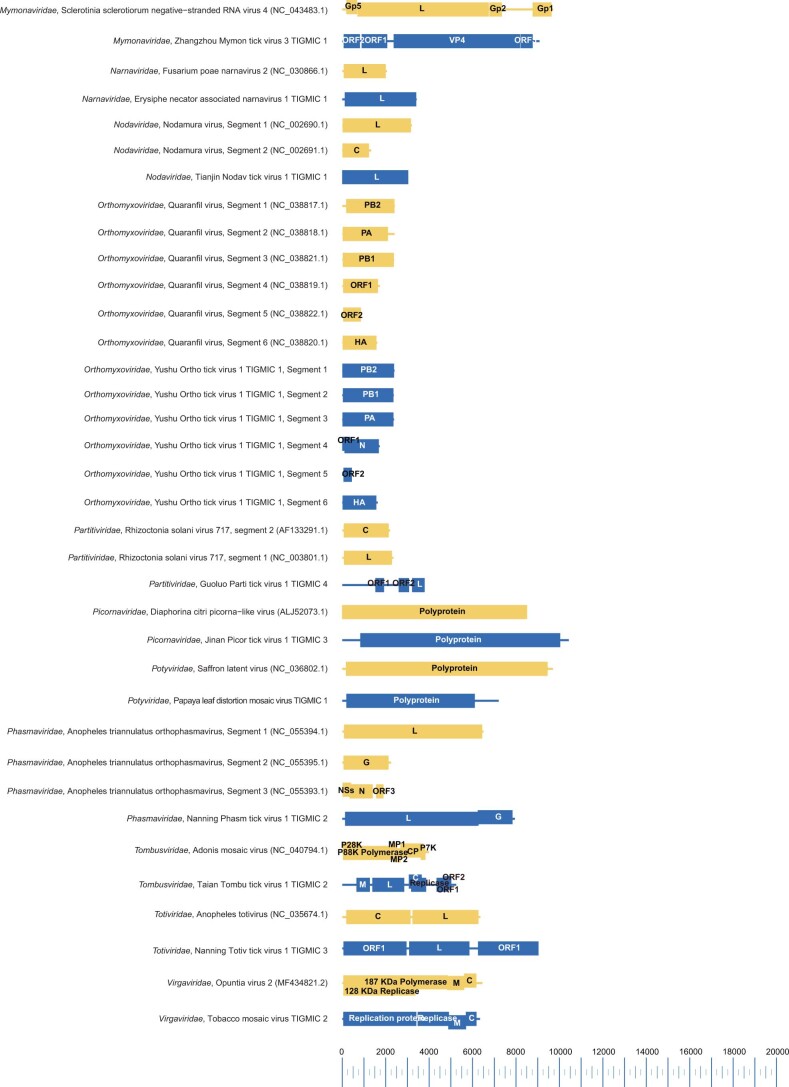
Genome structure of representative viruses for viral families detected in this study. The genomes are drawn to a unified length scale. Reference viruses and viruses discovered in this study are highlighted with yellow and blue respectively. The bottom scale indicates the length of nucleotides. L: RNA-dependent RNA polymerase; G: glycoprotein; M: matrix protein; N: nucleoprotein; VP: viral protein; P: phosphoprotein; C: coat/capsid protein.

**Extended Data Fig. 9:**
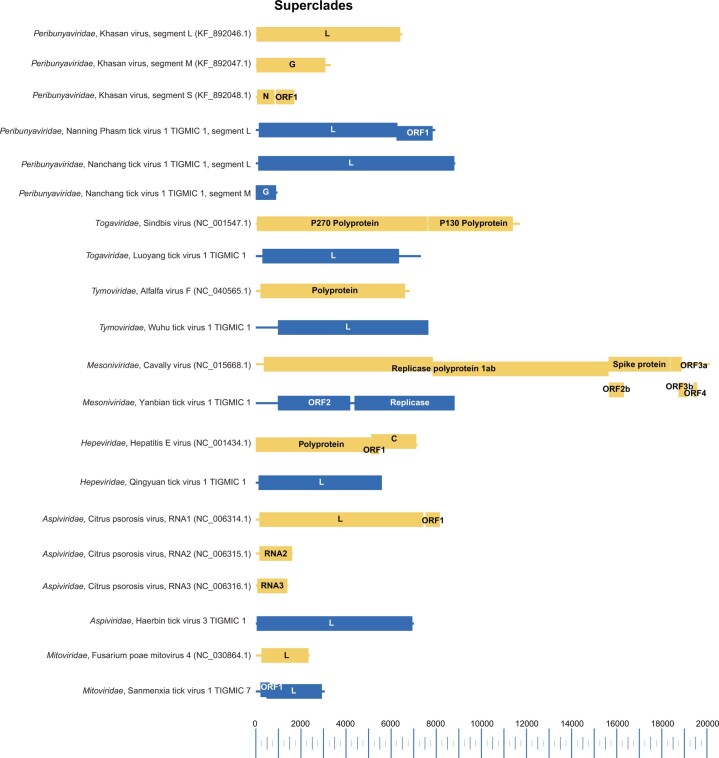
Genome structure of viruses classified into superclades detected in this study. The genomes are drawn to a unified length scale. Reference viruses and viruses discovered in this study are highlighted with yellow and blue respectively. The bottom scale indicates the length of nucleotides. L: RNA-dependent RNA polymerase; G: glycoprotein; M: matrix protein; N: nucleoprotein; VP: viral protein; P: phosphoprotein; C: coat/capsid protein.

Putative genome annotation of the assembled 1,801 complete or nearly complete (Supplementary Table 6) viral genomes summarized here showed a wide diversity of genome architectures (Extended Data Figs. 6–9). Although most of the tick virus genome organizations were similar to those of invertebrates viruses^27–29^, some of them exhibited extensive variation. Notably, segment or gene loss was mostly observed in tick-specific clades (8/10 clades), such as the lack of glycoprotein or nucleoprotein in the *Phenuiviridae*, *Nairoviridae* and *Peribunyaviridae* families, and the loss of non-structural genes in *Rhabdoviridae* (Fig. 4). However, only 2 out of 12 non-tick-specific clades had segment loss. These findings imply that the resultant genome deficiency in the tick-specific clade might have limited the viral infectivity to a narrower host range, for instance, due to the lack of glycoprotein for attaching to host receptors. Furthermore, we also found that the gain of non-structural genes mostly occurred in +ssRNA (*Botourmiaviridae*, *Fusariviridae*, *Mitoviridae*, *Tymoviridae* and *Virgaviridae*) and dsRNA (*Partitiviridae* and *Totiviridae*) viruses. Among them, *Botourmiaviridae*, *Mitoviridae* and *Totiviridae* showed longer genome length, especially in replicase-related genes, some of which were strikingly twice the size of previously documented viruses (Extended Data Figs. 7 and 8).

### Human pathogenic tick-associated RNA viruses

**Extended Data Fig. 10:**
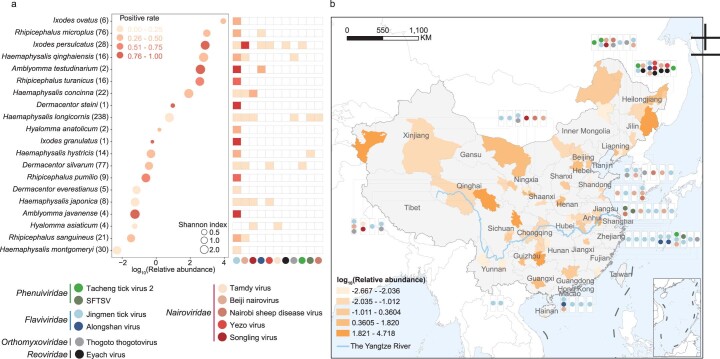
Epidemiology and human infection potential of tick-associated viruses. **a**, The abundances and positive rates of 11 known tick-borne RNA viral pathogens in different tick species collected in this study. The tick species are sorted by the relative abundance of detected known viral pathogens, which were estimated by read counts per million non-rRNA reads. The color of circles indicates the overall positive rate of 11 known tick-borne viral pathogens, while the color of square indicates the positive rate of each virus detected in tick species respectively. The size of circles represents the Shannon index of 9 detected viral families (*Flaviviridae*, *Nairoviridae, Phenuiviridae, Rhabdoviridae, Peribunyaviridae, Reoviridae, Orthomyxoviridae, Chuviridae* and *Nodaviridae*). **b**, Geographic distribution of 11 known tick-borne viral pathogens in China.

We assembled 11 (including 1 dsRNA, 2 +ssRNA and 8 −ssRNA) viruses known to be pathogenic to humans in 20 (64.5%) tick species. Among these 11 viruses, Yezo virus and Eyach virus, which are viral pathogens circulating in Japan^30^ and Europe^31^, were first detected in China. In most cases, tick-associated human viral pathogens are harboured by multiple tick species (Extended Data Fig. 10a). Nairobi sheep disease virus, which has been reported to infect humans working in the laboratory^32^, was identified in *H. longicornis* in a wide range of areas. However, Tamdy virus and Eyach virus, which on the basis of serological surveys have been known to infect humans, were only found in *Hyalomma asiaticum* and *I. persulcatus* ticks at restricted geographical regions of northwestern and northeastern China, respectively^33,34^ (Extended Data Fig. 10b). We propose that other factors, such as tick and host densities, contact between tick and human or virus–host interaction, might contribute to the likelihood of cross-species transmission. Notably, more viral pathogens were detected in ticks from northeastern China compared with other areas (Extended Data Fig. 10b). The complex ecological niches of ticks might contribute to the emergence of various pathogens in this region because the biodiversity of the hosts and vectors could impact viral transmission^35,36^.

## Discussion

We analysed the tick RNA virome present in 31 tick species from two tick families across mainland China using meta-transcriptomics. We detected reads with sequences homologous to 59 RNA viral families (Fig. 1c), which at least triples the reported viral families present in ticks worldwide^37^. Ticks developmental stages include larva, nymph and adult, and each stage sucks blood from animal hosts that include mammals, birds, reptiles and amphibians. Their life cycle enables ticks to harbour a reservoir of diverse ds and ssRNA viruses that have the potential to infect a broad range of hosts. It has been suggested that vertebrate and plant viruses, especially in negative-sense RNA viruses, emerged from the arthropod virome^27^. Using our evolutionary analyses, we report that ticks have developed RNA virus reservoirs after long-term interaction with different viruses from multiple hosts.

The finding that viruses in non-tick-specific clades tend to be found in more diverse tick species and ecotypes is interesting, and suggests that some tick viruses have evolved into generalists, with suitable genetic architectures and plasticity towards better tolerance of different ticks and hosts compared with specialist viruses in the tick-specific clades.

Mosquito-borne viruses also evolve towards generalization or specialization^16,38^. Some viruses, such as the Chikungunya virus^39^, are probably generalists that have evolved under conditions of host breadth and increased infectivity to vertebrate hosts, while others are specialists that benefit from adaptation to a single host^40^.

The tick RNA-virome composition, especially that of vertebrate-associated viruses, was distinctive at the genus but not at the species level of ticks. Biological characteristics as well as life cycle traits of different tick genera might have determined their host preference or ecology, and subsequently shaped their virome after long-term adaptation. Viruses present in various tick species or in diverse ecotypes are probably more capable of infecting multiple hosts. However, additional factors that predispose tick-associated viruses to a variety of animals should be further explored as some tick-borne pathogens were detected only in a single tick species. The tick virome dataset generated in our study should benefit the community and enable in-depth study of tick-associated RNA viruses.

## Methods

### Sample collection

From March 2016 to October 2019, ticks were collected from 30 provinces, metropolises or autonomous regions of mainland China. The collection sites were selected according to their ecological environments, including coniferous forest, steppe, farmland, desert, shrubland and tropical forest. Ticks were collected by dragging a standard 1 m^2^ flannel flag over vegetation or from domestic or wild animals such as cattle, dogs, sheep, goats, cats, rabbits, camels, deer and boars. The latitude and longitude of each collection site were recorded. The species, gender and developmental stage of each tick were identified by entomologists. Adult ticks were used for meta-transcriptomics analysis, including 31 tick species (Fig. 1a) from the family *Argasidae* (2 genera) and *Ixodidae* (6 genera). Live ticks were transported to the laboratory and thoroughly sterilized (two successive washes of 70% ethanol for 30 s), then stored at −80 °C. Ticks were divided into pools on the basis of tick species, gender, sampling sites and blood-feeding status.

### RNA preparation and sequencing

Extraction of total DNA and RNA from pools of ticks was performed using the AllPrep DNA/RNA mini kit (Qiagen) with modifications. Briefly, ticks were homogenized in RLT solution under liquid nitrogen. The homogenate was then incubated at 55 °C for 10 min with proteinase K (Qiagen) and centrifuged for 30 s at 15,000 *g*. The homogenized lysate was transferred to an AllPrep DNA spin column and centrifuged for 30 s at 8,000 *g*. The AllPrep DNA spin column was used for later DNA purification, and the flow-through was used for RNA purification following the manufacturer’s instructions. The extracted DNA was set aside for later use (described elsewhere).

The extracted RNA was quantified using Qubit 4.0 fluorometer, and RNA quality was assessed using an Agilent Bioanalyzer 2200 (Agilent). The ribosomal RNA was removed using RiBo-Zero Gold rRNA removal reagents (Human/Mouse/Rat) (Illumina). Then, the sequencing library was prepared following the Illumina standard protocol. Paired-end (2 × 150 bp) transcriptome sequencing (RNA-seq) of the RNA library was performed on an Illumina HiSeq 4000 platform at Novogene Tech.

### Discovery and assembly of viral genomes

Raw sequencing reads were first subjected to adapter removal and quality trimmed using Trimmomatic v0.39 programme^41^. Clean reads were de novo assembled using the Trinity v2.8.5 programme^42^. All assembled contigs with length above 200 bp were subjected to BLASTN against all non-redundant nucleotide databases using a local BLAST tool^43^ and BLASTX against all non-redundant protein databases downloaded from GenBank using DIAMOND^44^ v0.9.24. A significant hit was defined by an *E*-value smaller than 1 × 10^−6^. All contigs that shared homology with the virus were kept for second-round filtering to eliminate endogenous viral elements. All putative viral contigs that had overlapping regions (>100 bp) and a threshold value of 95% similarity were first merged using the SeqMan programme of Lasergene package v7.1 (DNAstar)^45^. Then, all re-merged contigs were compared with a custom host reference database that included:

1. Whole genomes of six tick species:
  *Ixodes persulcatus* (GWHAMMH00000000);
  *Haemaphysalis longicornis* (GWHAMMI00000000);
  *Dermacentor silvarum* (GWHAMML00000000);
  *Hyalomma asiaticum* (GWHAMMK00000000);
  *Rhipicephalus sanguineus* (GWHAMMM00000000);
  *Rhipicephalus microplus* (GWHAMMN00000000);
2. Whole-genome shotgun database of *Ixodida* (Taxonomy ID: 6935, accession data:1/2/2021).

If aligned bases of the query contigs covered more than 50% and nucleotide similarity was higher than 85% from comparison with the above two databases, they were removed from the downstream analysis to eliminate any potential endogenous viral elements. Bacterial contigs were identified and discarded if they showed higher than 85% similarity on more than 50% aligned bases of query contigs to any bacteria from the BLASTN comparison result. In addition, we also removed any possible contaminating viral sequences from high-throughput sequencing, as previously reported^46^. Novel virus was proposed if amino acid similarity on the RdRp domain was below 90%, and subsequently confirmed by phylogenetic analyses^5,13,47^.

All putative novel viruses were provisionally denominated as the sampling site plus viral family (the first five characters), followed by ‘TIGMIC’ (the abbreviation for the Tick Genome and Microbiome Consortium) as strain name. Viruses of superclades were named using only the sampling region.

### Assignment of virus taxonomy

All above filtered viral contigs were annotated and classified on the basis of the best match from the BLASTX comparison or the best BLASTN match if a contig does not exhibit any homology from BLASTX results. The virus contigs assigned to DNA viruses and reverse-transcribing viruses were excluded from further analysis. If the best match of one viral contig was not assigned to any defined family, this contig was designated as ‘unclassified viruses’.

### Quantification of viral abundance

Ribosomal reads were subtracted from each library by mapping to the SILVA rRNA database (https://www.arb-silva.de/) using Bowtie2 v2.4.3 in case of unequal efficiency of rRNA removal during sequencing library preparation. Then the non-rRNA reads from each library were mapped against the assembled sequences using a Bowtie2 end-to-end alignment with sensitive parameters^48^. We acquired the read counts of each viral RNA from mapping results and performed within-sample normalization (reads per million/virus ratio) to compare samples across different conditions.

### Metadata review of arthropod virome

We searched PubMed and ISI (Web of Science) for articles published in English, and the WanFang database, China National knowledge Infrastructure, as well as the Chinese Scientific Journal Database for articles published in Chinese. We first summarized a list of common names and Latin names for each arthropod. Then we used the common and Latin names of each arthropod and the words ‘virus’ and ‘virome’ as search terms. We performed a secondary manual search on the references cited in these articles to extend our search to relevant articles. The viral detection approach in these articles were recorded in three categories: reverse transcription-polymerase chain reaction (RT–PCR), transcriptome or both methods. To include arboviruses without sequences, which were isolated and named in early years, we consulted the Arbovirus Catalog website (https://wwwn.cdc.gov/arbocat/), a compilation of biological information that has no sequence database of arboviruses. We extracted the information from the Arbovirus Catalog, including the virus names/prototypes, original sources and natural host ranges for downstream analysis. All these related data were included in our dataset to compare tick virome diversity with those of other haematophagous or non-haematophagous arthropods (Fig. 1e).

### Analyses of tick virome diversity

The coding regions of the predicated viral open reading frames (ORFs) for each viral contig were retrieved using TransDecoder^49^ v5.5.0. The coding regions that matched RdRp genes were retained and grouped into OTUs on the basis of 95% nucleotide identity by CD-HIT^50^ v4.8.1. The number of non-rRNA reads mapping to the representative sequences of each OTU cluster were determined using Bowtie2^48^ v2.4.3. For viruses whose genome translated into one single polyprotein, we only summarized the read number mapping to the RdRp region. Viral relative abundance tables were generated by normalizing the absolute read counts using transcripts per kilobase million (TPM). Each representative OTU was assigned to a viral family on the basis of their best match as described above and further classified into vertebrate/invertebrate-associated viruses, invertebrate viruses, or plant/fungi/invertebrate-associated viruses according to the host range of their corresponding viral family. Alpha diversity (within-library virome richness) was measured using the Shannon index and Simpson index in the Python package ‘skbio’ (http://scikit-bio.org/) on the basis of the relative abundance table. Statistical differences in alpha diversity among groups of different factors were assessed by Mann–Whitney U test or Kruskal–Wallis test using SPSS^51^ v20.0. Beta-diversity (between-library dissimilarity) analysis was performed and visualized by t-SNE using ‘tsne’^52^ v0.1–3.

We used the following method to describe the ecogeographic distribution of ticks. The longitude and latitude of each tick collection site was selected as the centre, and the distance between two collection sites was calculated as:

$$\begin{array}{l}{{{\mathrm{Distance}}}} =\\ {{{\mathrm{arccos}}}}(\cos (E1 - E2) \times \cos (N1) \times \cos (N2) + \sin (N1) \times \sin (N2)) \times 6371{{{\mathrm{km}}}}\end{array}$$

where *E*1 is the sample1 longitude, *N*1 is the sample1 latitude, *E*2 is the sample2 longitude and *N*2 is the sample2 latitude.

Tick species in the area within a radius of 10–200 km (stepwise 10 km) were summarized and samples were classified into two groups: more than two tick species sharing this area of habitat and only one tick species dominating in this area. The tick virome diversity between these two groups was compared using *t*-test or Mann–Whitney U test.

### Population structures of tick genetics

Illumina reads after adaptor and quality removal were aligned to the corresponding tick reference genomes (GWHAMMH00000000, GWHAMMI00000000, GWHAMML00000000, GWHAMMK00000000, GWHAMMM00000000 and GWHAMMN0000000) using Bowtie2^48^ v2.4.3. Variant sites were called and those with quality scores ≥30 and sufficient reads (8 ≤ read depth ≤ 12, genotype rate >70%) were kept following the GATK 4.0 pipeline^53^. The nuclear genome’s phylogenetic tree was constructed using the maximum-likelihood method, with bootstrap tests (1,000 replicates) implemented in IQ-Tree^54^ v.1.6.1 on the basis of the single-nucleotide polymorphisms alignment. The Shannon index of the tick virome was compared between different tick genetic populations by Mann–Whitney U test or Kruskal–Wallis test using SPSS v20.0.

### Differential expression of the transcriptome

The expression level of each gene was obtained from the above mapping results of each library against the tick reference genome using HTSeq^55^ v0.6.0. Differentially expressed (DE) genes among the genetic populations with different virome diversity were identified using the R package ‘edgeR’^56^ on the basis of false discovery rate (FDR) ≤ 0.05 and |log_2_(fold change)| ≥ 1. Enriched Gene Ontology terms (http://geneontology.org/) of the DE genes were analysed using Kolmogorov–Smirnov test in the topGO package^57^ (FDR < 0.05).

### Analyses of virus phylogeny

The highly conserved RdRp gene was selected to construct the family-, order- and phylum-level phylogenies. The RdRp proteins of RefSeq and the metadata of host information were collected from NCBI using in-house script. Predicted viral proteins of RdRp genes were clustered into a set of ‘non-redundant’ representative sequences with the threshold of 100% similarity using CD-HIT^50^ v4.8.1. The longest representative sequence for each cluster was aligned with downloaded reference proteins belonging to the same viral family or order using the E-INS-i algorithm with the implementation of MAFFT^58^ v7.490. The phylum-level alignment was constructed by the stepwise merging of all family-level or order-level alignments using MAFFT^58^ v7.490. Ambiguously aligned regions were trimmed using TrimAl^59^ v1.2, and short contigs that did not align to reference genomes were removed before constructing the appropriate alignments for downstream phylogeny. The IQ-Tree v.1.6.1 algorithm^54,60^ was used to determine the best-fit amino acid substitution model on the basis of each multiple sequence alignment, and the maximum-likelihood phylogenetic tree was assessed with bootstrap tests (1,000 replicates). The maximum-likelihood trees were visualized using the ggtree package^61^ v3.0.4.

The phylum-level tree was used to estimate the evolutionary distance for each virus family using the Python package ETE toolkits^62^. The Q3+1.5IQR of the pairwise phylogenetic distance (the branch length between any two reference viruses) for each virus family was set as the threshold. If the branch length between the virus discovered in this study and its closest relative sequence was larger than the cut-off of the same family, this virus was identified as a ‘superclade’.

All well-classified complete RdRp proteins from arthropods were downloaded from NCBI and added to the multiple sequence alignment on the basis of their identified phylum. Phylum-level virus trees with added RdRp protein sequences were then constructed using IQ-Tree^54^ v1.6.1. For each viral family, the highest common ancestor node of viruses from ticks (reference viruses and viruses in this study) or other arthropods was first estimated on the basis of the phylogeny using in-house script. The phylogenetic distances from divergent nodes to the corresponding family node were calculated and compared to identify which node, whether ticks or other arthropods, was closer to the family root.

The phylogenies of viral families that contained adequate numbers of tick-associated viruses in this study were further analysed, including *Flaviviridae*, *Nairoviridae*, *Orthomyxoviridae*, *Peribunyaviridae, Phenuiviridae*, *Rhabdoviridae* and *Reoviridae*. The phylogenetic clades were divided at the genus level, unless those that have not been classified into any current genus were clustered based on the median phylogenetic distance at the family level using TreeCluster^63^ v1.0.3. The host ranges of tick viruses discovered in this study in each clade were identified by investigating BLASTP hits revealing above 50% identity^43^. We calculated the Shannon index to determine the diversity of tick species and ecotypes for each phylogenetic clade using the Python package ‘skbio’ (http://scikit-bio.org/). The alignments used for family phylogeny were segregated by individual clade and phylogenetic trees for each clade were inferred using LG amino acid substitution models by the Bayesian method implemented in MrBayes^64^ v3.2. The association index representative of the strength of the association between virus phylogeny and host class was estimated using the above virus phylogeny and the BaTS programme^65^.

### Annotation of viral genomes

Viral ORFs were predicted using TransDecoder^49^ v5.5.0 on the basis of two criteria: (1) the length of predicted ORFs were longer than 100 amino acids; (2) only the longest ORF was reported if a short ORF was nested entirely inside. All predicted ORFs were annotated by comparing non-redundant protein databases using BLASTP and the Conserved Domain Database by PSI-BLAST with an *E*-value threshold of 1 × 10^−5^ using BLAST tools^43^. Novel genomes were confirmed by checking reads coverage and continuity using Bowtie2^48^.

### Assessment of viral infectivity risk

To further evaluate the viral abundance of a reported human pathogenic tick-borne virus, we extracted all assembled viral contigs whose best match either from BLASTX or BLASTN was annotated as any known tick-borne pathogenic virus. In addition, the references of tick-borne pathogenic viruses were downloaded and non-rRNA reads were also mapped back to them using Bowtie2 in case tick-borne viruses without assembled contigs were neglected^48^. The pairwise identities of downloaded reference nucleotide sequences were calculated against all references of the same virus species using BLASTN^43^. The Q1-1.5IQR of pairwise identities for each virus species and the 80% coverage were both set to the cut-off determining viral contigs to be pathogenic virus from the above-extracted BLASTX or BLASTN results. The abundance of each virus species was normalized to the total non-rRNA read count of each library. The mean relative abundance and prevalence of each pathogenic virus were summarized according to each tick species or geographic region. The distribution of pathogenic viruses was geo-referenced to a Chinese map at the prefecture-level with ArcGIS^66^ 10.2 (ESRI) according to locations of the tick collection. Shannon index representative of viral diversity was estimated on the basis of the OTU95 abundance table.

### Reporting summary

Further information on research design is available in the Nature Portfolio Reporting Summary linked to this article.

## Supplementary information

Supplementary Information Supplementary Fig. 1.

Reporting Summary

Supplementary Table 1 Table 1. Sampling information of sequencing libraries by species. Table 2. Sampling information of sequencing libraries by province. Table 3. Sampling information of sequencing libraries by ecological fauna. Table 4. Sampling information of sequencing libraries by characteristics. Table 5. Comparison results of phylogenetic distance and height between virus and viral family. Table 6. Best match of BLASTX and BLASTN comparison for viruses discovered in this study. Table 7. Host information of viruses first detected in ticks in this study. Table 8. Alpha diversity of (a) tick species and (b) ecotypes, and virus–host association index (c) between ‘non-tick-specific clades viruses’ and ‘tick-specific viruses’. The association index (AI) ratio = observed association index/null association index, in which the null association index is derived from 1,000 tree-tip randomizations. A ratio closer to 0 indicates a stronger host structure. The *P* values of AI were estimated by a two-sided Bayesian tip-association significance test (BaTS) and derived from 1,000 tree-tip randomizations without adjustment for multiple comparisons. Table 9. List of Tick Genome and Microbiome Consortium (TIGMIC) members.

## Data Availability

The sequencing data have been deposited to SRA under Bioproject PRJNA841744, and the assembled virus sequences have been submitted to GenBank (https://www.ncbi.nlm.nih.gov/nuccore/ with the accession no. ON746331-ON746566, ON811696-ON813070, ON811604-ON811608, ON872591-ON872654, OP628496-OP628616) and BIGD (https://ngdc.cncb.ac.cn/gsub/) under the Project PRJCA008467 (accession no. GWHBHNN00000000, GWHBHNO00000000, GWHBHNP00000000, GWHBHNQ00000000, GWHBHNR00000000, GWHBHNS00000000, GWHBHNT00000000, GWHBHNU00000000). Source data are provided with this paper.

## Source data

Source Data Fig. 2 Statistical source data.
Source Data Extended Data Fig. 3 Statistical source data.
Source Data Extended Data Fig. 5 Statistical source data.
