# Supplementary material for: Metavirome of 31 tick species provides a compendium of 1,801 RNA virus genomes
Source: Nat Microbiol. 2023 Jan 5;8(1):162–73. doi: 10.1038/s41564-022-01275-w (PMC9816062; doi:10.1038/s41564-022-01275-w)
Supplement: Supplementary file 1 — Supplementary Fig. 1. [file 41564_2022_1275_MOESM1_ESM.pdf]

nature microbiology

Resource

https://doi.org/10.1038/s41564-022-01275-w

# Metavirome of 31 tick species provides a compendium of 1,801 RNA virus genomes

In the format provided by the authors and unedited

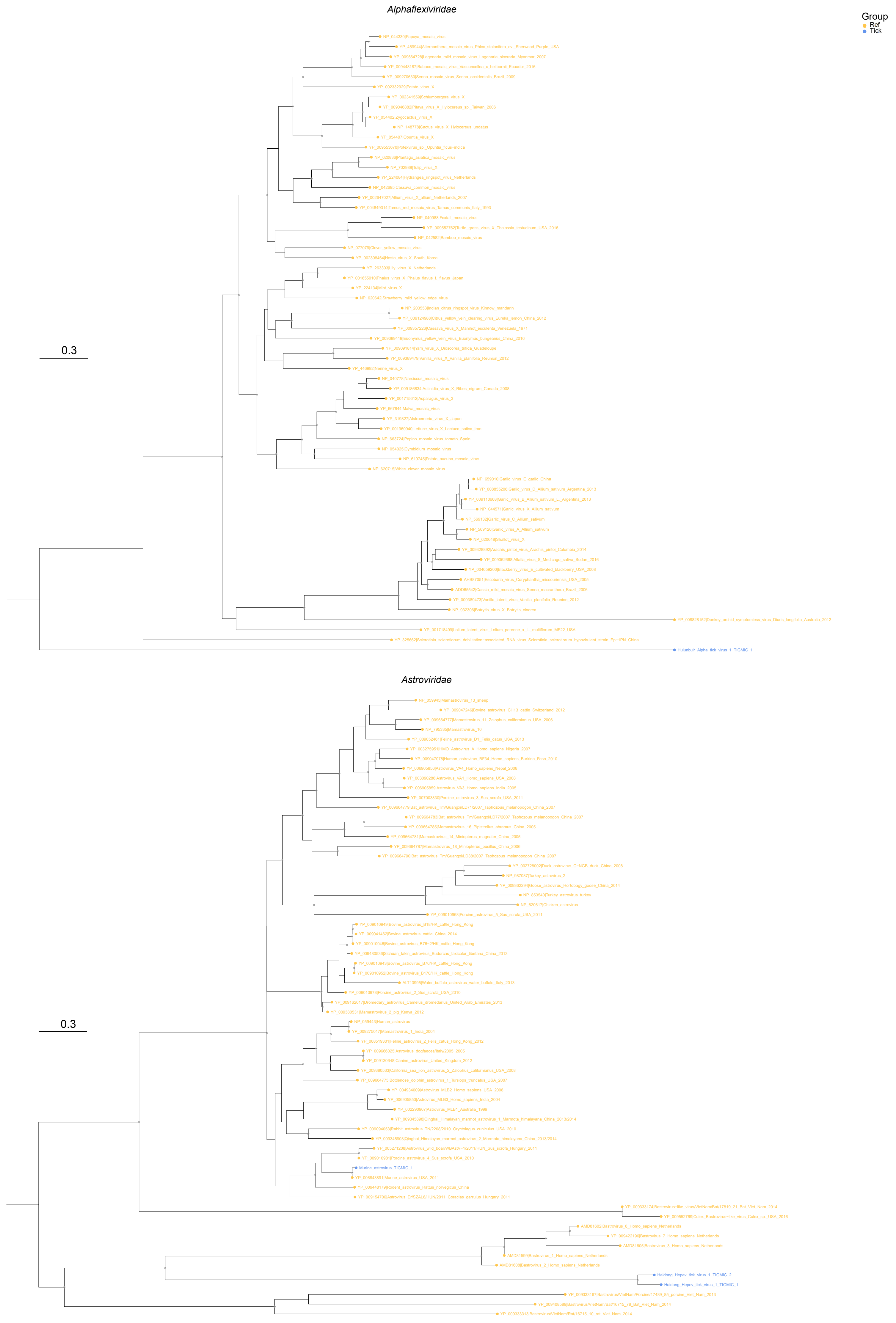
Alphaflexiviridae
Group
Ref
Tick
0.3
Hulunbuir_Alpha_tick_virus_1_TIGMIC_1
Astroviridae
0.3
Murine_astrovirus_TIGMIC_1
Haidong_Hepev_tick_virus_1_TIGMIC_2
Haidong_Hepev_tick_virus_1_TIGMIC_1

*Chrysoviridae*

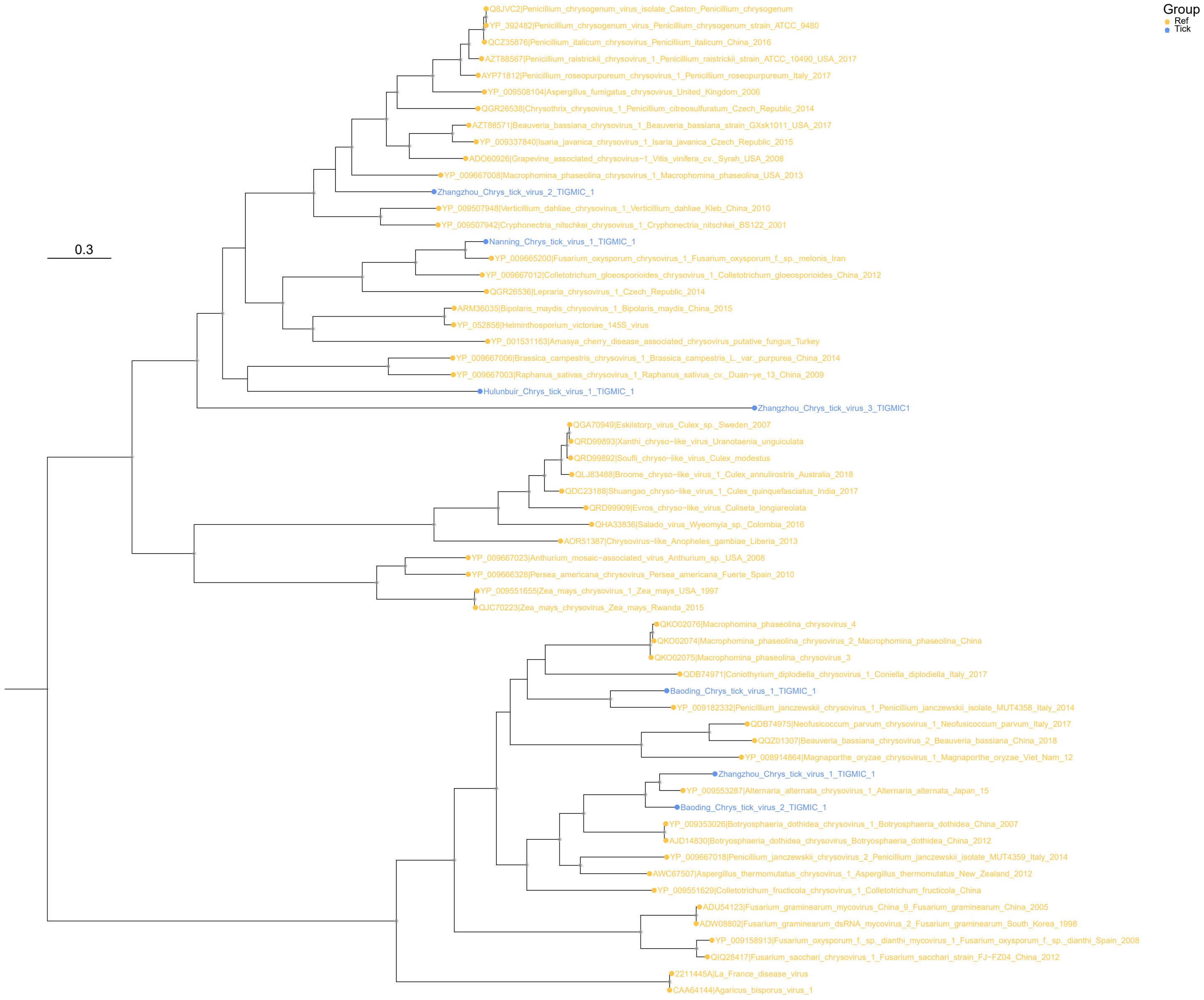


*Dicistroviridae*

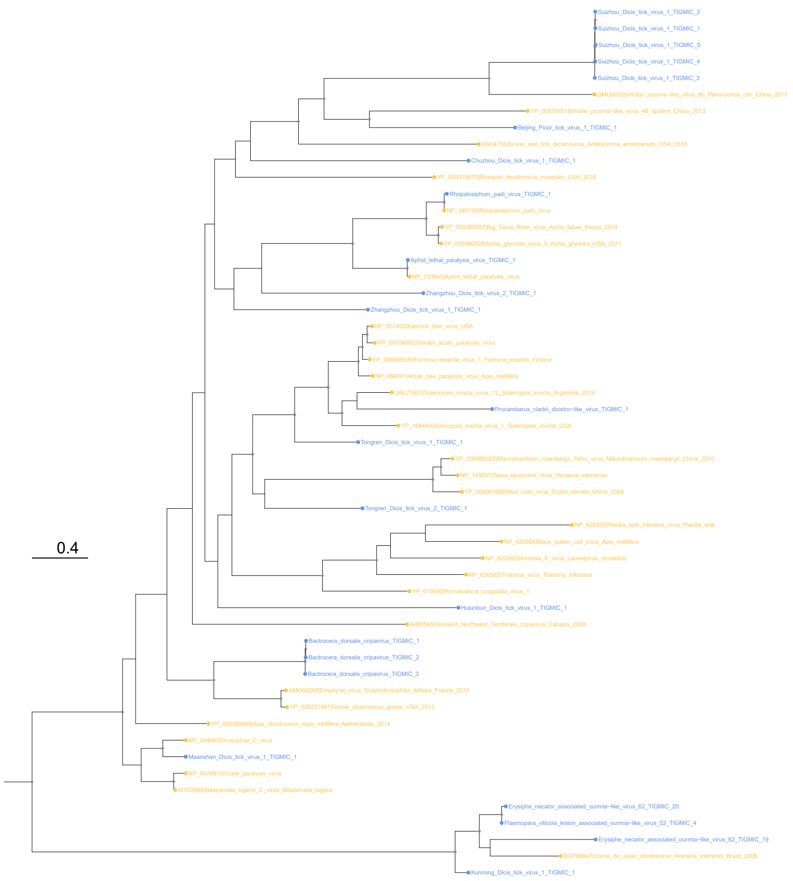

*Chuviridae*

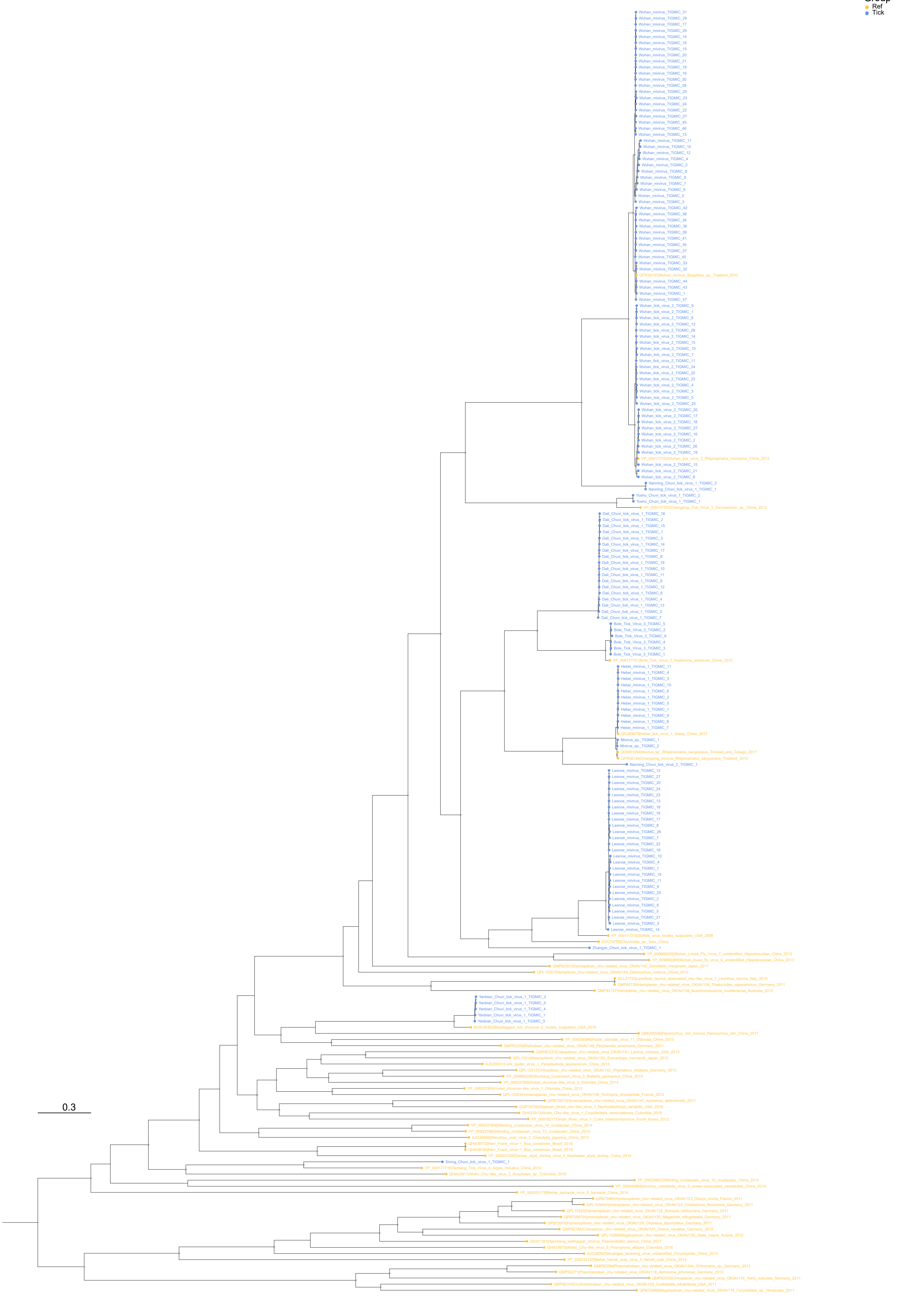

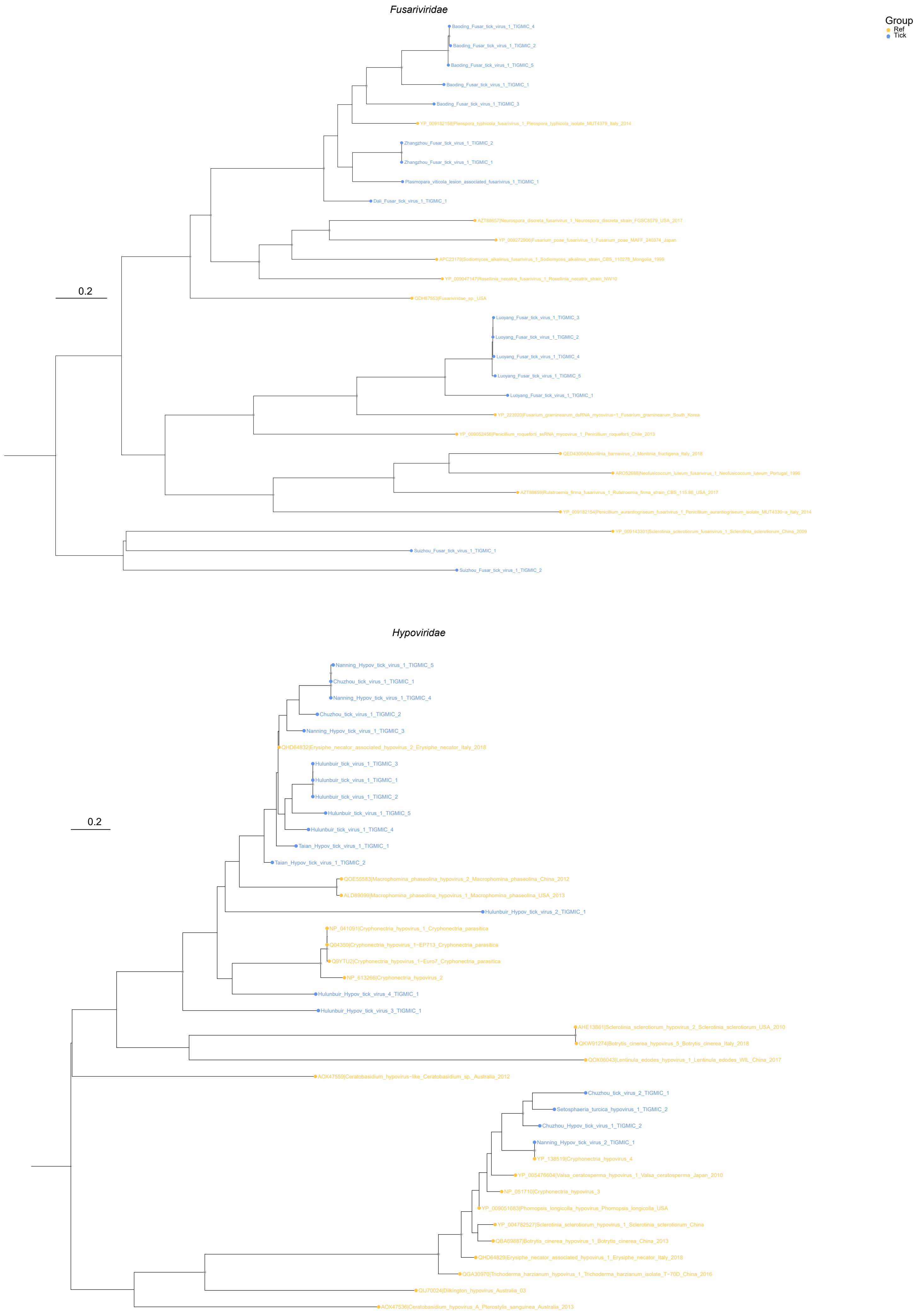
Fusariviridae
Group
Ref
Tick
0.2
Baoding_Fusar_tick_virus_1_TIGMIC_4
Baoding_Fusar_tick_virus_1_TIGMIC_2
Baoding_Fusar_tick_virus_1_TIGMIC_5
Baoding_Fusar_tick_virus_1_TIGMIC_1
Baoding_Fusar_tick_virus_1_TIGMIC_3
YP_009182158|Pleospora_typhicola_fusarivirus_1_Pleospora_typhicola_isolate_MUT4379_Italy_2014
Zhangzhou_Fusar_tick_virus_1_TIGMIC_2
Zhangzhou_Fusar_tick_virus_1_TIGMIC_1
Plasmopara_viticola_lesion_associated_fusarivirus_1_TIGMIC_1
Dali_Fusar_tick_virus_1_TIGMIC_1
AZT88657|Neurospora_discreta_fusarivirus_1_Neurospora_discreta_strain_FGSC8579_USA_2017
YP_009272906|Fusarium_poae_fusarivirus_1_Fusarium_poae_MAFF_240374_Japan
APC23179|Sodiomyces_alkalinus_fusarivirus_1_Sodiomyces_alkalinus_strain_CBS_110278_Mongolia_1999
YP_009047147|Rosellinia_necatrix_fusarivirus_1_Rosellinia_necatrix_strain_NW10
QDH87553|Fusariviridae_sp._USA
Luoyang_Fusar_tick_virus_1_TIGMIC_3
Luoyang_Fusar_tick_virus_1_TIGMIC_2
Luoyang_Fusar_tick_virus_1_TIGMIC_4
Luoyang_Fusar_tick_virus_1_TIGMIC_5
Luoyang_Fusar_tick_virus_1_TIGMIC_1
YP_223920|Fusarium_graminearum_dsRNA_mycovirus−1_Fusarium_graminearum_South_Korea
YP_009052456|Penicillium_roqueforti_ssRNA_mycovirus_1_Penicillium_roqueforti_Chile_2013
QED43004|Monilinia_barnavirus_J_Monilinia_fructigena_Italy_2018
ARO52688|Neofusicoccum_luteum_fusarivirus_1_Neofusicoccum_luteum_Portugal_1996
AZT88659|Rutstroemia_firma_fusarivirus_1_Rutstroemia_firma_strain_CBS_115.86_USA_2017
YP_009182154|Penicillium_aurantiogriseum_fusarivirus_1_Penicillium_aurantiogriseum_isolate_MUT4330−a_Italy_2014
YP_009143301|Sclerotinia_sclerotiorum_fusarivirus_1_Sclerotinia_sclerotiorum_China_2009
Suizhou_Fusar_tick_virus_1_TIGMIC_1
Suizhou_Fusar_tick_virus_1_TIGMIC_2
Hypoviridae
0.2
Nanning_Hypov_tick_virus_1_TIGMIC_5
Chuzhou_tick_virus_1_TIGMIC_1
Nanning_Hypov_tick_virus_1_TIGMIC_4
Chuzhou_tick_virus_1_TIGMIC_2
Nanning_Hypov_tick_virus_1_TIGMIC_3
QHD64832|Erysiphe_necator_associated_hypovirus_2_Erysiphe_necator_Italy_2018
Hulunbuir_tick_virus_1_TIGMIC_3
Hulunbuir_tick_virus_1_TIGMIC_1
Hulunbuir_tick_virus_1_TIGMIC_2
Hulunbuir_tick_virus_1_TIGMIC_5
Hulunbuir_tick_virus_1_TIGMIC_4
Taian_Hypov_tick_virus_1_TIGMIC_1
Taian_Hypov_tick_virus_1_TIGMIC_2
QOE55583|Macrophomina_phaseolina_hypovirus_2_Macrophomina_phaseolina_China_2012
ALD89099|Macrophomina_phaseolina_hypovirus_1_Macrophomina_phaseolina_USA_2013
Hulunbuir_Hypov_tick_virus_2_TIGMIC_1
NP_041091|Cryphonectria_hypovirus_1_Cryphonectria_parasitica
Q04350|Cryphonectria_hypovirus_1−EP713_Cryphonectria_parasitica
Q9YTU2|Cryphonectria_hypovirus_1−Euro7_Cryphonectria_parasitica
NP_613266|Cryphonectria_hypovirus_2
Hulunbuir_Hypov_tick_virus_4_TIGMIC_1
Hulunbuir_Hypov_tick_virus_3_TIGMIC_1
AHE13861|Sclerotinia_sclerotiorum_hypovirus_2_Sclerotinia_sclerotiorum_USA_2010
QKW91274|Botrytis_cinerea_hypovirus_5_Botrytis_cinerea_Italy_2018
QOX06043|Lentinula_edodes_hypovirus_1_Lentinula_edodes_WIL_China_2017
AOX47559|Ceratobasidium_hypovirus−like_Ceratobasidium_sp._Australia_2012
Chuzhou_tick_virus_2_TIGMIC_1
Setosphaeria_turcica_hypovirus_1_TIGMIC_2
Chuzhou_Hypov_tick_virus_1_TIGMIC_2
Nanning_Hypov_tick_virus_2_TIGMIC_1
YP_138519|Cryphonectria_hypovirus_4
YP_005476604|Valsa_ceratosperma_hypovirus_1_Valsa_ceratosperma_Japan_2010
NP_051710|Cryphonectria_hypovirus_3
YP_009051683|Phomopsis_longicolla_hypovirus_Phomopsis_longicolla_USA
YP_004782527|Sclerotinia_sclerotiorum_hypovirus_1_Sclerotinia_sclerotiorum_China
QBA69887|Botrytis_cinerea_hypovirus_1_Botrytis_cinerea_China_2013
QHD64829|Erysiphe_necator_associated_hypovirus_1_Erysiphe_necator_Italy_2018
QGA30970|Trichoderma_harzianum_hypovirus_1_Trichoderma_harzianum_isolate_T−70D_China_2016
QIJ70024|Dilkington_hypovirus_Australia_03
AOX47536|Ceratobasidium_hypovirus_A_Pterostylis_sanguinea_Australia_2013

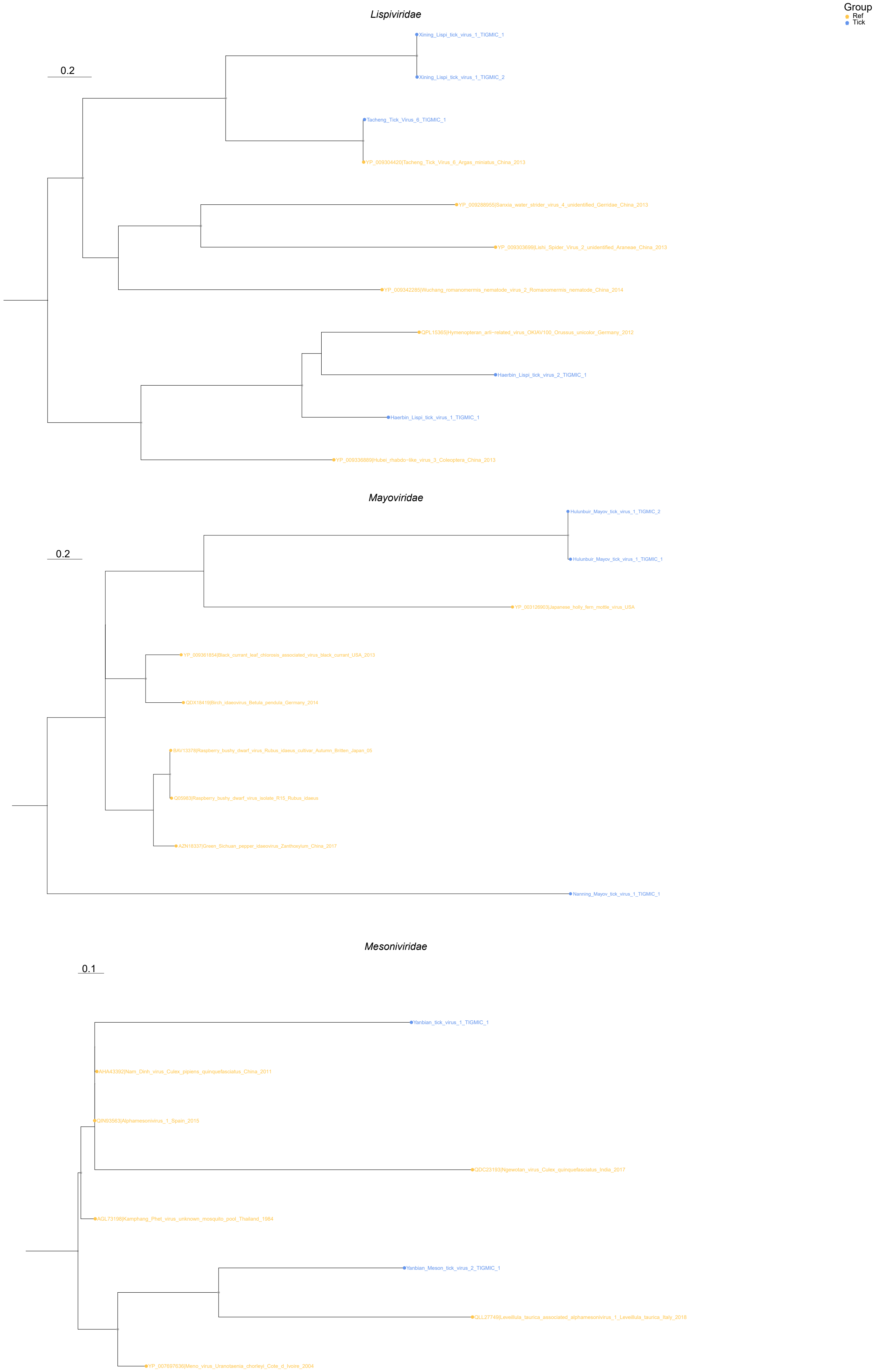
Lispiviridae
Group
Ref
Tick
0.2
Xining_Lispi_tick_virus_1_TIGMIC_1
Xining_Lispi_tick_virus_1_TIGMIC_2
Tacheng_Tick_Virus_6_TIGMIC_1
YP_009304420|Tacheng_Tick_Virus_6_Argas_miniatus_China_2013
YP_009288955|Sanxia_water_strider_virus_4_unidentified_Gerridae_China_2013
YP_009303698|Lishi_Spider_Virus_2_unidentified_Araneae_China_2013
YP_009342285|Wuchang_romanomermis_nematode_virus_2_Romanomermis_nematode_China_2014
QPL15365|Hymenopteran_arli−related_virus_OKIAV100_Orussus_unicolor_Germany_2012
Haerbin_Lispi_tick_virus_2_TIGMIC_1
Haerbin_Lispi_tick_virus_1_TIGMIC_1
YP_009336889|Hubei_rhabdo−like_virus_3_Coleoptera_China_2013
Mayoviridae
0.2
Hulunbuir_Mayov_tick_virus_1_TIGMIC_2
Hulunbuir_Mayov_tick_virus_1_TIGMIC_1
YP_003126903|Japanese_holly_fern_mottle_virus_USA
YP_009361854|Black_currant_leaf_chlorosis_associated_virus_black_currant_USA_2013
QDX18419|Birch_idaeovirus_Betula_pendula_Germany_2014
BAV13378|Raspberry_bushy_dwarf_virus_Rubus_idaeus_cultivar_Autumn_Britten_Japan_05
Q05983|Raspberry_bushy_dwarf_virus_isolate_R15_Rubus_idaeus
AZN18337|Green_Sichuan_pepper_idaeovirus_Zanthoxylum_China_2017
Nanning_Mayov_tick_virus_1_TIGMIC_1
Mesoniviridae
0.1
Yanbian_tick_virus_1_TIGMIC_1
AHA43392|Nam_Dinh_virus_Culex_pipiens_quinquefasciatus_China_2011
QIN93563|Alphamesonivirus_1_Spain_2015
QDC23193|Ngewotan_virus_Culex_quinquefasciatus_India_2017
AGL73198|Kamphang_Phet_virus_unknown_mosquito_pool_Thailand_1984
Yanbian_Meson_tick_virus_2_TIGMIC_1
QLL27749|Leveillula_taurica_associated_alphamesonivirus_1_Leveillula_taurica_Italy_2018
YP_007697636|Meno_virus_Uranotaenia_chorleyi_Cote_d_ivoire_2004

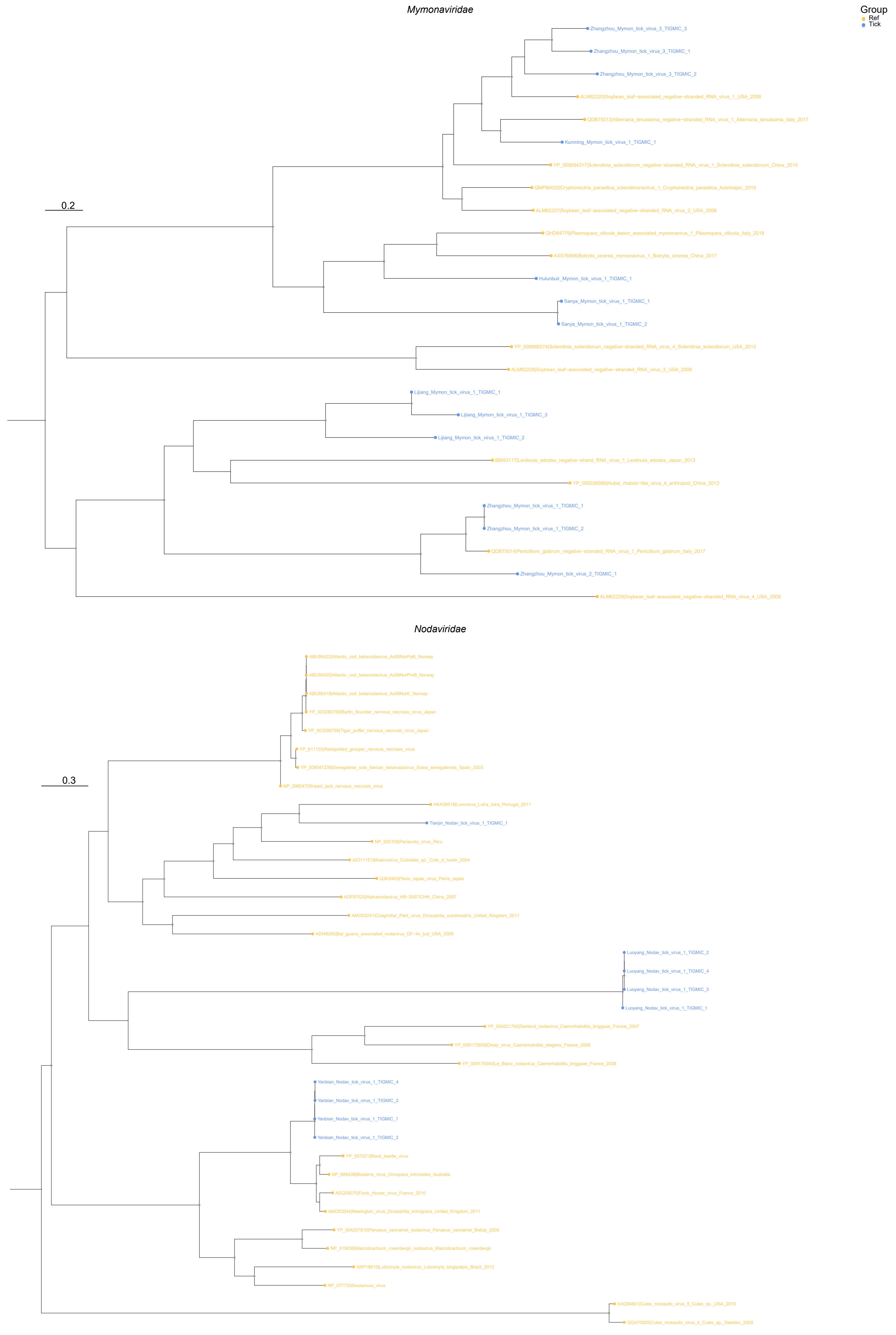
Mymonaviridae
Group
Ref
Tick
0.2
Zhangzhou_Mymon_tick_virus_3_TIGMIC_3
Zhangzhou_Mymon_tick_virus_3_TIGMIC_1
Zhangzhou_Mymon_tick_virus_3_TIGMIC_2
ALM62220|Soybean_leaf-associated_negative-stranded_RNA_virus_1_USA_2008
QDB75013|Alternaria_tenuissima_negative-stranded_RNA_virus_1_Alternaria_tenuissima_Italy_2017
Kunming_Mymon_tick_virus_1_TIGMIC_1
YP_009094317|Sclerotinia_sclerotiorum_negative-stranded_RNA_virus_1_Sclerotinia_sclerotiorum_China_2010
QMP84020|Cryphonectria_parasitica_sclerotimonavirus_1_Cryphonectria_parasitica_Azerbaijan_2019
ALM62227|Soybean_leaf-associated_negative-stranded_RNA_virus_2_USA_2008
QHD64779|Plasmopara_viticola_lesion_associated_mymonavirus_1_Plasmopara_viticola_Italy_2018
AXS76906|Botrytis_cinerea_mymonavirus_1_Botrytis_cinerea_China_2017
Hulunbuir_Mymon_tick_virus_1_TIGMIC_1
Sanya_Mymon_tick_virus_1_TIGMIC_1
Sanya_Mymon_tick_virus_1_TIGMIC_2
YP_009666274|Sclerotinia_sclerotiorum_negative-stranded_RNA_virus_4_Sclerotinia_sclerotiorum_USA_2013
ALM62228|Soybean_leaf-associated_negative-stranded_RNA_virus_3_USA_2008
Lijiang_Mymon_tick_virus_1_TIGMIC_1
Lijiang_Mymon_tick_virus_1_TIGMIC_3
Lijiang_Mymon_tick_virus_1_TIGMIC_2
BBI93117|Lentinula_edodes_negative-strand_RNA_virus_1_Lentinula_edodes_Japan_2013
YP_009336595|Hubei_rhabdo-like_virus_4_arthropod_China_2013
Zhangzhou_Mymon_tick_virus_1_TIGMIC_1
Zhangzhou_Mymon_tick_virus_1_TIGMIC_2
QDB75014|Penicillium_glabrum_negative-stranded_RNA_virus_1_Penicillium_glabrum_Italy_2017
Zhangzhou_Mymon_tick_virus_2_TIGMIC_1
ALM62229|Soybean_leaf-associated_negative-stranded_RNA_virus_4_USA_2008
Nodaviridae
0.3
ABU95422|Atlantic_cod_betanodavirus_Ac06NorPpB_Norway
ABU95420|Atlantic_cod_betanodavirus_Ac06NorPmB_Norway
ABU95418|Atlantic_cod_betanodavirus_Ac06NorE_Norway
YP_003288756|Barfin_flounder_nervous_necrosis_virus_Japan
YP_003288759|Tiger_puffer_nervous_necrosis_virus_Japan
YP_611155|Redspotted_grouper_nervous_necrosis_virus
YP_009047239|Senegalese_sole_Iberian_betanodavirus_Solea_senegalensis_Spain_2003
NP_599247|Striped_jack_nervous_necrosis_virus
AKA58518|Lunovirus_Lutra_lutra_Portugal_2011
Tianjin_Nodav_tick_virus_1_TIGMIC_1
NP_620109|Pariacoto_virus_Peru
AIO11151|Mosinovirus_Culicidae_sp._Cote_d_Ivoire_2004
Q3KSM3|Pieris_rapae_virus_Pieris_rapae
ADF97523|Alphanodavirus_HB-2007/CHN_China_2007
AMO03241|Craigmillar_Park_virus_Drosophila_subsilvestris_United_Kingdom_2011
ADI48250|Bat_guano_associated_nodavirus_GF-4n_bat_USA_2009
Luoyang_Nodav_tick_virus_1_TIGMIC_2
Luoyang_Nodav_tick_virus_1_TIGMIC_4
Luoyang_Nodav_tick_virus_1_TIGMIC_3
Luoyang_Nodav_tick_virus_1_TIGMIC_1
YP_004221742|Santeuil_nodavirus_Caenorhabditis_briggsae_France_2007
YP_009173859|Orsay_virus_Caenorhabditis_elegans_France_2008
YP_009175040|Le_Blanc_nodavirus_Caenorhabditis_briggsae_France_2008
Yanbian_Nodav_tick_virus_1_TIGMIC_4
Yanbian_Nodav_tick_virus_1_TIGMIC_2
Yanbian_Nodav_tick_virus_1_TIGMIC_1
Yanbian_Nodav_tick_virus_1_TIGMIC_3
YP_057021|Black_beetle_virus
NP_689439|Boolarra_virus_Oncopera_intricoides_Australia
AEQ39075|Flock_House_virus_France_2010
AMO03244|Newington_virus_Drosophila_immigrans_United_Kingdom_2011
YP_004207810|Penaeus_vannamei_nodavirus_Penaeus_vannamei_Belize_2005
NP_919036|Macrobrachium_rosenbergii_nodavirus_Macrobrachium_rosenbergii
AKP18615|Lutzomyia_nodavirus_Lutzomyia_longipalpis_Brazil_2012
NP_077730|Nodamura_virus
AXQ04841|Culex_mosquito_virus_5_Culex_sp._USA_2016
QGA70925|Culex_mosquito_virus_4_Culex_sp._Sweden_2009

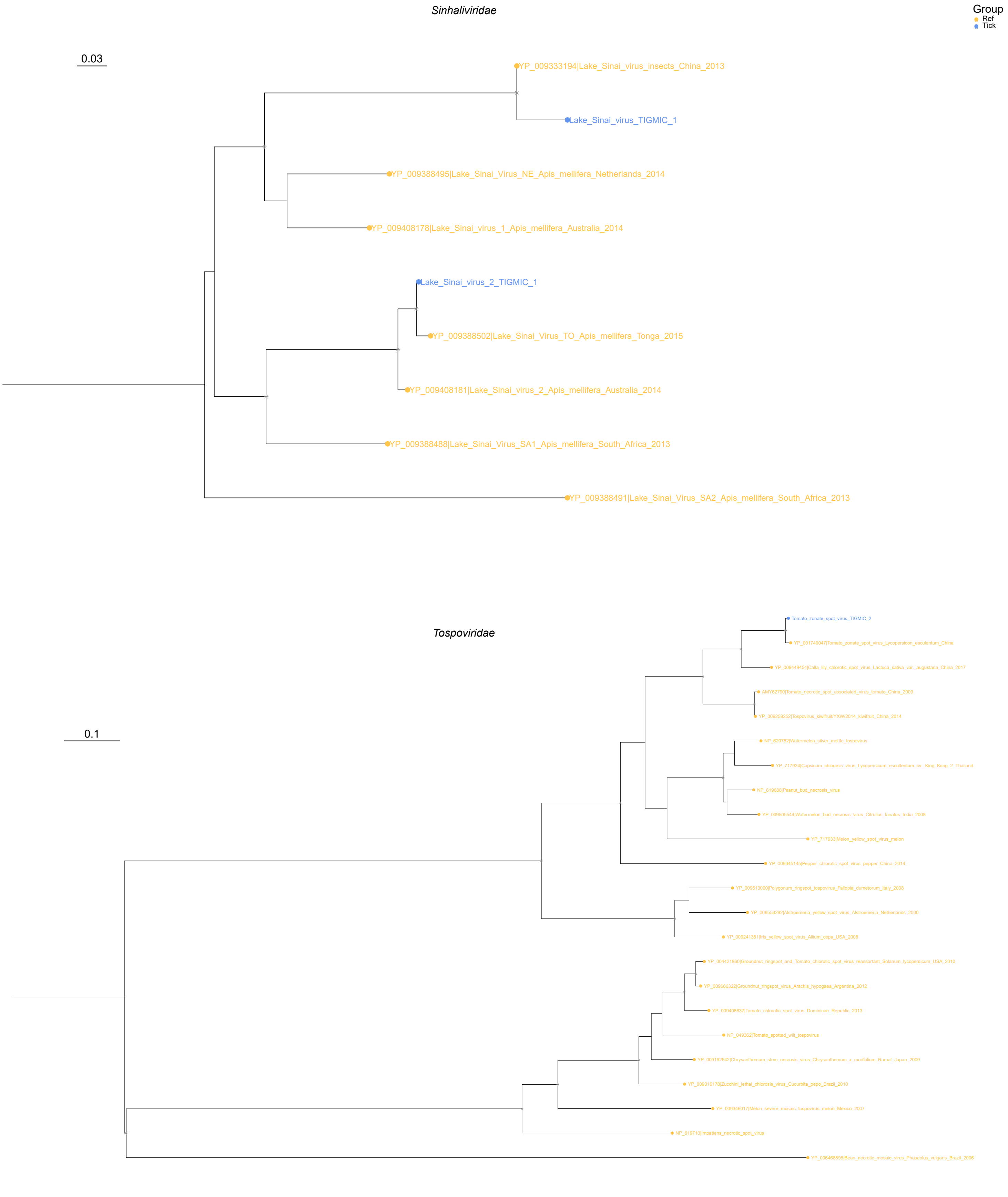

Sinhaliviridae
Group
Ref
Tick
0.03
YP_009333194|Lake_Sinai_virus_insects_China_2013
Lake_Sinai_virus_TIGMIC_1
YP_009388495|Lake_Sinai_Virus_NE_Apis_mellifera_Netherlands_2014
YP_009408178|Lake_Sinai_virus_1_Apis_mellifera_Australia_2014
Lake_Sinai_virus_2_TIGMIC_1
YP_009388502|Lake_Sinai_Virus_TO_Apis_mellifera_Tonga_2015
YP_009408181|Lake_Sinai_virus_2_Apis_mellifera_Australia_2014
YP_009388488|Lake_Sinai_Virus_SA1_Apis_mellifera_South_Africa_2013
YP_009388491|Lake_Sinai_Virus_SA2_Apis_mellifera_South_Africa_2013
Tospoviridae
0.1
Tomato_zonate_spot_virus_TIGMIC_2
YP_001740047|Tomato_zonate_spot_virus_Lycopersicon_esculentum_China
YP_009449454|Calla_lily_chlorotic_spot_virus_Lactuca_sativa_var._augustana_China_2017
AMY62790|Tomato_necrotic_spot_associated_virus_tomato_China_2009
YP_009259252|Tospovirus_kiwifruit/YXW/2014_kiwifruit_China_2014
NP_620752|Watermelon_silver_mottle_tospovirus
YP_717924|Capsicum_chlorosis_virus_Lycopersicum_escultentum_cv._King_Kong_2_Thailand
NP_619688|Peanut_bud_necrosis_virus
YP_009505544|Watermelon_bud_necrosis_virus_Citrullus_lanatus_India_2008
YP_717933|Melon_yellow_spot_virus_melon
YP_009345145|Pepper_chlorotic_spot_virus_pepper_China_2014
YP_009513000|Polygonum_ringspot_tospovirus_Fallopia_dumetorum_Italy_2008
YP_009553292|Alstroemeria_yellow_spot_virus_Alstroemeria_Netherlands_2000
YP_009241381|Iris_yellow_spot_virus_Allium_cepa_USA_2008
YP_004421860|Groundnut_ringspot_and_Tomato_chlorotic_spot_virus_reassortant_Solanum_lycopersicum_USA_2010
YP_009666322|Groundnut_ringspot_virus_Arachis_hypogaea_Argentina_2012
YP_009408637|Tomato_chlorotic_spot_virus_Dominican_Republic_2013
NP_049362|Tomato_spotted_wilt_tospovirus
YP_009162642|Chrysanthemum_stem_necrosis_virus_Chrysanthemum_x_morifolium_Ramat_Japan_2009
YP_009316178|Zucchini_lethal_chlorosis_virus_Cucurbita_pepo_Brazil_2010
YP_009346017|Melon_severe_mosaic_tospovirus_melon_Mexico_2007
NP_619710|Impatiens_necrotic_spot_virus
YP_006468898|Bean_necrotic_mosaic_virus_Phaseolus_vulgaris_Brazil_2006

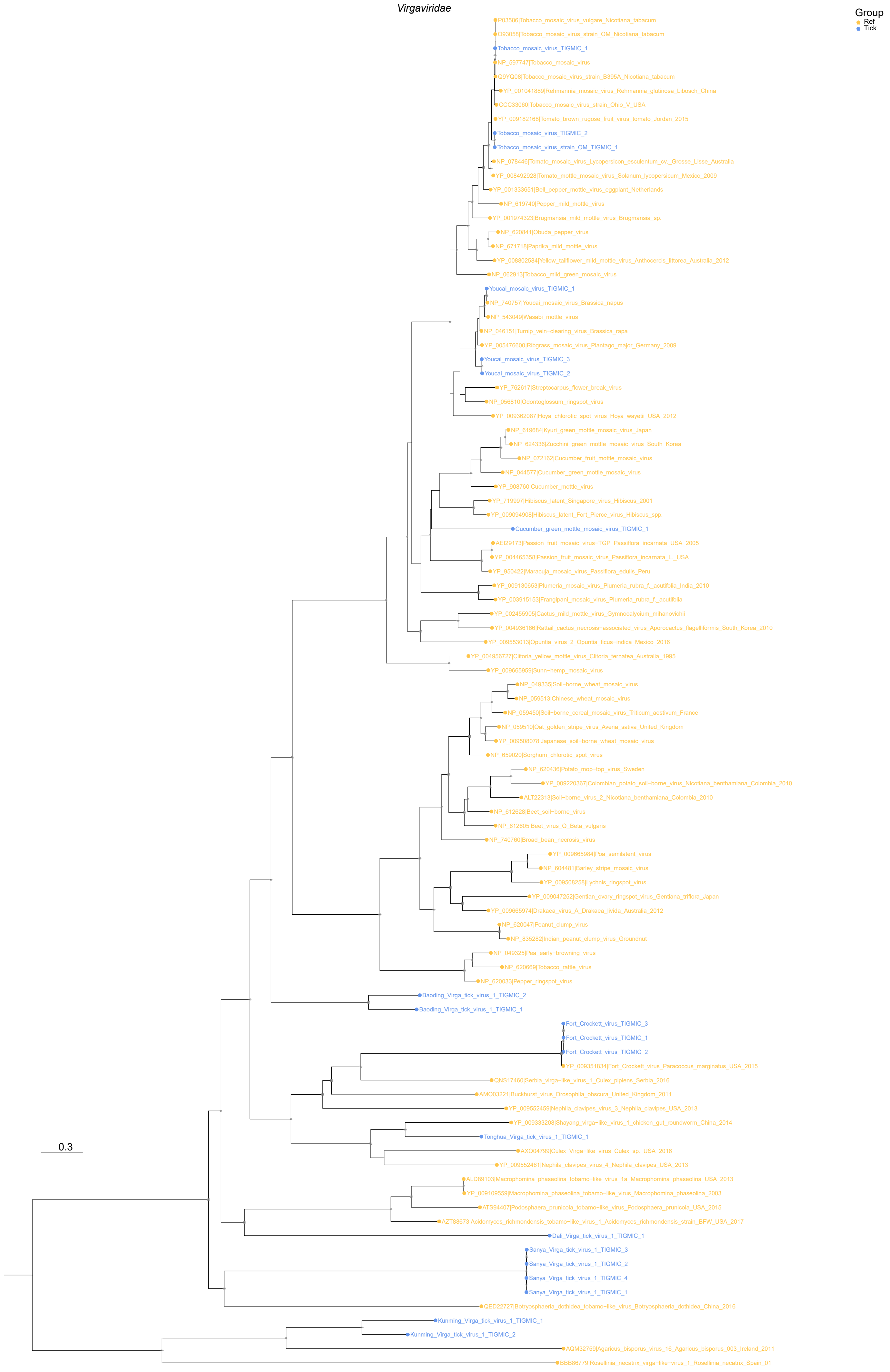
Virgaviridae
Group
Ref
Tick
P03586|Tobacco_mosaic_virus_vulgare_Nicotiana_tabacum
O93058|Tobacco_mosaic_virus_strain_OM_Nicotiana_tabacum
Tobacco_mosaic_virus_TIGMIC_1
NP_597747|Tobacco_mosaic_virus
Q9YQ08|Tobacco_mosaic_virus_strain_B395A_Nicotiana_tabacum
YP_001041889|Rehmannia_mosaic_virus_Rehmannia_glutinosa_Libosch_China
CCC33060|Tobacco_mosaic_virus_strain_Ohio_V_USA
YP_009182168|Tomato_brown_rugose_fruit_virus_tomato_Jordan_2015
Tobacco_mosaic_virus_TIGMIC_2
Tobacco_mosaic_virus_strain_OM_TIGMIC_1
NP_078446|Tomato_mosaic_virus_Lycopersicon_esculentum_cv._Grosse_Lisse_Australia
YP_008492928|Tomato_mottle_mosaic_virus_Solanum_lycopersicum_Mexico_2009
YP_001333651|Bell_pepper_mottle_virus_eggplant_Netherlands
NP_619740|Pepper_mild_mottle_virus
YP_001974323|Brugmansia_mild_mottle_virus_Brugmansia_sp.
NP_620841|Obuda_pepper_virus
NP_671718|Paprika_mild_mottle_virus
YP_008802584|Yellow_tailflower_mild_mottle_virus_Anthocercis_littorea_Australia_2012
NP_062913|Tobacco_mild_green_mosaic_virus
Youcai_mosaic_virus_TIGMIC_1
NP_740757|Youcai_mosaic_virus_Brassica_napus
NP_543049|Wasabi_mottle_virus
NP_046151|Turnip_vein-clearing_virus_Brassica_rapa
YP_005476600|Ribgrass_mosaic_virus_Plantago_major_Germany_2009
Youcai_mosaic_virus_TIGMIC_3
Youcai_mosaic_virus_TIGMIC_2
YP_762617|Streptocarpus_flower_break_virus
NP_056810|Odontoglossum_ringspot_virus
YP_009362087|Hoya_chlorotic_spot_virus_Hoya_wayetii_USA_2012
NP_619684|Kyuri_green_mottle_mosaic_virus_Japan
NP_624336|Zucchini_green_mottle_mosaic_virus_South_Korea
NP_072162|Cucumber_fruit_mottle_mosaic_virus
NP_044577|Cucumber_green_mottle_mosaic_virus
YP_908760|Cucumber_mottle_virus
YP_719997|Hibiscus_latent_Singapore_virus_Hibiscus_2001
YP_009094908|Hibiscus_latent_Fort_Pierce_virus_Hibiscus_spp.
Cucumber_green_mottle_mosaic_virus_TIGMIC_1
AEI29173|Passion_fruit_mosaic_virus-TGP_Passiflora_incarnata_USA_2005
YP_004465358|Passion_fruit_mosaic_virus_Passiflora_incarnata_L._USA
YP_950422|Maracuja_mosaic_virus_Passiflora_edulis_Peru
YP_009130653|Plumeria_mosaic_virus_Plumeria_rubra_f._acutifolia_India_2010
YP_003915153|Frangipani_mosaic_virus_Plumeria_rubra_f._acutifolia
YP_002455905|Cactus_mild_mottle_virus_Gymnocalycium_mihanovichii
YP_004936166|Rattail_cactus_necrosis-associated_virus_Aporocactus_flagelliformis_South_Korea_2010
YP_009553013|Opuntia_virus_2_Opuntia_ficus-indica_Mexico_2016
YP_004956727|Clitoria_yellow_mottle_virus_Clitoria_ternatea_Australia_1995
YP_009665969|Sunn-hemp_mosaic_virus
NP_049335|Soil-borne_wheat_mosaic_virus
NP_059513|Chinese_wheat_mosaic_virus
NP_059450|Soil-borne_cereal_mosaic_virus_Triticum_aestivum_France
NP_059510|Oat_golden_stripe_virus_Avena_sativa_United_Kingdom
YP_009508078|Japanese_soil-borne_wheat_mosaic_virus
NP_659020|Sorghum_chlorotic_spot_virus
NP_620436|Potato_mop-top_virus_Sweden
YP_009220367|Colombian_potato_soil-borne_virus_Nicotiana_benthamiana_Colombia_2010
ALT22313|Soil-borne_virus_2_Nicotiana_benthamiana_Colombia_2010
NP_612628|Beet_soil-borne_virus
NP_612605|Beet_virus_Q_Beta_vulgaris
NP_740760|Broad_bean_necrosis_virus
YP_009665984|Poa_semilatent_virus
NP_604481|Barley_stripe_mosaic_virus
YP_009508258|Lychnis_ringspot_virus
YP_009047252|Gentian_ovary_ringspot_virus_Gentiana_triflora_Japan
YP_009665974|Drakaea_virus_A_Drakaea_livida_Australia_2012
NP_620047|Peanut_clump_virus
NP_835282|Indian_peanut_clump_virus_Groundnut
NP_049325|Pea_early-browning_virus
NP_620669|Tobacco_rattle_virus
NP_620033|Pepper_ringspot_virus
Baoding_Virga_tick_virus_1_TIGMIC_2
Baoding_Virga_tick_virus_1_TIGMIC_1
Fort_Crockett_virus_TIGMIC_3
Fort_Crockett_virus_TIGMIC_1
Fort_Crockett_virus_TIGMIC_2
YP_009351834|Fort_Crockett_virus_Paracoccus_marginatus_USA_2015
QNS17460|Serbia_virga-like_virus_1_Culex_pipiens_Serbia_2016
AMO03221|Buckhurst_virus_Drosophila_obscura_United_Kingdom_2011
YP_009552459|Nephila_clavipes_virus_3_Nephila_clavipes_USA_2013
YP_009333208|Shayang_virga-like_virus_1_chicken_gut_roundworm_China_2014
Tonghua_Virga_tick_virus_1_TIGMIC_1
AXQ04799|Culex_Virga-like_virus_Culex_sp._USA_2016
YP_009552461|Nephila_clavipes_virus_4_Nephila_clavipes_USA_2013
ALD89103|Macrophomina_phaseolina_tobamo-like_virus_1a_Macrophomina_phaseolina_USA_2013
YP_009109559|Macrophomina_phaseolina_tobamo-like_virus_Macrophomina_phaseolina_2003
ATS94407|Podosphaera_prunicola_tobamo-like_virus_Podosphaera_prunicola_USA_2015
AZT88673|Acidomyces_richmondensis_tobamo-like_virus_1_Acidomyces_richmondensis_strain_BFW_USA_2017
Dali_Virga_tick_virus_1_TIGMIC_1
Sanya_Virga_tick_virus_1_TIGMIC_3
Sanya_Virga_tick_virus_1_TIGMIC_2
Sanya_Virga_tick_virus_1_TIGMIC_4
Sanya_Virga_tick_virus_1_TIGMIC_1
QED22727|Botryosphaeria_dothidea_tobamo-like_virus_Botryosphaeria_dothidea_China_2016
Kunming_Virga_tick_virus_1_TIGMIC_1
Kunming_Virga_tick_virus_1_TIGMIC_2
AQM32759|Agaricus_bisporus_virus_16_Agaricus_bisporus_003_Ireland_2011
BBB86779|Rosellinia_necatrix_virga-like-virus_1_Rosellinia_necatrix_Spain_01
0.3

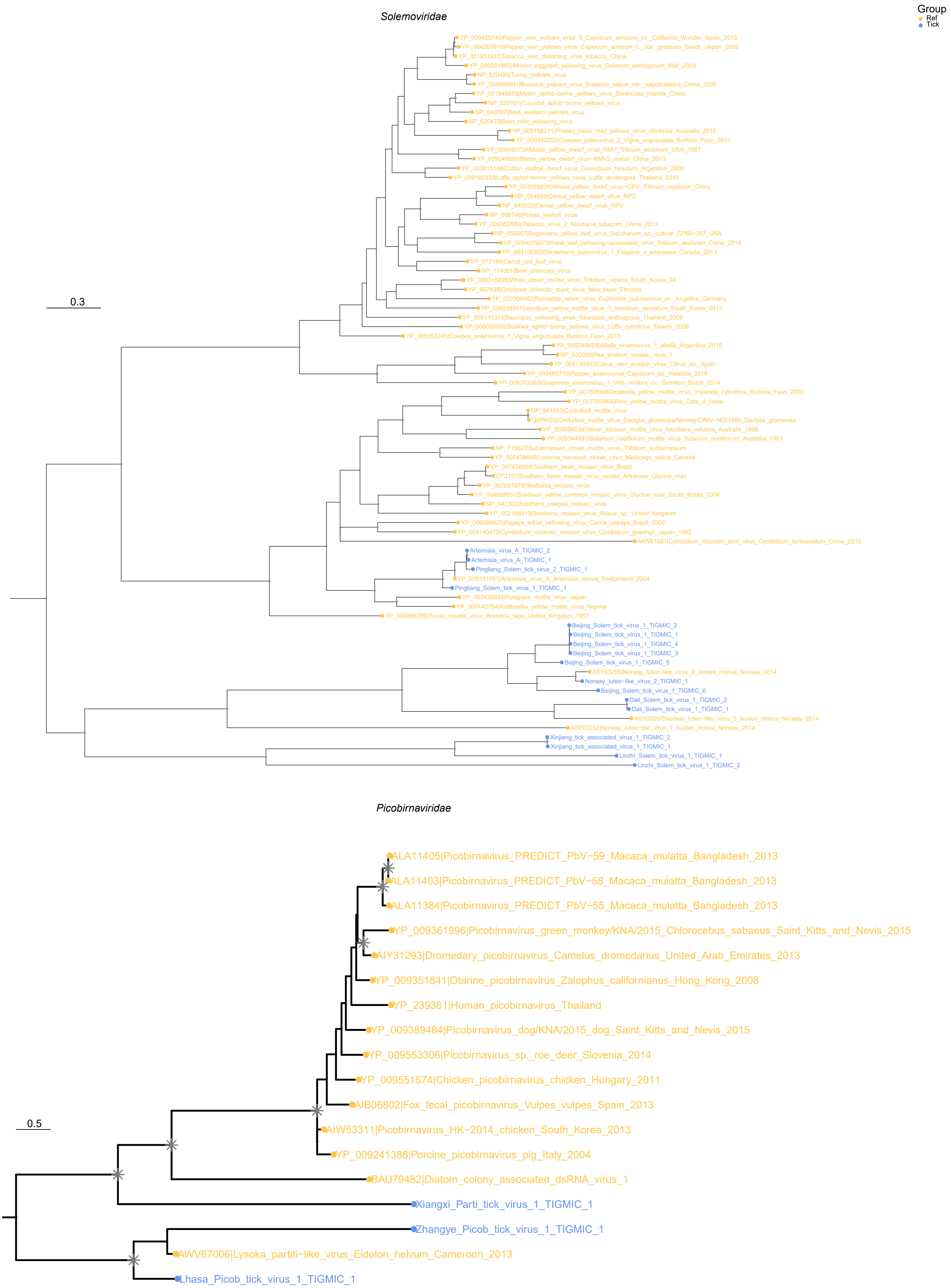
Solemoviridae
Group
Ref
Tick
0.3
YP_009455740|Pepper_vein_yellows_virus_5_Capsicum_annuum_cv._California_Wonder_Spain_2013
YP_004207916|Pepper_vein_yellows_virus_Capsicum_annuum_L._Var._grossum_Sendt._Japan_2009
YP_001931931|Tobacco_vein_distorting_virus_tobacco_China
YP_009351865|African_eggplant_yellowing_virus_Solanum_aethiopicum_Mali_2009
NP_620485|Turnip_yellows_virus
YP_004869991|Brassica_yellows_virus_Brassica_napus_var._napobrassica_China_2008
YP_001949870|Melon_aphid−borne_yellows_virus_Benincasa_hispida_China
NP_620101|Cucurbit_aphid−borne_yellows_virus
NP_840097|Beet_western_yellows_virus
NP_620479|Beet_mild_yellowing_virus
YP_009198211|Phasey_bean_mild_yellows_virus_chickpea_Australia_2013
YP_009352252|Cowpea_polerovirus_2_Vigna_unguiculata_Burkina_Faso_2013
YP_008083739|Maize_yellow_dwarf_virus_RMV_Triticum_aestivum_USA_1987
YP_009249569|Maize_yellow_dwarf_virus−RMV2_maize_China_2013
YP_003915148|Cotton_leafroll_dwarf_virus_Gossypium_hirsutum_Argentina_2006
YP_009162333|Luffa_aphid−borne_yellows_virus_Luffa_acutangula_Thailand_2010
YP_003029839|Wheat_yellow_dwarf_virus−GPV_Triticum_aestivum_China
NP_054685|Cereal_yellow_dwarf_virus_RPS
NP_840022|Cereal_yellow_dwarf_virus_RPV
NP_056748|Potato_leafroll_virus
YP_009352889|Tobacco_virus_2_Nicotiana_tabacum_China_2013
NP_050007|Sugarcane_yellow_leaf_virus_Saccharum_sp._cultivar_CP65−357_USA
YP_009407907|Wheat_leaf_yellowing−associated_virus_Triticum_aestivum_China_2016
YP_009100303|Strawberry_polerovirus_1_Fragaria_x_ananassa_Canada_2013
YP_077186|Carrot_red_leaf_virus
NP_114361|Beet_chlorosis_virus
YP_009315896|White_clover_mottle_virus_Trifolium_repens_South_Korea_04
YP_667838|Chickpea_chlorotic_stunt_virus_faba_bean_Ethiopia
YP_002308462|Poinsettia_latent_virus_Euphorbia_pulcherrima_cv._Angelika_Germany
YP_009238501|Ixeridium_yellow_mottle_virus_1_Ixeridium_dentatum_South_Korea_2013
YP_009111314|Sauropus_yellowing_virus_Sauropus_androgynus_Thailand_2009
YP_006666506|Suakwa_aphid−borne_yellows_virus_Luffa_cylindrica_Taiwan_2008
YP_009352245|Cowpea_polerovirus_1_Vigna_unguiculata_Burkina_Faso_2013
YP_009249823|Alfalfa_enamovirus_1_alfalfa_Argentina_2010
NP_620026|Pea_enation_mosaic_virus_1
YP_008130302|Citrus_vein_enation_virus_Citrus_sp._Spain
YP_009465710|Pepper_enamovirus_Capsicum_sp._Rwanda_2016
YP_009373263|Grapevine_enamovirus_1_Vitis_vinifera_cv._Semillon_Brazil_2014
YP_007506946|Imperata_yellow_mottle_virus_Imperata_cylindrica_Burkina_Faso_2000
YP_007500964|Rice_yellow_mottle_virus_Cote_d_Ivoire
NP_941957|Cocksfoot_mottle_virus
Q0PW25|Cocksfoot_mottle_virus_Dactylis_glomerata/Norway/CfMV−NO/1995_Dactylis_glomerata
YP_003896039|Velvet_tobacco_mottle_virus_Nicotiana_velutina_Australia_1986
YP_009344991|Solanum_nodiflorum_mottle_virus_Solanum_nodiflorum_Australia_1981
NP_715627|Subterranean_clover_mottle_virus_Trifolium_subterraneum
YP_007438849|Lucerne_transient_streak_virus_Medicago_sativa_Canada
YP_007438858|Southern_bean_mosaic_virus_Brazil
O72157|Southern_bean_mosaic_virus_isolate_Arkansas_Glycine_max
YP_007697678|Sesbania_mosaic_virus
YP_004869651|Soybean_yellow_common_mosaic_virus_Glycine_max_South_Korea_2004
NP_042302|Southern_cowpea_mosaic_virus
YP_002158813|Sowbane_mosaic_virus_Rubus_sp._United_Kingdom
YP_006589925|Papaya_lethal_yellowing_virus_Carica_papaya_Brazil_2009
YP_009140472|Cymbidium_chlorotic_mosaic_virus_Cymbidium_goeringii_Japan_1992
AKN81081|Cymbidium_chlorotic_spot_virus_Cymbidium_tortisepalum_China_2012
Artemisia_virus_A_TIGMIC_2
Artemisia_virus_A_TIGMIC_1
Pingliang_Solem_tick_virus_2_TIGMIC_1
YP_006331061|Artemisia_virus_A_Artemisia_annua_Switzerland_2004
Pingliang_Solem_tick_virus_1_TIGMIC_1
YP_007438853|Ryegrass_mottle_virus_Japan
YP_009142784|Rottboellia_yellow_mottle_virus_Nigeria
YP_008869286|Turnip_rosette_virus_Brassica_rapa_United_Kingdom_1957
Beijing_Solem_tick_virus_1_TIGMIC_2
Beijing_Solem_tick_virus_1_TIGMIC_1
Beijing_Solem_tick_virus_1_TIGMIC_4
Beijing_Solem_tick_virus_1_TIGMIC_3
Beijing_Solem_tick_virus_1_TIGMIC_5
ASY03255|Norway_luteo−like_virus_2_Ixodes_ricinus_Norway_2014
Norway_luteo−like_virus_2_TIGMIC_1
Beijing_Solem_tick_virus_1_TIGMIC_6
Dali_Solem_tick_virus_1_TIGMIC_2
Dali_Solem_tick_virus_1_TIGMIC_1
ASY03257|Norway_luteo−like_virus_3_Ixodes_ricinus_Norway_2014
ASY03252|Norway_luteo−like_virus_1_Ixodes_ricinus_Norway_2014
Xinjiang_tick_associated_virus_1_TIGMIC_2
Xinjiang_tick_associated_virus_1_TIGMIC_1
Linzhi_Solem_tick_virus_1_TIGMIC_1
Linzhi_Solem_tick_virus_1_TIGMIC_2
Picobirnaviridae
0.5
ALA11405|Picobirnavirus_PREDICT_PbV−59_Macaca_mulatta_Bangladesh_2013
ALA11403|Picobirnavirus_PREDICT_PbV−58_Macaca_mulatta_Bangladesh_2013
ALA11384|Picobirnavirus_PREDICT_PbV−55_Macaca_mulatta_Bangladesh_2013
YP_009361996|Picobirnavirus_green_monkey/KNA/2015_Chlorocebus_sabaeus_Saint_Kitts_and_Nevis_2015
AIY31293|Dromedary_picobirnavirus_Camelus_dromedarius_United_Arab_Emirates_2013
YP_009351841|Otarine_picobirnavirus_Zalophus_californianus_Hong_Kong_2008
YP_239361|Human_picobirnavirus_Thailand
YP_009389484|Picobirnavirus_dog/KNA/2015_dog_Saint_Kitts_and_Nevis_2015
YP_009553306|Picobirnavirus_sp._roe_deer_Slovenia_2014
YP_009551574|Chicken_picobirnavirus_chicken_Hungary_2011
AIB06802|Fox_fecal_picobirnavirus_Vulpes_vulpes_Spain_2013
AIW53311|Picobirnavirus_HK−2014_chicken_South_Korea_2013
YP_009241386|Porcine_picobirnavirus_pig_Italy_2004
BAU79482|Diatom_colony_associated_dsRNA_virus_1
Xiangxi_Parti_tick_virus_1_TIGMIC_1
Zhangye_Picob_tick_virus_1_TIGMIC_1
AWV67006|Lysoka_partiti−like_virus_Eidolon_helvum_Cameroon_2013
Lhasa_Picob_tick_virus_1_TIGMIC_1

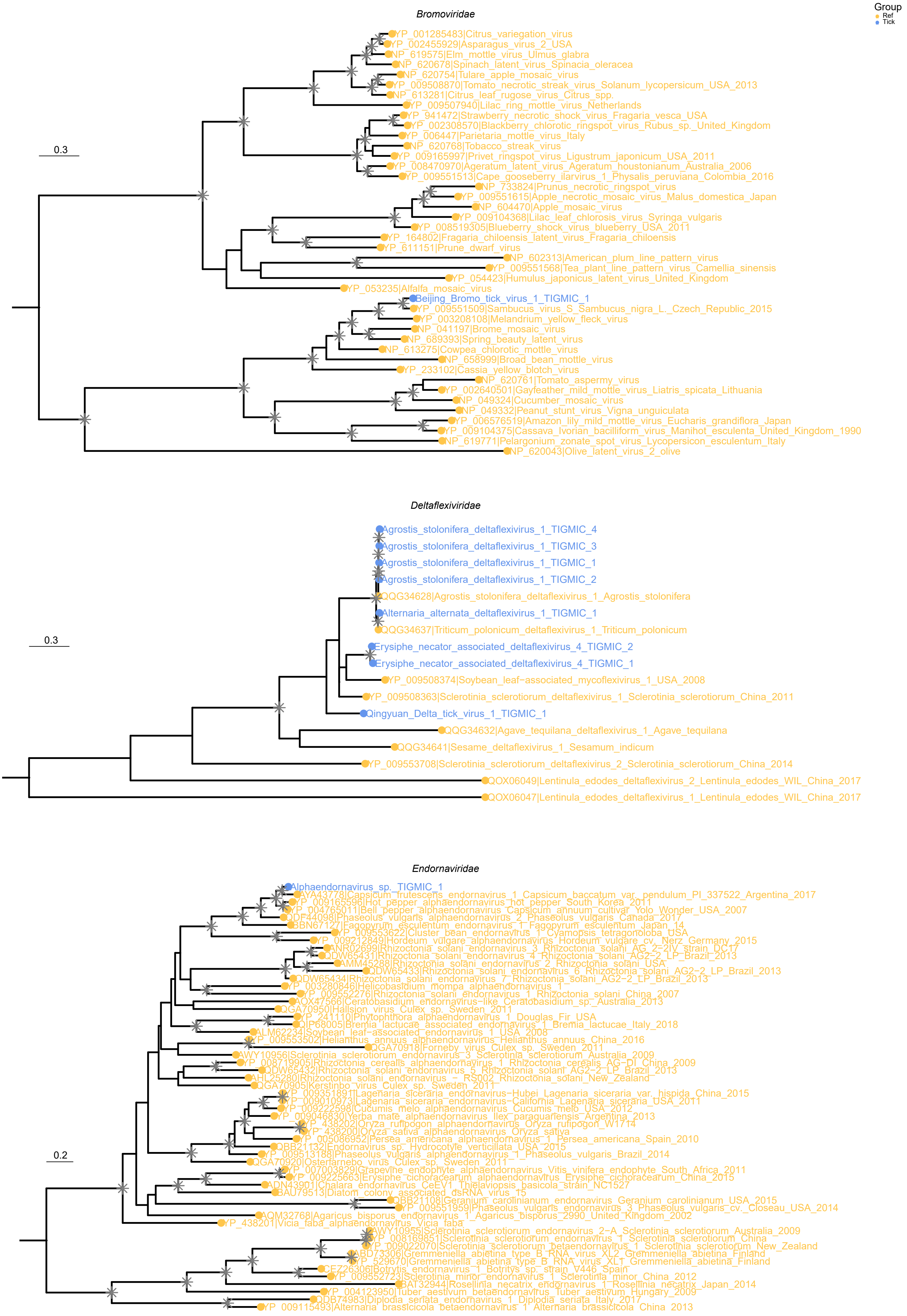

Group
Ref
Tick
Bromoviridae
0.3
YP_001285483|Citrus_variegation_virus
YP_002455929|Asparagus_virus_2_USA
NP_619575|Elm_mottle_virus_Ulmus_glabra
NP_620678|Spinach_latent_virus_Spinacia_oleracea
NP_620754|Tulare_apple_mosaic_virus
YP_009508870|Tomato_necrotic_streak_virus_Solanum_lycopersicum_USA_2013
NP_613281|Citrus_leaf_rugose_virus_Citrus_spp.
YP_009507940|Lilac_ring_mottle_virus_Netherlands
YP_941472|Strawberry_necrotic_shock_virus_Fragaria_vesca_USA
YP_002308570|Blackberry_chlorotic_ringspot_virus_Rubus_sp._United_Kingdom
YP_006447|Parietaria_mottle_virus_Italy
NP_620768|Tobacco_streak_virus
YP_009165997|Privet_ringspot_virus_Ligustrum_japonicum_USA_2011
YP_008470970|Ageratum_latent_virus_Ageratum_houstonianum_Australia_2006
YP_009551513|Cape_gooseberry_ilarvirus_1_Physalis_peruviana_Colombia_2016
NP_733824|Prunus_necrotic_ringspot_virus
YP_009551615|Apple_necrotic_mosaic_virus_Malus_domestica_Japan
NP_604470|Apple_mosaic_virus
YP_009104368|Lilac_leaf_chlorosis_virus_Syringa_vulgaris
YP_008519305|Blueberry_shock_virus_blueberry_USA_2011
YP_164802|Fragaria_chiloensis_latent_virus_Fragaria_chiloensis
YP_611151|Prune_dwarf_virus
NP_602313|American_plum_line_pattern_virus
YP_009551568|Tea_plant_line_pattern_virus_Camellia_sinensis
YP_054423|Humulus_japonicus_latent_virus_United_Kingdom
YP_053235|Alfalfa_mosaic_virus
Beijing_Bromo_tick_virus_1_TIGMIC_1
YP_009551509|Sambucus_virus_S_Sambucus_nigra_L._Czech_Republic_2015
YP_003208108|Melandrium_yellow_fleck_virus
NP_041197|Brome_mosaic_virus
NP_689393|Spring_beauty_latent_virus
NP_613275|Cowpea_chlorotic_mottle_virus
NP_658999|Broad_bean_mottle_virus
YP_233102|Cassia_yellow_blotch_virus
NP_620761|Tomato_aspermy_virus
YP_002640501|Gayfeather_mild_mottle_virus_Liatris_spicata_Lithuania
NP_049324|Cucumber_mosaic_virus
NP_049332|Peanut_stunt_virus_Vigna_unguiculata
YP_006576519|Amazon_lily_mild_mottle_virus_Eucharis_grandiflora_Japan
YP_009104375|Cassava_Ivorian_bacilliform_virus_Manihot_esculenta_United_Kingdom_1990
NP_619771|Pelargonium_zonate_spot_virus_Lycopersicon_esculentum_Italy
NP_620043|Olive_latent_virus_2_olive
Deltaflexiviridae
0.3
Agrostis_stolonifera_deltaflexivirus_1_TIGMIC_4
Agrostis_stolonifera_deltaflexivirus_1_TIGMIC_3
Agrostis_stolonifera_deltaflexivirus_1_TIGMIC_1
Agrostis_stolonifera_deltaflexivirus_1_TIGMIC_2
QQG34628|Agrostis_stolonifera_deltaflexivirus_1_Agrostis_stolonifera
Alternaria_alternata_deltaflexivirus_1_TIGMIC_1
QQG34637|Triticum_polonicum_deltaflexivirus_1_Triticum_polonicum
Erysiphe_necator_associated_deltaflexivirus_4_TIGMIC_2
Erysiphe_necator_associated_deltaflexivirus_4_TIGMIC_1
YP_009508374|Soybean_leaf-associated_mycoflexivirus_1_USA_2008
YP_009508363|Sclerotinia_sclerotiorum_deltaflexivirus_1_Sclerotinia_sclerotiorum_China_2011
Qingyuan_Delta_tick_virus_1_TIGMIC_1
QQG34632|Agave_tequilana_deltaflexivirus_1_Agave_tequilana
QQG34641|Sesame_deltaflexivirus_1_Sesamum_indicum
YP_009553708|Sclerotinia_sclerotiorum_deltaflexivirus_2_Sclerotinia_sclerotiorum_China_2014
QOX06049|Lentinula_edodes_deltaflexivirus_2_Lentinula_edodes_WIL_China_2017
QOX06047|Lentinula_edodes_deltaflexivirus_1_Lentinula_edodes_WIL_China_2017
Endornaviridae
0.2
Alphaendornavirus_sp._TIGMIC_1
AYA43778|Capsicum_frutescens_endornavirus_1_Capsicum_baccatum_var._pendulum_PI_337522_Argentina_2017
YP_009165596|Hot_pepper_alphaendornavirus_hot_pepper_South_Korea_2011
YP_004765011|Bell_pepper_alphaendornavirus_Capsicum_annuum_cultivar_Yolo_Wonder_USA_2007
QDF44098|Phaseolus_vulgaris_alphaendornavirus_2_Phaseolus_vulgaris_Canada_2017
BBN67127|Fagopyrum_esculentum_endornavirus_1_Fagopyrum_esculentum_Japan_14
YP_009553622|Cluster_bean_endornavirus_1_Cyamopsis_tetragonoloba_USA
YP_009212849|Hordeum_vulgare_alphaendornavirus_Hordeum_vulgare_cv._Nerz_Germany_2015
ANR02699|Rhizoctonia_solani_endornavirus_3_Rhizoctonia_solani_AG_2-2IV_strain_DC17
QDW65431|Rhizoctonia_solani_endornavirus_4_Rhizoctonia_solani_AG2-2_LP_Brazil_2013
AMM45288|Rhizoctonia_solani_endornavirus_2_Rhizoctonia_solani_USA
QDW65433|Rhizoctonia_solani_endornavirus_6_Rhizoctonia_solani_AG2-2_LP_Brazil_2013
QDW65434|Rhizoctonia_solani_endornavirus_7_Rhizoctonia_solani_AG2-2_LP_Brazil_2013
YP_003280846|Helicobasidium_mompa_alphaendornavirus_1
YP_009552276|Rhizoctonia_solani_endornavirus_1_Rhizoctonia_solani_China_2007
AOX47566|Ceratobasidium_endornavirus-like_Ceratobasidium_sp._Australia_2013
QGA70950|Hallsjon_virus_Culex_sp._Sweden_2011
YP_241110|Phytophthora_alphaendornavirus_1_Douglas_Fir_USA
QIP68005|Bremia_lactucae_associated_endornavirus_1_Bremia_lactucae_Italy_2018
ALM62234|Soybean_leaf-associated_endornavirus_1_USA_2008
YP_009553502|Helianthus_annuus_alphaendornavirus_Helianthus_annuus_China_2016
QGA70918|Forneby_virus_Culex_sp._Sweden_2011
AWY10956|Sclerotinia_sclerotiorum_endornavirus_3_Sclerotinia_sclerotiorum_Australia_2009
YP_008719905|Rhizoctonia_cerealis_alphaendornavirus_1_Rhizoctonia_cerealis_AG-DI_China_2009
QDW65432|Rhizoctonia_solani_endornavirus_5_Rhizoctonia_solani_AG2-2_LP_Brazil_2013
AHL25280|Rhizoctonia_solani_endornavirus_-_RS002_Rhizoctonia_solani_New_Zealand
QGA70905|Kerstinbo_virus_Culex_sp._Sweden_2011
YP_009351891|Lagenaria_siceraria_endornavirus-Hubei_Lagenaria_siceraria_var._hispida_China_2015
YP_009010973|Lagenaria_siceraria_endornavirus-California_Lagenaria_siceraria_USA_2011
YP_009222598|Cucumis_melo_alphaendornavirus_Cucumis_melo_USA_2012
YP_009046830|Yerba_mate_alphaendornavirus_Ilex_paraguariensis_Argentina_2013
YP_438202|Oryza_rufipogon_alphaendornavirus_Oryza_rufipogon_W1714
YP_438200|Oryza_sativa_alphaendornavirus_Oryza_sativa
YP_005086952|Persea_americana_alphaendornavirus_1_Persea_americana_Spain_2010
QBB21132|Endornavirus_sp._Hydrocotyle_verticillata_USA_2015
YP_009513188|Phaseolus_vulgaris_alphaendornavirus_1_Phaseolus_vulgaris_Brazil_2014
QGA70920|Osterfarnebo_virus_Culex_sp._Sweden_2011
YP_007003829|Grapevine_endophyte_alphaendornavirus_Vitis_vinifera_endophyte_South_Africa_2011
YP_009225663|Erysiphe_cichoracearum_alphaendornavirus_Erysiphe_cichoracearum_China_2015
ADN43901|Chalara_endornavirus_CeEV1_Thielaviopsis_basicola_strain_NC1527
BAU79513|Diatom_colony_associated_dsRNA_virus_15
QBB21108|Geranium_carolinianum_endornavirus_Geranium_carolinianum_USA_2015
YP_009551959|Phaseolus_vulgaris_endornavirus_3_Phaseolus_vulgaris_cv._Closeau_USA_2014
AQM32768|Agaricus_bisporus_endornavirus_1_Agaricus_bisporus_2990_United_Kingdom_2002
YP_438201|Vicia_faba_alphaendornavirus_Vicia_faba
AWY10955|Sclerotinia_sclerotiorum_endornavirus_2-A_Sclerotinia_sclerotiorum_Australia_2009
YP_008169851|Sclerotinia_sclerotiorum_endornavirus_1_Sclerotinia_sclerotiorum_China
YP_009022070|Sclerotinia_sclerotiorum_betaendornavirus_1_Sclerotinia_sclerotiorum_New_Zealand
ABD73306|Gremmeniella_abietina_type_B_RNA_virus_XL2_Gremmeniella_abietina_Finland
YP_529670|Gremmeniella_abietina_type_B_RNA_virus_XL1_Gremmeniella_abietina_Finland
CEZ26306|Botrytis_endornavirus_1_Botrytis_sp._strain_V446_Spain
YP_009552723|Sclerotinia_minor_endornavirus_1_Sclerotinia_minor_China_2012
BAT32944|Rosellinia_necatrix_endornavirus_1_Rosellinia_necatrix_Japan_2014
YP_004123950|Tuber_aestivum_betaendornavirus_Tuber_aestivum_Hungary_2009
QDB74983|Diplodia_seriata_endornavirus_1_Diplodia_seriata_Italy_2017
YP_009115493|Alternaria_brassicicola_betaendornavirus_1_Alternaria_brassicicola_China_2013

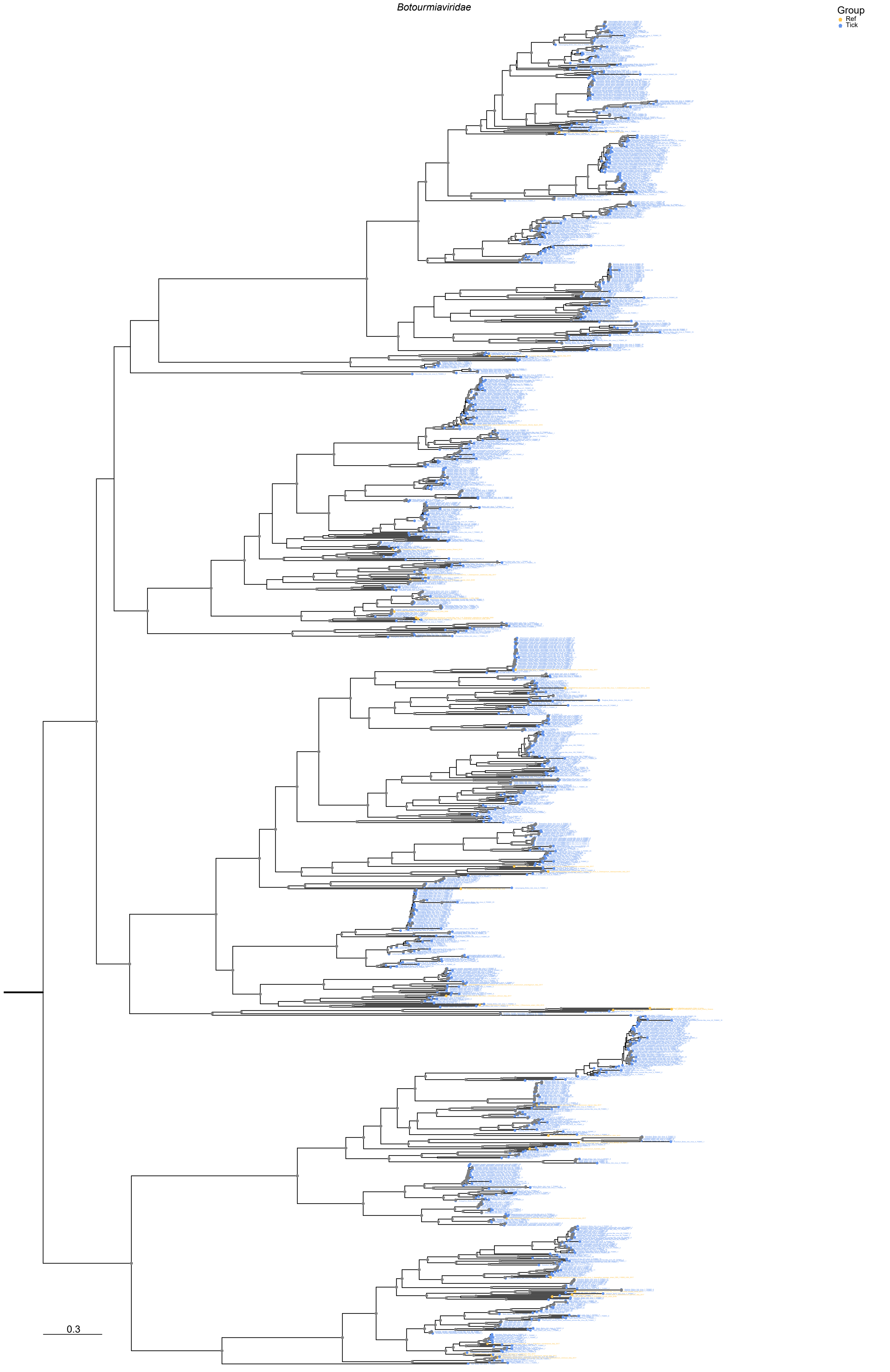

Botourmiaviridae
Group
Ref
Tick
0.3

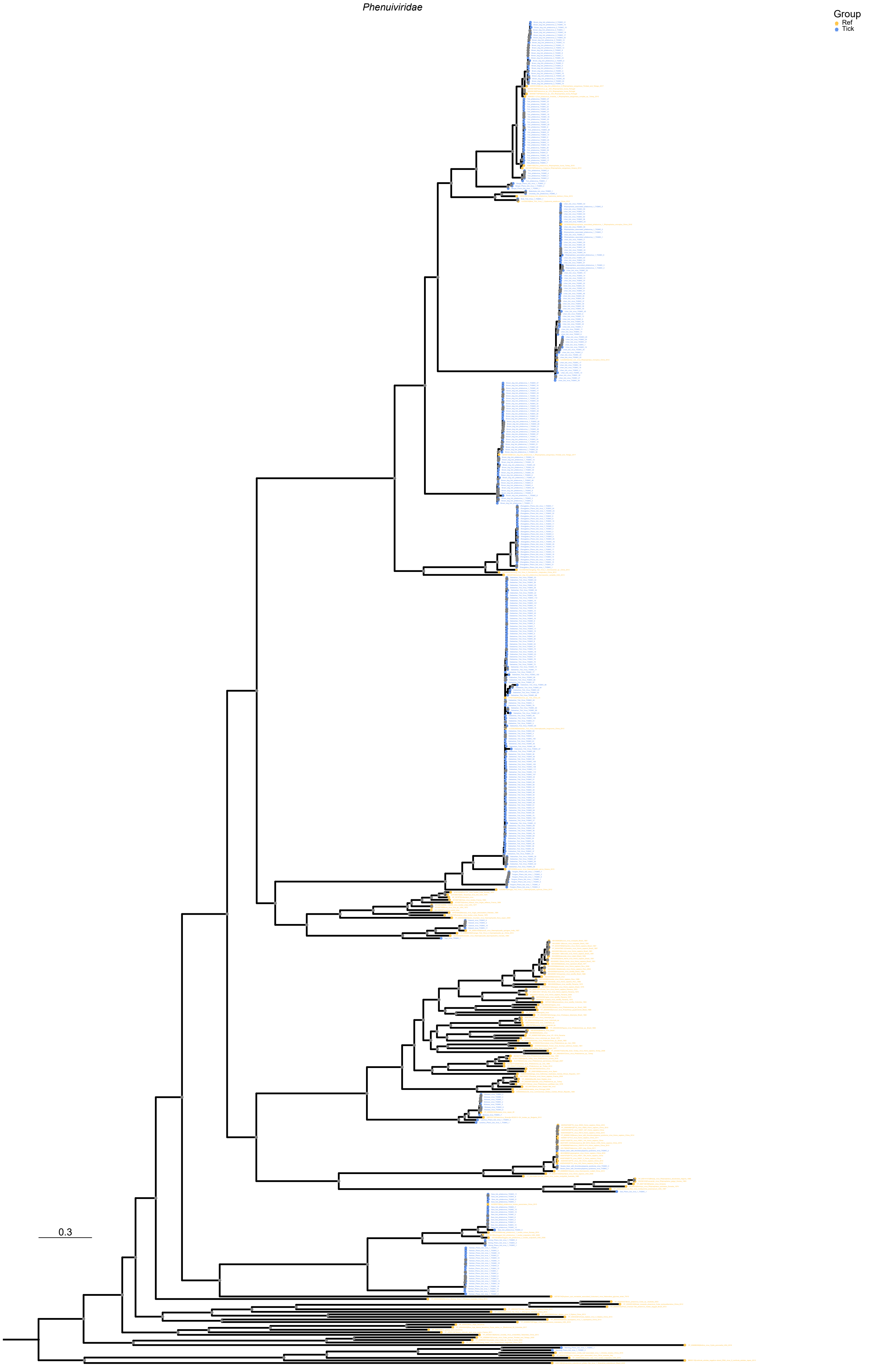
Phenuiviridae
Group
Ref
Tick
0.3

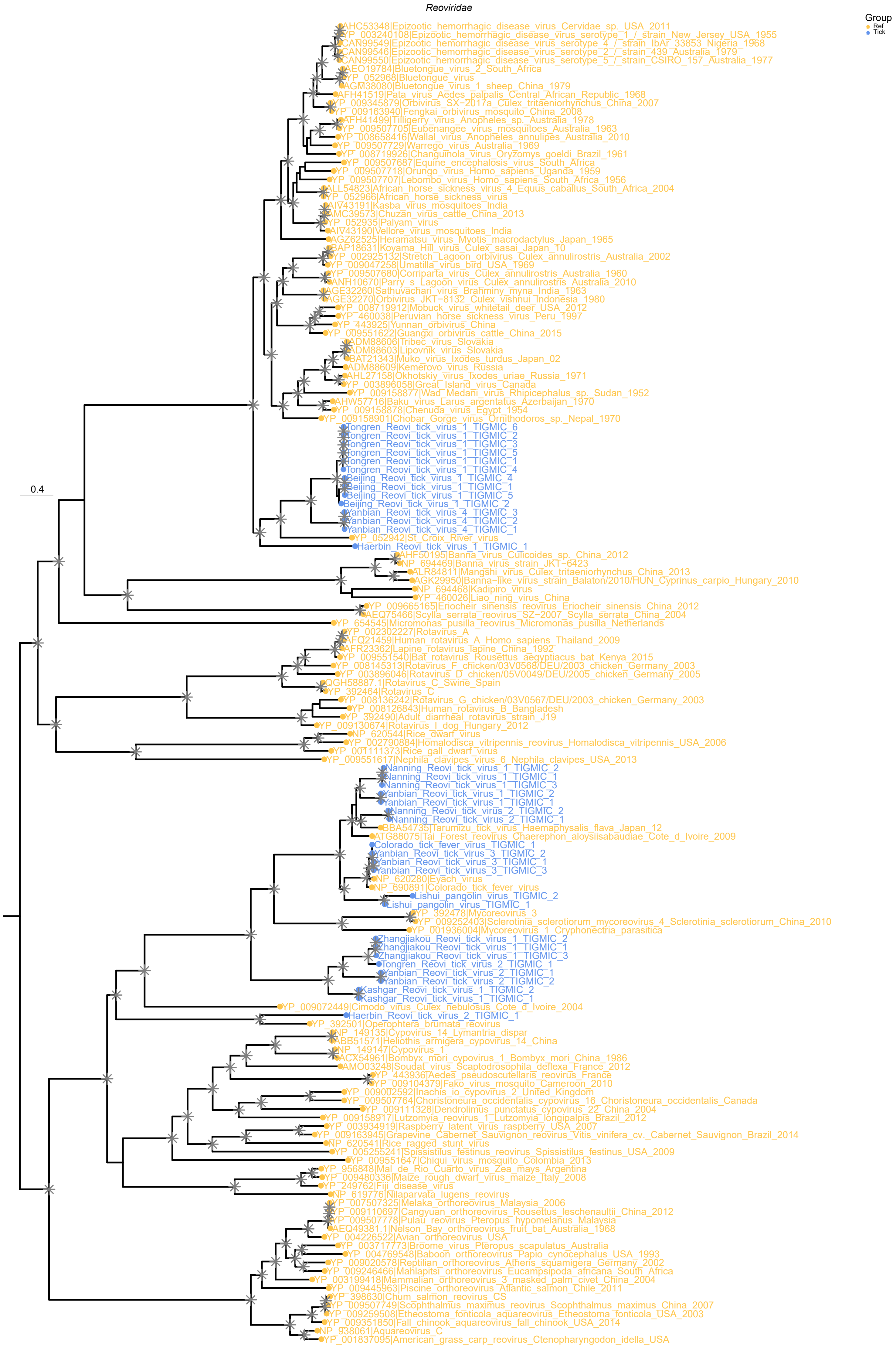
Reoviridae
Group
Ref
Tick
0.4
AHC53348|Epizootic_hemorrhagic_disease_virus_Cervidae_sp._USA_2011
YP_003240108|Epizootic_hemorrhagic_disease_virus_serotype_1_/_strain_New_Jersey_USA_1955
CAN99549|Epizootic_hemorrhagic_disease_virus_serotype_4_/_strain_IbAr_33853_Nigeria_1968
CAN99546|Epizootic_hemorrhagic_disease_virus_serotype_2_/_strain_439_Australia_1979
CAN99550|Epizootic_hemorrhagic_disease_virus_serotype_5_/_strain_CSIRO_157_Australia_1977
AEO19784|Bluetongue_virus_2_South_Africa
YP_052968|Bluetongue_virus
AGM38080|Bluetongue_virus_1_sheep_China_1979
AFH41519|Pata_virus_Aedes_palpalis_Central_African_Republic_1968
YP_009345879|Orbivirus_SX-2017a_Culex_tritaeniorhynchus_China_2007
YP_009163940|Fengkai_orbivirus_mosquito_China_2008
AFH41499|Tilligerry_virus_Anopheles_sp._Australia_1978
YP_009507705|Eubenangee_virus_mosquitoes_Australia_1963
YP_008658416|Wallal_virus_Anopheles_annulipes_Australia_2010
YP_009507729|Warrego_virus_Australia_1969
YP_008719926|Changuinola_virus_Oryzomys_goeldi_Brazil_1961
YP_009507687|Equine_encephalosis_virus_South_Africa
YP_009507718|Orungo_virus_Homo_sapiens_Uganda_1959
YP_009507707|Lebombo_virus_Homo_sapiens_South_Africa_1956
ALL54823|African_horse_sickness_virus_4_Equus_caballus_South_Africa_2004
YP_052966|African_horse_sickness_virus
AIV43191|Kasba_virus_mosquitoes_India
AMC39573|Chuzan_virus_cattle_China_2013
YP_052935|Palyam_virus
AIV43190|Vellore_virus_mosquitoes_India
AGZ62525|Heramatsu_virus_Myotis_macrodactylus_Japan_1965
BAP18631|Koyama_Hill_virus_Culex_sasai_Japan_10
YP_002925132|Stretch_Lagoon_orbivirus_Culex_annulirostris_Australia_2002
YP_009047258|Umatilla_virus_bird_USA_1969
YP_009507680|Corriparta_virus_Culex_annulirostris_Australia_1960
ANH10670|Parry_s_Lagoon_virus_Culex_annulirostris_Australia_2010
AGE32260|Sathuvachari_virus_Brahminy_myna_India_1963
AGE32270|Orbivirus_JKT-8132_Culex_vishnui_Indonesia_1980
YP_008719912|Mobuck_virus_whitetail_deer_USA_2012
YP_460038|Peruvian_horse_sickness_virus_Peru_1997
YP_443925|Yunnan_orbivirus_China
YP_009551622|Guangxi_orbivirus_cattle_China_2015
ADM88606|Tribec_virus_Slovakia
ADM88603|Lipovnik_virus_Slovakia
BAT21343|Muko_virus_Ixodes_turdus_Japan_02
ADM88609|Kemerovo_virus_Russia
AHL27158|Okhotskiy_virus_Ixodes_uriae_Russia_1971
YP_003896058|Great_Island_virus_Canada
YP_009158877|Wad_Medani_virus_Rhipicephalus_sp._Sudan_1952
AHW57716|Baku_virus_Larus_argentatus_Azerbaijan_1970
YP_009158878|Chenuda_virus_Egypt_1954
YP_009158901|Chobar_Gorge_virus_Ornithodoros_sp._Nepal_1970
Tongren_Reovi_tick_virus_1_TIGMIC_6
Tongren_Reovi_tick_virus_1_TIGMIC_2
Tongren_Reovi_tick_virus_1_TIGMIC_3
Tongren_Reovi_tick_virus_1_TIGMIC_5
Tongren_Reovi_tick_virus_1_TIGMIC_1
Tongren_Reovi_tick_virus_1_TIGMIC_4
Beijing_Reovi_tick_virus_1_TIGMIC_4
Beijing_Reovi_tick_virus_1_TIGMIC_1
Beijing_Reovi_tick_virus_1_TIGMIC_5
Beijing_Reovi_tick_virus_1_TIGMIC_2
Yanbian_Reovi_tick_virus_4_TIGMIC_3
Yanbian_Reovi_tick_virus_4_TIGMIC_2
Yanbian_Reovi_tick_virus_4_TIGMIC_1
YP_052942|St_Croix_River_virus
Haerbin_Reovi_tick_virus_1_TIGMIC_1
AHF50195|Banna_virus_Culicoides_sp._China_2012
NP_694469|Banna_virus_strain_JKT-6423
ALR84811|Mangshi_virus_Culex_tritaeniorhynchus_China_2013
AGK29950|Banna-like_virus_strain_Balaton/2010/HUN_Cyprinus_carpio_Hungary_2010
NP_694468|Kadipiro_virus
YP_460026|Liao_ning_virus_China
YP_009665165|Eriocheir_sinensis_reovirus_Eriocheir_sinensis_China_2012
AEQ75466|Scylla_serrata_reovirus_SZ-2007_Scylla_serrata_China_2004
YP_654545|Micromonas_pusilla_reovirus_Micromonas_pusilla_Netherlands
YP_002302227|Rotavirus_A
AFQ21459|Human_rotavirus_A_Homo_sapiens_Thailand_2009
AFR23362|Lapine_rotavirus_lapine_China_1992
YP_009551540|Bat_rotavirus_Rousettus_aegyptiacus_bat_Kenya_2015
YP_008145313|Rotavirus_F_chicken/03V0568/DEU/2003_chicken_Germany_2003
YP_003896046|Rotavirus_D_chicken/05V0049/DEU/2005_chicken_Germany_2005
QGH58887.1|Rotavirus_C_Swine_Spain
YP_392464|Rotavirus_C
YP_008136242|Rotavirus_G_chicken/03V0567/DEU/2003_chicken_Germany_2003
YP_008126843|Human_rotavirus_B_Bangladesh
YP_392490|Adult_diarrheal_rotavirus_strain_J19
YP_009130674|Rotavirus_I_dog_Hungary_2012
NP_620544|Rice_dwarf_virus
YP_002790884|Homalodisca_vitripennis_reovirus_Homalodisca_vitripennis_USA_2006
YP_001111373|Rice_gall_dwarf_virus
YP_009551617|Nephila_clavipes_virus_6_Nephila_clavipes_USA_2013
Nanning_Reovi_tick_virus_1_TIGMIC_2
Nanning_Reovi_tick_virus_1_TIGMIC_1
Nanning_Reovi_tick_virus_1_TIGMIC_3
Yanbian_Reovi_tick_virus_1_TIGMIC_2
Yanbian_Reovi_tick_virus_1_TIGMIC_1
Nanning_Reovi_tick_virus_2_TIGMIC_2
Nanning_Reovi_tick_virus_2_TIGMIC_1
BBA54735|Tarumizu_tick_virus_Haemaphysalis_flava_Japan_12
ATG88075|Tai_Forest_reovirus_Chaerephon_aloysiisabaudiae_Cote_d_Ivoire_2009
Colorado_tick_fever_virus_TIGMIC_1
Yanbian_Reovi_tick_virus_3_TIGMIC_2
Yanbian_Reovi_tick_virus_3_TIGMIC_1
Yanbian_Reovi_tick_virus_3_TIGMIC_3
NP_620280|Eyach_virus
NP_690891|Colorado_tick_fever_virus
Lishui_pangolin_virus_TIGMIC_2
Lishui_pangolin_virus_TIGMIC_1
YP_392478|Mycoreovirus_3
YP_009252403|Sclerotinia_sclerotiorum_mycoreovirus_4_Sclerotinia_sclerotiorum_China_2010
YP_001936004|Mycoreovirus_1_Cryphonectria_parasitica
Zhangjiakou_Reovi_tick_virus_1_TIGMIC_2
Zhangjiakou_Reovi_tick_virus_1_TIGMIC_1
Zhangjiakou_Reovi_tick_virus_1_TIGMIC_3
Tongren_Reovi_tick_virus_2_TIGMIC_1
Yanbian_Reovi_tick_virus_2_TIGMIC_1
Yanbian_Reovi_tick_virus_2_TIGMIC_2
Kashgar_Reovi_tick_virus_1_TIGMIC_2
Kashgar_Reovi_tick_virus_1_TIGMIC_1
YP_009072449|Cimodo_virus_Culex_nebulosus_Cote_d_Ivoire_2004
Haerbin_Reovi_tick_virus_2_TIGMIC_1
YP_392501|Operophtera_brumata_reovirus
NP_149135|Cypovirus_14_Lymantria_dispar
ABB51571|Heliothis_armigera_cypovirus_14_China
NP_149147|Cypovirus_1
ACX54961|Bombyx_mori_cypovirus_1_Bombyx_mori_China_1986
AMO03248|Soudat_virus_Scaptodrosophila_deflexa_France_2012
YP_443936|Aedes_pseudoscutellaris_reovirus_France
YP_009104379|Fako_virus_mosquito_Cameroon_2010
YP_009002592|Inachis_io_cypovirus_2_United_Kingdom
YP_009507764|Choristoneura_occidentalis_cypovirus_16_Choristoneura_occidentalis_Canada
YP_009111328|Dendrolimus_punctatus_cypovirus_22_China_2004
YP_009158917|Lutzomyia_reovirus_1_Lutzomyia_longipalpis_Brazil_2012
YP_003934919|Raspberry_latent_virus_raspberry_USA_2007
YP_009163945|Grapevine_Cabernet_Sauvignon_reovirus_Vitis_vinifera_cv._Cabernet_Sauvignon_Brazil_2014
NP_620541|Rice_ragged_stunt_virus
YP_005255241|Spissistilus_festinus_reovirus_Spissistilus_festinus_USA_2009
YP_009551647|Chiqui_virus_mosquito_Colombia_2013
YP_956848|Mal_de_Rio_Cuarto_virus_Zea_mays_Argentina
YP_009480336|Maize_rough_dwarf_virus_maize_Italy_2008
YP_249762|Fiji_disease_virus
NP_619776|Nilaparvata_lugens_reovirus
YP_007507325|Melaka_orthoreovirus_Malaysia_2006
YP_009110697|Cangyuan_orthoreovirus_Rousettus_leschenaultii_China_2012
YP_009507778|Pulau_reovirus_Pteropus_hypomelanus_Malaysia
AEQ49381.1|Nelson_Bay_orthoreovirus_fruit_bat_Australia_1968
YP_004226522|Avian_orthoreovirus_USA
YP_003717773|Broome_virus_Pteropus_scapulatus_Australia
YP_004769548|Baboon_orthoreovirus_Papio_cynocephalus_USA_1993
YP_009020578|Reptilian_orthoreovirus_Atheris_squamigera_Germany_2002
YP_009246466|Mahlapitsi_orthoreovirus_Eucampsipoda_africana_South_Africa
YP_003199418|Mammalian_orthoreovirus_3_masked_palm_civet_China_2004
YP_009445963|Piscine_orthoreovirus_Atlantic_salmon_Chile_2011
YP_398630|Chum_salmon_reovirus_CS
YP_009507749|Scophthalmus_maximus_reovirus_Scophthalmus_maximus_China_2007
YP_009259508|Etheostoma_fonticola_aquareovirus_Etheostoma_fonticola_USA_2003
YP_009351850|Fall_chinook_aquareovirus_fall_chinook_USA_2014
NP_938061|Aquareovirus_C
YP_001837095|American_grass_carp_reovirus_Ctenopharyngodon_idella_USA

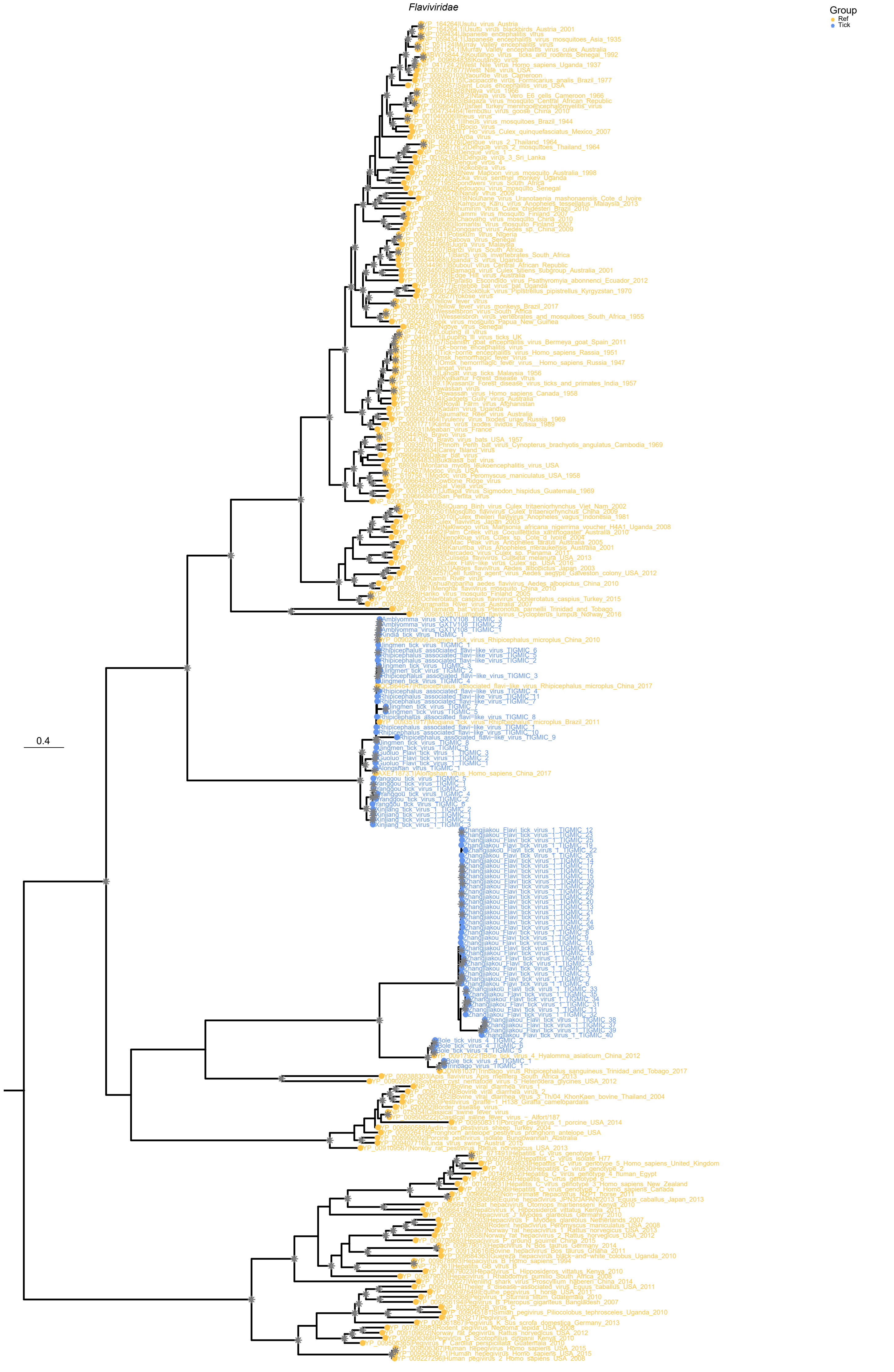
Flaviviridae
Group
Ref
Tick
0.4

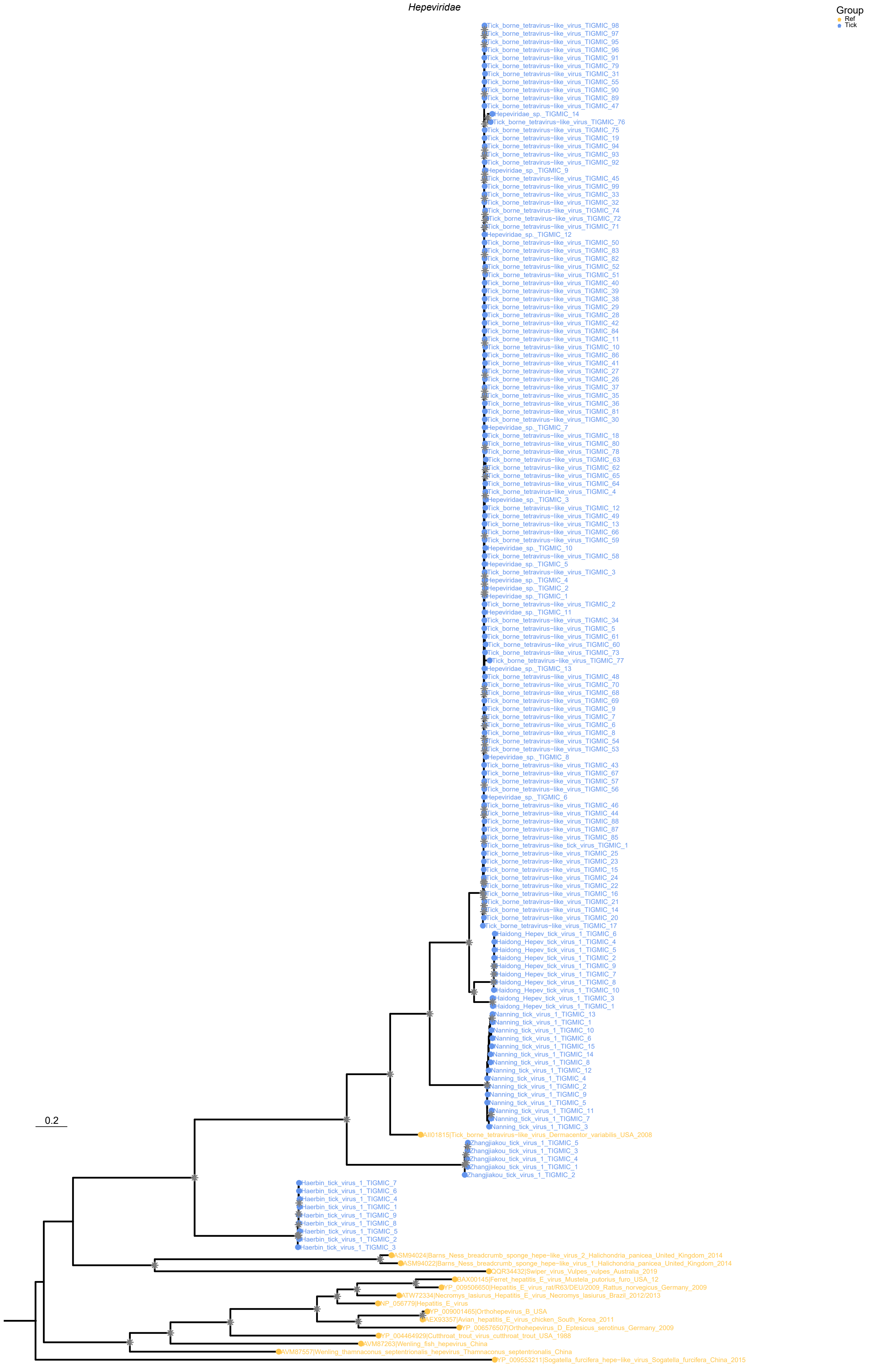

Hepeviridae
Group
Ref
Tick
0.2
Tick_borne_tetravirus−like_virus_TIGMIC_98
Tick_borne_tetravirus−like_virus_TIGMIC_97
Tick_borne_tetravirus−like_virus_TIGMIC_95
Tick_borne_tetravirus−like_virus_TIGMIC_96
Tick_borne_tetravirus−like_virus_TIGMIC_91
Tick_borne_tetravirus−like_virus_TIGMIC_79
Tick_borne_tetravirus−like_virus_TIGMIC_31
Tick_borne_tetravirus−like_virus_TIGMIC_55
Tick_borne_tetravirus−like_virus_TIGMIC_90
Tick_borne_tetravirus−like_virus_TIGMIC_89
Tick_borne_tetravirus−like_virus_TIGMIC_47
Hepeviridae_sp._TIGMIC_14
Tick_borne_tetravirus−like_virus_TIGMIC_76
Tick_borne_tetravirus−like_virus_TIGMIC_75
Tick_borne_tetravirus−like_virus_TIGMIC_19
Tick_borne_tetravirus−like_virus_TIGMIC_94
Tick_borne_tetravirus−like_virus_TIGMIC_93
Tick_borne_tetravirus−like_virus_TIGMIC_92
Hepeviridae_sp._TIGMIC_9
Tick_borne_tetravirus−like_virus_TIGMIC_45
Tick_borne_tetravirus−like_virus_TIGMIC_99
Tick_borne_tetravirus−like_virus_TIGMIC_33
Tick_borne_tetravirus−like_virus_TIGMIC_32
Tick_borne_tetravirus−like_virus_TIGMIC_74
Tick_borne_tetravirus−like_virus_TIGMIC_72
Tick_borne_tetravirus−like_virus_TIGMIC_71
Hepeviridae_sp._TIGMIC_12
Tick_borne_tetravirus−like_virus_TIGMIC_50
Tick_borne_tetravirus−like_virus_TIGMIC_83
Tick_borne_tetravirus−like_virus_TIGMIC_82
Tick_borne_tetravirus−like_virus_TIGMIC_52
Tick_borne_tetravirus−like_virus_TIGMIC_51
Tick_borne_tetravirus−like_virus_TIGMIC_40
Tick_borne_tetravirus−like_virus_TIGMIC_39
Tick_borne_tetravirus−like_virus_TIGMIC_38
Tick_borne_tetravirus−like_virus_TIGMIC_29
Tick_borne_tetravirus−like_virus_TIGMIC_28
Tick_borne_tetravirus−like_virus_TIGMIC_42
Tick_borne_tetravirus−like_virus_TIGMIC_84
Tick_borne_tetravirus−like_virus_TIGMIC_11
Tick_borne_tetravirus−like_virus_TIGMIC_10
Tick_borne_tetravirus−like_virus_TIGMIC_86
Tick_borne_tetravirus−like_virus_TIGMIC_41
Tick_borne_tetravirus−like_virus_TIGMIC_27
Tick_borne_tetravirus−like_virus_TIGMIC_26
Tick_borne_tetravirus−like_virus_TIGMIC_37
Tick_borne_tetravirus−like_virus_TIGMIC_35
Tick_borne_tetravirus−like_virus_TIGMIC_36
Tick_borne_tetravirus−like_virus_TIGMIC_81
Tick_borne_tetravirus−like_virus_TIGMIC_30
Hepeviridae_sp._TIGMIC_7
Tick_borne_tetravirus−like_virus_TIGMIC_18
Tick_borne_tetravirus−like_virus_TIGMIC_80
Tick_borne_tetravirus−like_virus_TIGMIC_78
Tick_borne_tetravirus−like_virus_TIGMIC_63
Tick_borne_tetravirus−like_virus_TIGMIC_62
Tick_borne_tetravirus−like_virus_TIGMIC_65
Tick_borne_tetravirus−like_virus_TIGMIC_64
Tick_borne_tetravirus−like_virus_TIGMIC_4
Hepeviridae_sp._TIGMIC_3
Tick_borne_tetravirus−like_virus_TIGMIC_12
Tick_borne_tetravirus−like_virus_TIGMIC_49
Tick_borne_tetravirus−like_virus_TIGMIC_13
Tick_borne_tetravirus−like_virus_TIGMIC_66
Tick_borne_tetravirus−like_virus_TIGMIC_59
Hepeviridae_sp._TIGMIC_10
Tick_borne_tetravirus−like_virus_TIGMIC_58
Hepeviridae_sp._TIGMIC_5
Tick_borne_tetravirus−like_virus_TIGMIC_3
Hepeviridae_sp._TIGMIC_4
Hepeviridae_sp._TIGMIC_2
Hepeviridae_sp._TIGMIC_1
Tick_borne_tetravirus−like_virus_TIGMIC_2
Hepeviridae_sp._TIGMIC_11
Tick_borne_tetravirus−like_virus_TIGMIC_34
Tick_borne_tetravirus−like_virus_TIGMIC_5
Tick_borne_tetravirus−like_virus_TIGMIC_61
Tick_borne_tetravirus−like_virus_TIGMIC_60
Tick_borne_tetravirus−like_virus_TIGMIC_73
Tick_borne_tetravirus−like_virus_TIGMIC_77
Hepeviridae_sp._TIGMIC_13
Tick_borne_tetravirus−like_virus_TIGMIC_48
Tick_borne_tetravirus−like_virus_TIGMIC_70
Tick_borne_tetravirus−like_virus_TIGMIC_68
Tick_borne_tetravirus−like_virus_TIGMIC_69
Tick_borne_tetravirus−like_virus_TIGMIC_9
Tick_borne_tetravirus−like_virus_TIGMIC_7
Tick_borne_tetravirus−like_virus_TIGMIC_6
Tick_borne_tetravirus−like_virus_TIGMIC_8
Tick_borne_tetravirus−like_virus_TIGMIC_54
Tick_borne_tetravirus−like_virus_TIGMIC_53
Hepeviridae_sp._TIGMIC_8
Tick_borne_tetravirus−like_virus_TIGMIC_43
Tick_borne_tetravirus−like_virus_TIGMIC_67
Tick_borne_tetravirus−like_virus_TIGMIC_57
Tick_borne_tetravirus−like_virus_TIGMIC_56
Hepeviridae_sp._TIGMIC_6
Tick_borne_tetravirus−like_virus_TIGMIC_46
Tick_borne_tetravirus−like_virus_TIGMIC_44
Tick_borne_tetravirus−like_virus_TIGMIC_88
Tick_borne_tetravirus−like_virus_TIGMIC_87
Tick_borne_tetravirus−like_virus_TIGMIC_85
Tick_borne_tetravirus−like_tick_virus_TIGMIC_1
Tick_borne_tetravirus−like_virus_TIGMIC_25
Tick_borne_tetravirus−like_virus_TIGMIC_23
Tick_borne_tetravirus−like_virus_TIGMIC_15
Tick_borne_tetravirus−like_virus_TIGMIC_24
Tick_borne_tetravirus−like_virus_TIGMIC_22
Tick_borne_tetravirus−like_virus_TIGMIC_16
Tick_borne_tetravirus−like_virus_TIGMIC_21
Tick_borne_tetravirus−like_virus_TIGMIC_14
Tick_borne_tetravirus−like_virus_TIGMIC_20
Tick_borne_tetravirus−like_virus_TIGMIC_17
Haidong_Hepev_tick_virus_1_TIGMIC_6
Haidong_Hepev_tick_virus_1_TIGMIC_4
Haidong_Hepev_tick_virus_1_TIGMIC_5
Haidong_Hepev_tick_virus_1_TIGMIC_2
Haidong_Hepev_tick_virus_1_TIGMIC_9
Haidong_Hepev_tick_virus_1_TIGMIC_7
Haidong_Hepev_tick_virus_1_TIGMIC_8
Haidong_Hepev_tick_virus_1_TIGMIC_10
Haidong_Hepev_tick_virus_1_TIGMIC_3
Haidong_Hepev_tick_virus_1_TIGMIC_1
Nanning_tick_virus_1_TIGMIC_13
Nanning_tick_virus_1_TIGMIC_1
Nanning_tick_virus_1_TIGMIC_10
Nanning_tick_virus_1_TIGMIC_6
Nanning_tick_virus_1_TIGMIC_15
Nanning_tick_virus_1_TIGMIC_14
Nanning_tick_virus_1_TIGMIC_8
Nanning_tick_virus_1_TIGMIC_12
Nanning_tick_virus_1_TIGMIC_4
Nanning_tick_virus_1_TIGMIC_2
Nanning_tick_virus_1_TIGMIC_9
Nanning_tick_virus_1_TIGMIC_5
Nanning_tick_virus_1_TIGMIC_11
Nanning_tick_virus_1_TIGMIC_7
Nanning_tick_virus_1_TIGMIC_3
AII01815|Tick_borne_tetravirus−like_virus_Dermacentor_variabilis_USA_2008
Zhangjiakou_tick_virus_1_TIGMIC_5
Zhangjiakou_tick_virus_1_TIGMIC_3
Zhangjiakou_tick_virus_1_TIGMIC_4
Zhangjiakou_tick_virus_1_TIGMIC_1
Zhangjiakou_tick_virus_1_TIGMIC_2
Haerbin_tick_virus_1_TIGMIC_7
Haerbin_tick_virus_1_TIGMIC_6
Haerbin_tick_virus_1_TIGMIC_4
Haerbin_tick_virus_1_TIGMIC_1
Haerbin_tick_virus_1_TIGMIC_9
Haerbin_tick_virus_1_TIGMIC_8
Haerbin_tick_virus_1_TIGMIC_5
Haerbin_tick_virus_1_TIGMIC_2
Haerbin_tick_virus_1_TIGMIC_3
ASM94024|Barns_Ness_breadcrumb_sponge_hepe−like_virus_2_Halichondria_panicea_United_Kingdom_2014
ASM94022|Barns_Ness_breadcrumb_sponge_hepe−like_virus_1_Halichondria_panicea_United_Kingdom_2014
QQR34432|Swiper_virus_Vulpes_vulpes_Australia_2019
BAX00145|Ferret_hepatitis_E_virus_Mustela_putorius_furo_USA_12
YP_009506650|Hepatitis_E_virus_rat/R63/DEU/2009_Rattus_norvegicus_Germany_2009
ATW72334|Necromys_lasiurus_Hepatitis_E_virus_Necromys_lasiurus_Brazil_2012/2013
NP_056779|Hepatitis_E_virus
YP_009001465|Orthohepevirus_B_USA
AEX93357|Avian_hepatitis_E_virus_chicken_South_Korea_2011
YP_008576507|Orthohepevirus_D_Eptesicus_serotinus_Germany_2009
YP_004464929|Cutthroat_trout_virus_cutthroat_trout_USA_1988
AVM87263|Wenling_fish_hepevirus_China
AVM87557|Wenling_thamnaconus_septentrionalis_hepevirus_Thamnaconus_septentrionalis_China
YP_009553211|Sogatella_furcifera_hepe−like_virus_Sogatella_furcifera_China_2015

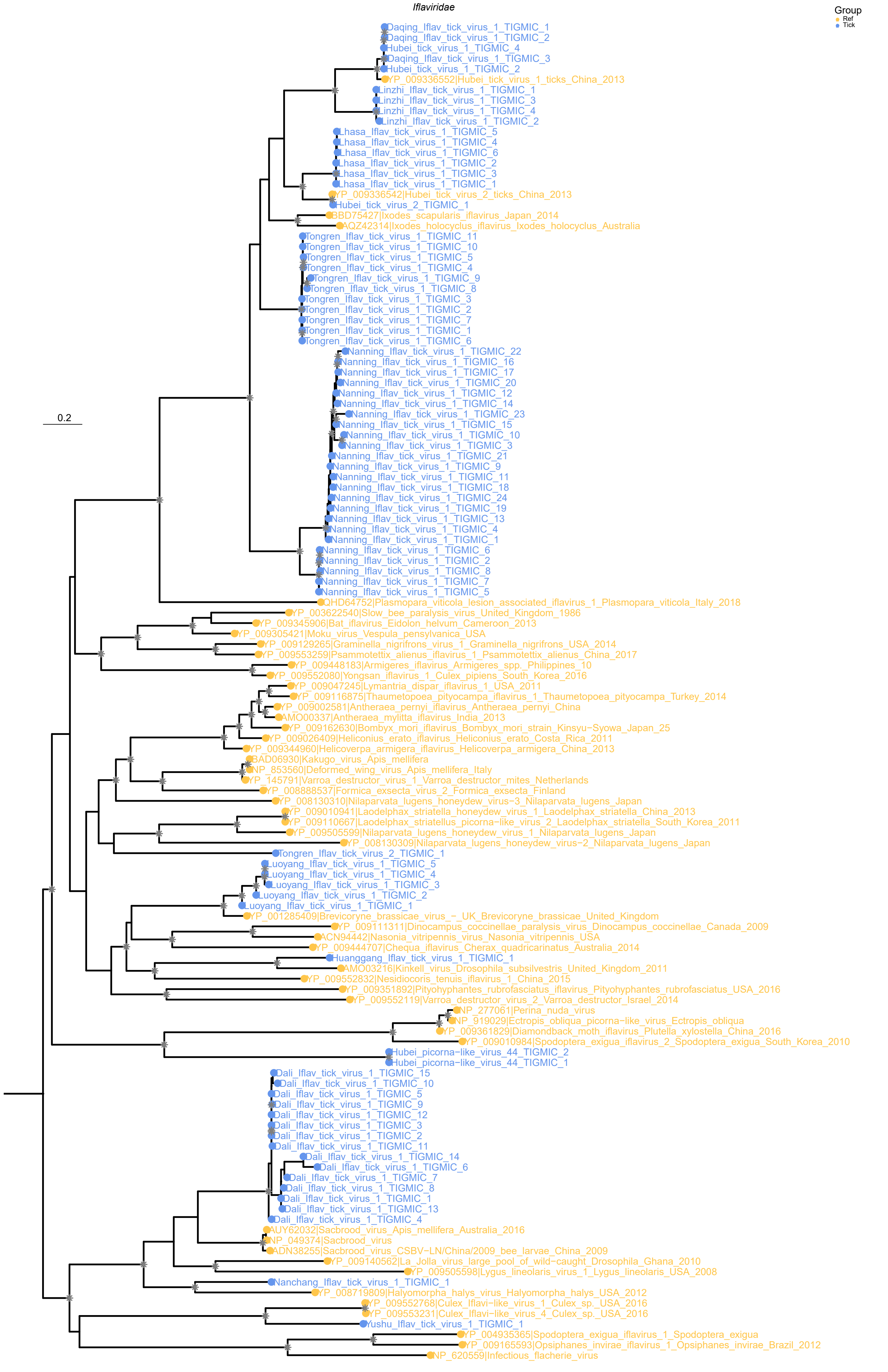
Iflaviridae
Group
Ref
Tick
0.2
Daqing_Iflav_tick_virus_1_TIGMIC_1
Daqing_Iflav_tick_virus_1_TIGMIC_2
Hubei_tick_virus_1_TIGMIC_4
Daqing_Iflav_tick_virus_1_TIGMIC_3
Hubei_tick_virus_1_TIGMIC_2
YP_009336552|Hubei_tick_virus_1_ticks_China_2013
Linzhi_Iflav_tick_virus_1_TIGMIC_1
Linzhi_Iflav_tick_virus_1_TIGMIC_3
Linzhi_Iflav_tick_virus_1_TIGMIC_4
Linzhi_Iflav_tick_virus_1_TIGMIC_2
Lhasa_Iflav_tick_virus_1_TIGMIC_5
Lhasa_Iflav_tick_virus_1_TIGMIC_4
Lhasa_Iflav_tick_virus_1_TIGMIC_6
Lhasa_Iflav_tick_virus_1_TIGMIC_2
Lhasa_Iflav_tick_virus_1_TIGMIC_3
Lhasa_Iflav_tick_virus_1_TIGMIC_1
YP_009336542|Hubei_tick_virus_2_ticks_China_2013
Hubei_tick_virus_2_TIGMIC_1
BBD75427|Ixodes_scapularis_iflavirus_Japan_2014
AQZ42314|Ixodes_holocyclus_iflavirus_Ixodes_holocyclus_Australia
Tongren_Iflav_tick_virus_1_TIGMIC_11
Tongren_Iflav_tick_virus_1_TIGMIC_10
Tongren_Iflav_tick_virus_1_TIGMIC_5
Tongren_Iflav_tick_virus_1_TIGMIC_4
Tongren_Iflav_tick_virus_1_TIGMIC_9
Tongren_Iflav_tick_virus_1_TIGMIC_8
Tongren_Iflav_tick_virus_1_TIGMIC_3
Tongren_Iflav_tick_virus_1_TIGMIC_2
Tongren_Iflav_tick_virus_1_TIGMIC_7
Tongren_Iflav_tick_virus_1_TIGMIC_1
Tongren_Iflav_tick_virus_1_TIGMIC_6
Nanning_Iflav_tick_virus_1_TIGMIC_22
Nanning_Iflav_tick_virus_1_TIGMIC_16
Nanning_Iflav_tick_virus_1_TIGMIC_17
Nanning_Iflav_tick_virus_1_TIGMIC_20
Nanning_Iflav_tick_virus_1_TIGMIC_12
Nanning_Iflav_tick_virus_1_TIGMIC_14
Nanning_Iflav_tick_virus_1_TIGMIC_23
Nanning_Iflav_tick_virus_1_TIGMIC_15
Nanning_Iflav_tick_virus_1_TIGMIC_10
Nanning_Iflav_tick_virus_1_TIGMIC_3
Nanning_Iflav_tick_virus_1_TIGMIC_21
Nanning_Iflav_tick_virus_1_TIGMIC_9
Nanning_Iflav_tick_virus_1_TIGMIC_11
Nanning_Iflav_tick_virus_1_TIGMIC_18
Nanning_Iflav_tick_virus_1_TIGMIC_24
Nanning_Iflav_tick_virus_1_TIGMIC_19
Nanning_Iflav_tick_virus_1_TIGMIC_13
Nanning_Iflav_tick_virus_1_TIGMIC_4
Nanning_Iflav_tick_virus_1_TIGMIC_1
Nanning_Iflav_tick_virus_1_TIGMIC_6
Nanning_Iflav_tick_virus_1_TIGMIC_2
Nanning_Iflav_tick_virus_1_TIGMIC_8
Nanning_Iflav_tick_virus_1_TIGMIC_7
Nanning_Iflav_tick_virus_1_TIGMIC_5
QHD64752|Plasmopara_viticola_lesion_associated_iflavirus_1_Plasmopara_viticola_Italy_2018
YP_003622540|Slow_bee_paralysis_virus_United_Kingdom_1986
YP_009345906|Bat_iflavirus_Eidolon_helvum_Cameroon_2013
YP_009305421|Moku_virus_Vespula_pensylvanica_USA
YP_009129265|Graminella_nigrifrons_virus_1_Graminella_nigrifrons_USA_2014
YP_009553259|Psammotettix_alienus_iflavirus_1_Psammotettix_alienus_China_2017
YP_009448183|Armigeres_iflavirus_Armigeres_spp._Philippines_10
YP_009552080|Yongsan_iflavirus_1_Culex_pipiens_South_Korea_2016
YP_009047245|Lymantria_dispar_iflavirus_1_USA_2011
YP_009116875|Thaumetopoea_pityocampa_iflavirus_1_Thaumetopoea_pityocampa_Turkey_2014
YP_009002581|Antheraea_pernyi_iflavirus_Antheraea_pernyi_China
AMO00337|Antheraea_mylitta_iflavirus_India_2013
YP_009162630|Bombyx_mori_iflavirus_Bombyx_mori_strain_Kinsyu-Syowa_Japan_25
YP_009026409|Heliconius_erato_iflavirus_Heliconius_erato_Costa_Rica_2011
YP_009344960|Helicoverpa_armigera_iflavirus_Helicoverpa_armigera_China_2013
BAD06930|Kakugo_virus_Apis_mellifera
NP_853560|Deformed_wing_virus_Apis_mellifera_Italy
YP_145791|Varroa_destructor_virus_1_Varroa_destructor_mites_Netherlands
YP_008888537|Formica_exsecta_virus_2_Formica_exsecta_Finland
YP_008130310|Nilaparvata_lugens_honeydew_virus-3_Nilaparvata_lugens_Japan
YP_009010941|Laodelphax_striatella_honeydew_virus_1_Laodelphax_striatella_China_2013
YP_009110667|Laodelphax_striatellus_picorna-like_virus_2_Laodelphax_striatella_South_Korea_2011
YP_009505599|Nilaparvata_lugens_honeydew_virus_1_Nilaparvata_lugens_Japan
YP_008130309|Nilaparvata_lugens_honeydew_virus-2_Nilaparvata_lugens_Japan
Tongren_Iflav_tick_virus_2_TIGMIC_1
Luoyang_Iflav_tick_virus_1_TIGMIC_5
Luoyang_Iflav_tick_virus_1_TIGMIC_4
Luoyang_Iflav_tick_virus_1_TIGMIC_3
Luoyang_Iflav_tick_virus_1_TIGMIC_2
Luoyang_Iflav_tick_virus_1_TIGMIC_1
YP_001285409|Brevicoryne_brassicae_virus_-_UK_Brevicoryne_brassicae_United_Kingdom
YP_009111311|Dinocampus_coccinellae_paralysis_virus_Dinocampus_coccinellae_Canada_2009
ACN94442|Nasonia_vitripennis_virus_Nasonia_vitripennis_USA
YP_009444707|Chequa_iflavirus_Cherax_quadricarinatus_Australia_2014
Huanggang_Iflav_tick_virus_1_TIGMIC_1
AMO03216|Kinkell_virus_Drosophila_subsilvestris_United_Kingdom_2011
YP_009552832|Nesidiocoris_tenuis_iflavirus_1_China_2015
YP_009351892|Pityohyphantes_rubrofasciatus_iflavirus_Pityohyphantes_rubrofasciatus_USA_2016
YP_009552119|Varroa_destructor_virus_2_Varroa_destructor_Israel_2014
NP_277061|Perina_nuda_virus
NP_919029|Ectropis_obliqua_picorna-like_virus_Ectropis_obliqua
YP_009361829|Diamondback_moth_iflavirus_Plutella_xylostella_China_2016
YP_009010984|Spodoptera_exigua_iflavirus_2_Spodoptera_exigua_South_Korea_2010
Hubei_picorna-like_virus_44_TIGMIC_2
Hubei_picorna-like_virus_44_TIGMIC_1
Dali_Iflav_tick_virus_1_TIGMIC_15
Dali_Iflav_tick_virus_1_TIGMIC_10
Dali_Iflav_tick_virus_1_TIGMIC_5
Dali_Iflav_tick_virus_1_TIGMIC_9
Dali_Iflav_tick_virus_1_TIGMIC_12
Dali_Iflav_tick_virus_1_TIGMIC_3
Dali_Iflav_tick_virus_1_TIGMIC_2
Dali_Iflav_tick_virus_1_TIGMIC_11
Dali_Iflav_tick_virus_1_TIGMIC_14
Dali_Iflav_tick_virus_1_TIGMIC_6
Dali_Iflav_tick_virus_1_TIGMIC_7
Dali_Iflav_tick_virus_1_TIGMIC_8
Dali_Iflav_tick_virus_1_TIGMIC_1
Dali_Iflav_tick_virus_1_TIGMIC_13
Dali_Iflav_tick_virus_1_TIGMIC_4
AUY62032|Sacbrood_virus_Apis_mellifera_Australia_2016
NP_049374|Sacbrood_virus
ADN38255|Sacbrood_virus_CSBV-LN/China/2009_bee_larvae_China_2009
YP_009140562|La_Jolla_virus_large_pool_of_wild-caught_Drosophila_Ghana_2010
YP_009505598|Lygus_lineolaris_virus_1_Lygus_lineolaris_USA_2008
Nanchang_Iflav_tick_virus_1_TIGMIC_1
YP_008719809|Halyomorpha_halys_virus_Halyomorpha_halys_USA_2012
YP_009552768|Culex_Iflavi-like_virus_1_Culex_sp._USA_2016
YP_009553231|Culex_Iflavi-like_virus_4_Culex_sp._USA_2016
Yushu_Iflav_tick_virus_1_TIGMIC_1
YP_004935365|Spodoptera_exigua_iflavirus_1_Spodoptera_exigua
YP_009165593|Opsiphanes_invirae_iflavirus_1_Opsiphanes_invirae_Brazil_2012
NP_620559|Infectious_flacherie_virus

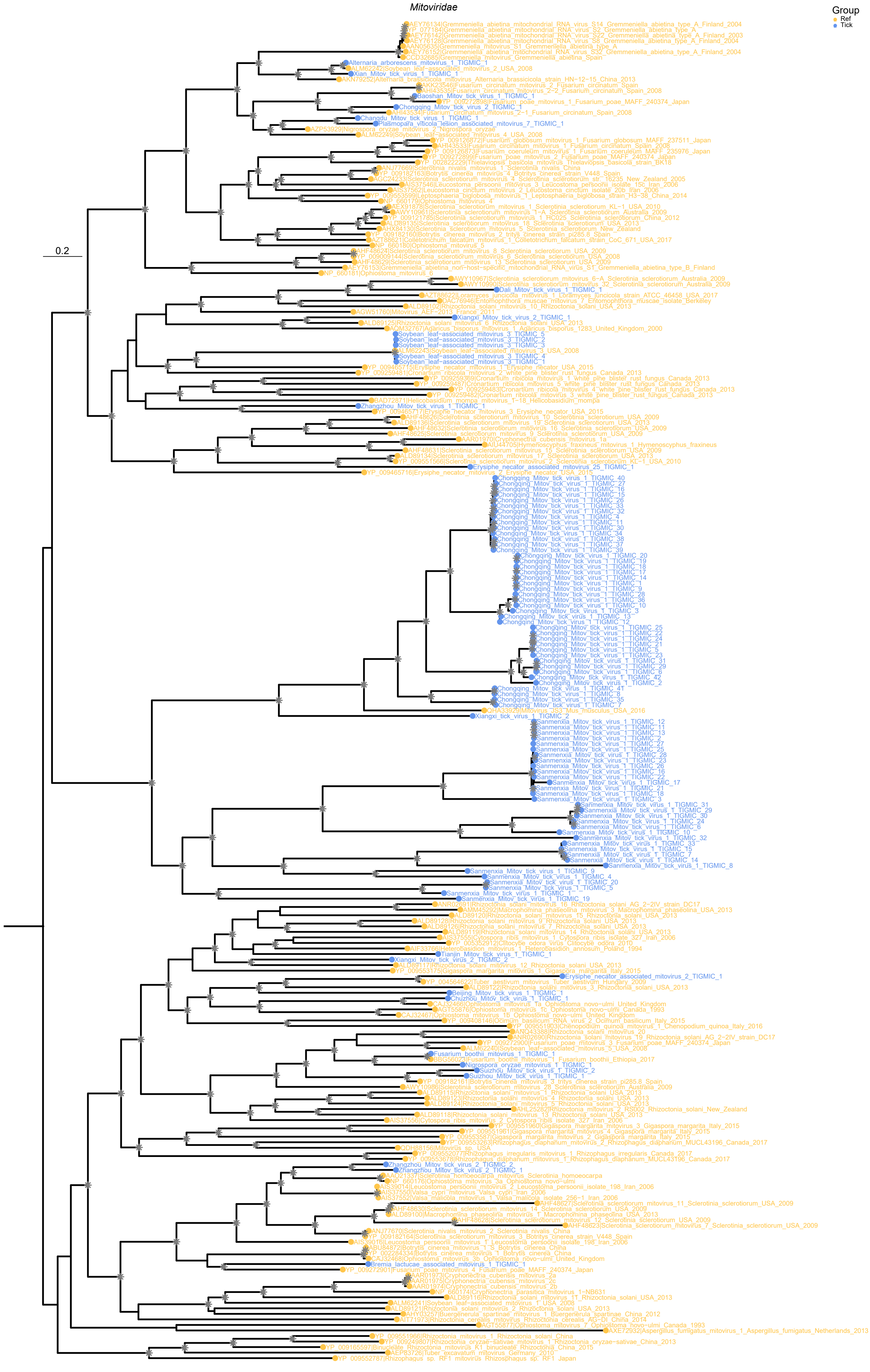

Mitoviridae
Group
Ref
Tick
0.2

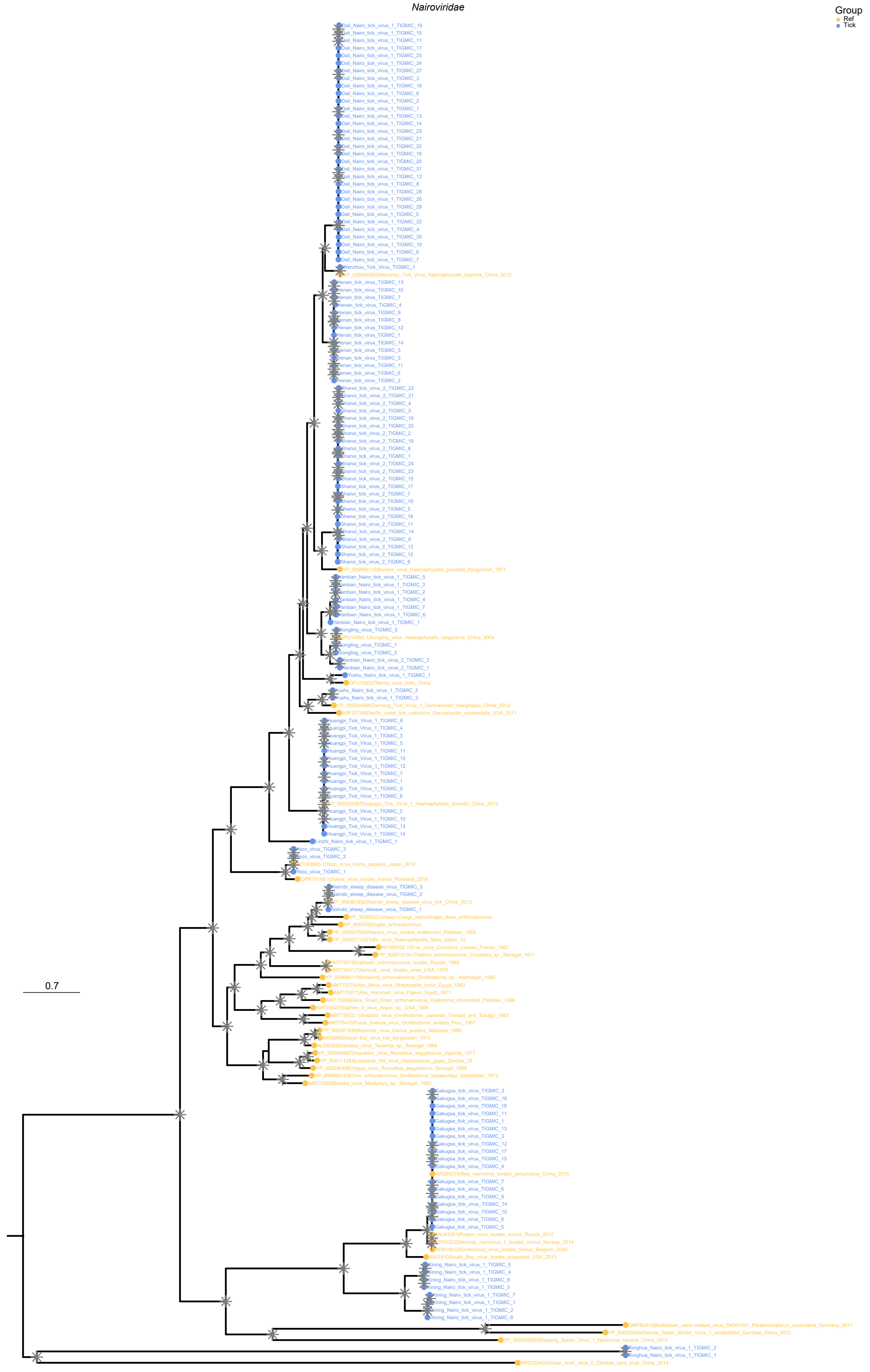
Nairoviridae
Group
Ref
Tick
Dali_Nairo_tick_virus_1_TIGMIC_16
Dali_Nairo_tick_virus_1_TIGMIC_15
Dali_Nairo_tick_virus_1_TIGMIC_11
Dali_Nairo_tick_virus_1_TIGMIC_17
Dali_Nairo_tick_virus_1_TIGMIC_25
Dali_Nairo_tick_virus_1_TIGMIC_24
Dali_Nairo_tick_virus_1_TIGMIC_27
Dali_Nairo_tick_virus_1_TIGMIC_3
Dali_Nairo_tick_virus_1_TIGMIC_19
Dali_Nairo_tick_virus_1_TIGMIC_6
Dali_Nairo_tick_virus_1_TIGMIC_2
Dali_Nairo_tick_virus_1_TIGMIC_1
Dali_Nairo_tick_virus_1_TIGMIC_13
Dali_Nairo_tick_virus_1_TIGMIC_14
Dali_Nairo_tick_virus_1_TIGMIC_23
Dali_Nairo_tick_virus_1_TIGMIC_21
Dali_Nairo_tick_virus_1_TIGMIC_32
Dali_Nairo_tick_virus_1_TIGMIC_18
Dali_Nairo_tick_virus_1_TIGMIC_20
Dali_Nairo_tick_virus_1_TIGMIC_31
Dali_Nairo_tick_virus_1_TIGMIC_12
Dali_Nairo_tick_virus_1_TIGMIC_8
Dali_Nairo_tick_virus_1_TIGMIC_28
Dali_Nairo_tick_virus_1_TIGMIC_26
Dali_Nairo_tick_virus_1_TIGMIC_29
Dali_Nairo_tick_virus_1_TIGMIC_5
Dali_Nairo_tick_virus_1_TIGMIC_22
Dali_Nairo_tick_virus_1_TIGMIC_4
Dali_Nairo_tick_virus_1_TIGMIC_30
Dali_Nairo_tick_virus_1_TIGMIC_10
Dali_Nairo_tick_virus_1_TIGMIC_9
Dali_Nairo_tick_virus_1_TIGMIC_7
Wenzhou_Tick_Virus_TIGMIC_1
YP_009304993|Wenzhou_Tick_Virus_Haemaphysalis_hystricis_China_2012
Henan_tick_virus_TIGMIC_13
Henan_tick_virus_TIGMIC_10
Henan_tick_virus_TIGMIC_7
Henan_tick_virus_TIGMIC_4
Henan_tick_virus_TIGMIC_9
Henan_tick_virus_TIGMIC_8
Henan_tick_virus_TIGMIC_12
Henan_tick_virus_TIGMIC_1
Henan_tick_virus_TIGMIC_14
Henan_tick_virus_TIGMIC_5
Henan_tick_virus_TIGMIC_3
Henan_tick_virus_TIGMIC_11
Henan_tick_virus_TIGMIC_6
Henan_tick_virus_TIGMIC_2
Shanxi_tick_virus_2_TIGMIC_22
Shanxi_tick_virus_2_TIGMIC_21
Shanxi_tick_virus_2_TIGMIC_4
Shanxi_tick_virus_2_TIGMIC_3
Shanxi_tick_virus_2_TIGMIC_16
Shanxi_tick_virus_2_TIGMIC_20
Shanxi_tick_virus_2_TIGMIC_2
Shanxi_tick_virus_2_TIGMIC_19
Shanxi_tick_virus_2_TIGMIC_8
Shanxi_tick_virus_2_TIGMIC_1
Shanxi_tick_virus_2_TIGMIC_24
Shanxi_tick_virus_2_TIGMIC_23
Shanxi_tick_virus_2_TIGMIC_15
Shanxi_tick_virus_2_TIGMIC_17
Shanxi_tick_virus_2_TIGMIC_7
Shanxi_tick_virus_2_TIGMIC_10
Shanxi_tick_virus_2_TIGMIC_5
Shanxi_tick_virus_2_TIGMIC_18
Shanxi_tick_virus_2_TIGMIC_11
Shanxi_tick_virus_2_TIGMIC_14
Shanxi_tick_virus_2_TIGMIC_6
Shanxi_tick_virus_2_TIGMIC_13
Shanxi_tick_virus_2_TIGMIC_12
Shanxi_tick_virus_2_TIGMIC_9
YP_009666118|Burana_virus_Haemaphysalis_punctata_Kyrgyzstan_1971
Yanbian_Nairo_tick_virus_1_TIGMIC_5
Yanbian_Nairo_tick_virus_1_TIGMIC_3
Yanbian_Nairo_tick_virus_1_TIGMIC_2
Yanbian_Nairo_tick_virus_1_TIGMIC_4
Yanbian_Nairo_tick_virus_1_TIGMIC_7
Yanbian_Nairo_tick_virus_1_TIGMIC_6
Yanbian_Nairo_tick_virus_1_TIGMIC_1
Songling_virus_TIGMIC_3
QPO14992.1|Songling_virus_Haemaphysalis_longicornis_China_2004
Songling_virus_TIGMIC_1
Songling_virus_TIGMIC_2
Yanbian_Nairo_tick_virus_2_TIGMIC_2
Yanbian_Nairo_tick_virus_2_TIGMIC_1
Yushu_Nairo_tick_virus_1_TIGMIC_1
QFU19352|Tamdy_virus_ticks_China
Yushu_Nairo_tick_virus_1_TIGMIC_2
Yushu_Nairo_tick_virus_1_TIGMIC_3
YP_009304988|Tacheng_Tick_Virus_1_Dermacentor_marginatus_China_2012
ARF07704|Pacific_coast_tick_nairovirus_Dermacentor_occidentalis_USA_2011
Huangpi_Tick_Virus_1_TIGMIC_6
Huangpi_Tick_Virus_1_TIGMIC_4
Huangpi_Tick_Virus_1_TIGMIC_3
Huangpi_Tick_Virus_1_TIGMIC_5
Huangpi_Tick_Virus_1_TIGMIC_11
Huangpi_Tick_Virus_1_TIGMIC_15
Huangpi_Tick_Virus_1_TIGMIC_12
Huangpi_Tick_Virus_1_TIGMIC_7
Huangpi_Tick_Virus_1_TIGMIC_1
Huangpi_Tick_Virus_1_TIGMIC_9
Huangpi_Tick_Virus_1_TIGMIC_8
YP_009293587|Huangpi_Tick_Virus_1_Haemaphysalis_doenitzi_China_2013
Huangpi_Tick_Virus_1_TIGMIC_2
Huangpi_Tick_Virus_1_TIGMIC_10
Huangpi_Tick_Virus_1_TIGMIC_13
Huangpi_Tick_Virus_1_TIGMIC_14
Linzhi_Nairo_tick_virus_1_TIGMIC_1
Yezo_virus_TIGMIC_3
Yezo_virus_TIGMIC_2
BCU08945.1|Yezo_virus_Homo_sapiens_Japan_2019
Yezo_virus_TIGMIC_1
QRR19149.1|Sulina_virus_Ixodes_ricinus_Romania_2016
Nairobi_sheep_disease_virus_TIGMIC_3
Nairobi_sheep_disease_virus_TIGMIC_2
YP_009361832|Nairobi_sheep_disease_virus_tick_China_2013
Nairobi_sheep_disease_virus_TIGMIC_1
YP_325663|Crimean-Congo_hemorrhagic_fever_orthonairovirus
NP_690576|Dugbe_orthonairovirus
YP_009507850|Hazara_virus_Ixodes_redikorzevi_Pakistan_1964
YP_009227122|Tofla_virus_Haemaphysalis_flava_Japan_12
AFH89032.1|Erve_virus_Crocidura_russula_France_1982
YP_009513191|Thiafora_orthonairovirus_Crocidura_sp._Senegal_1971
AMT75419|Sakhalin_orthonairovirus_Ixodes_Russia_1969
AMT75431|Tillamook_virus_Ixodes_uriae_USA_1970
YP_009666119|Artashat_orthonairovirus_Ornithodoros_sp._Azerbaijan_1985
AMT75374|Abu_Mina_virus_Streptopelia_turtur_Egypt_1963
AMT75371|Abu_Hammad_virus_Pigeon_Egypt_1971
AMT75389|Dera_Ghazi_Khan_orthonairovirus_Hyalomma_dromedarii_Pakistan_1966
AMT75422|Saphire_II_virus_Argas_sp._USA_1969
AMT75425.1|Soldado_virus_Ornithodoros_capensis_Trinidad_and_Tobago_1963
AMT75410|Punta_Salinas_virus_Ornithodoros_amblus_Peru_1967
YP_009361838|Keterah_virus_Cariosl_pusillus_Malaysia_1966
AKI29982|Issyk-Kul_virus_bat_Kyrgyzstan_1973
ALD83626|Gossas_virus_Tadarida_sp._Senegal_1964
YP_009449567|Kasokero_virus_Rousettus_aegyptiacus_Uganda_1977
YP_009111284|Leopards_Hill_virus_Hipposideros_gigas_Zambia_29
YP_009246486|Yogue_virus_Rousettus_aegyptiacus_Senegal_1968
YP_009666113|Chim_orthonairovirus_Ornithodoros_tartakovskyi_Uzbekistan_1972
AMT75383|Bandia_virus_Mastomys_sp._Senegal_1965
Gakugsa_tick_virus_TIGMIC_2
Gakugsa_tick_virus_TIGMIC_16
Gakugsa_tick_virus_TIGMIC_18
Gakugsa_tick_virus_TIGMIC_11
Gakugsa_tick_virus_TIGMIC_1
Gakugsa_tick_virus_TIGMIC_13
Gakugsa_tick_virus_TIGMIC_3
Gakugsa_tick_virus_TIGMIC_12
Gakugsa_tick_virus_TIGMIC_17
Gakugsa_tick_virus_TIGMIC_15
Gakugsa_tick_virus_TIGMIC_4
AXQ59276|Beiji_nairovirus_Ixodes_persulcatus_China_2015
Gakugsa_tick_virus_TIGMIC_7
Gakugsa_tick_virus_TIGMIC_6
Gakugsa_tick_virus_TIGMIC_9
Gakugsa_tick_virus_TIGMIC_14
Gakugsa_tick_virus_TIGMIC_10
Gakugsa_tick_virus_TIGMIC_8
Gakugsa_tick_virus_TIGMIC_5
ANJ43351|Pustyn_virus_Ixodes_ricinus_Russia_2015
ASY03232|Norway_nairovirus_1_Ixodes_ricinus_Norway_2014
ARB16032|Grotenhout_virus_Ixodes_ricinus_Belgium_2009
AII01810|South_Bay_virus_Ixodes_scapularis_USA_2013
Xining_Nairo_tick_virus_1_TIGMIC_5
Xining_Nairo_tick_virus_1_TIGMIC_4
Xining_Nairo_tick_virus_1_TIGMIC_8
Xining_Nairo_tick_virus_1_TIGMIC_3
Xining_Nairo_tick_virus_1_TIGMIC_7
Xining_Nairo_tick_virus_1_TIGMIC_1
Xining_Nairo_tick_virus_1_TIGMIC_2
Xining_Nairo_tick_virus_1_TIGMIC_6
QMP82413|Blattodean_nairo-related_virus_OKIAV321_Paratemnopteryx_couloniana_Germany_2011
YP_009293594|Sanxia_Water_Strider_Virus_1_unidentified_Gerridae_China_2012
YP_009300660|Shayang_Spider_Virus_1_Neoscona_nautica_China_2012
Tonghua_Nairo_tick_virus_1_TIGMIC_2
Tonghua_Nairo_tick_virus_1_TIGMIC_1
APG79342|Wuhan_snail_virus_2_Chinese_land_snail_China_2014
0.7

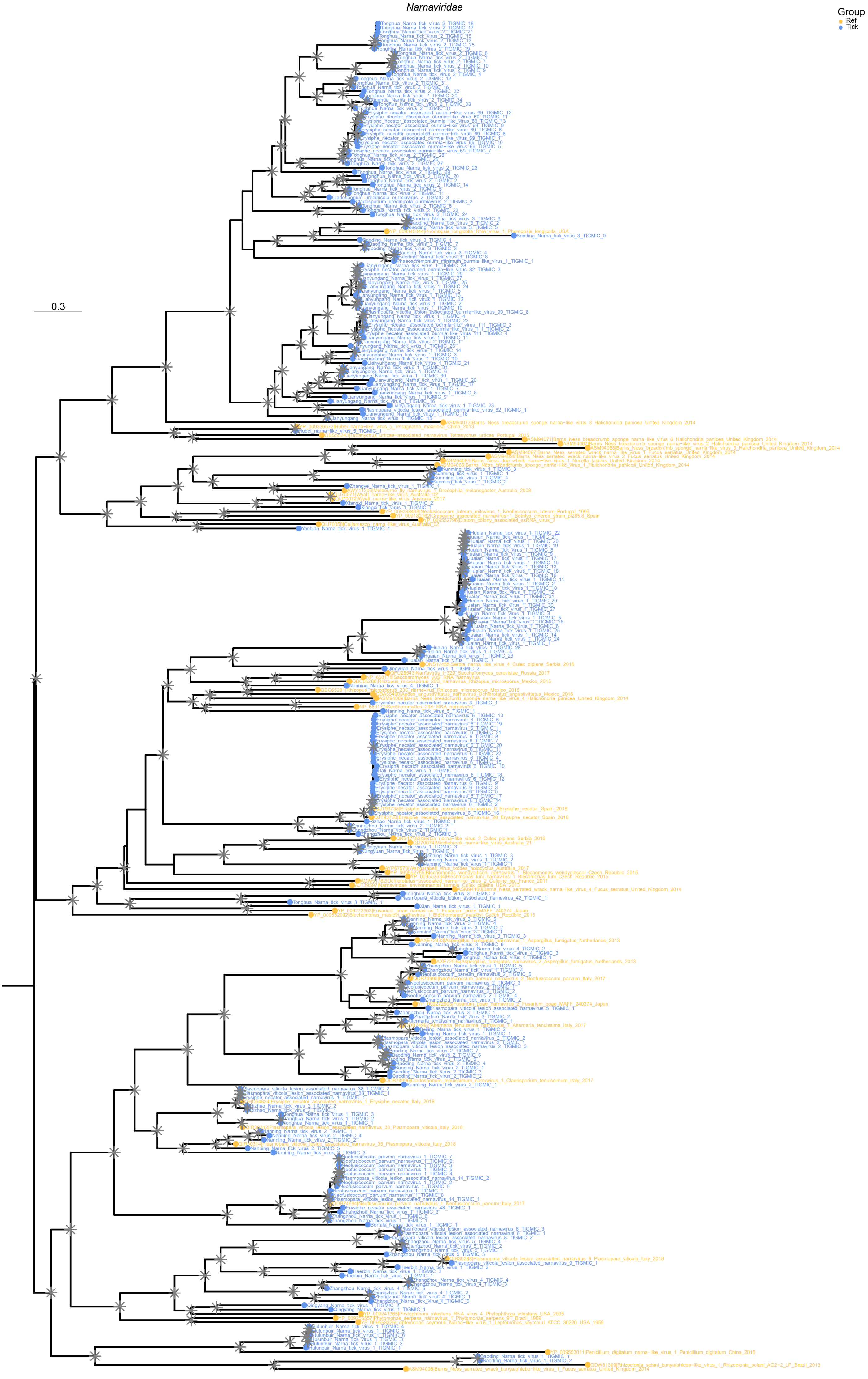
Narnaviridae
Group
Ref
Tick
0.3

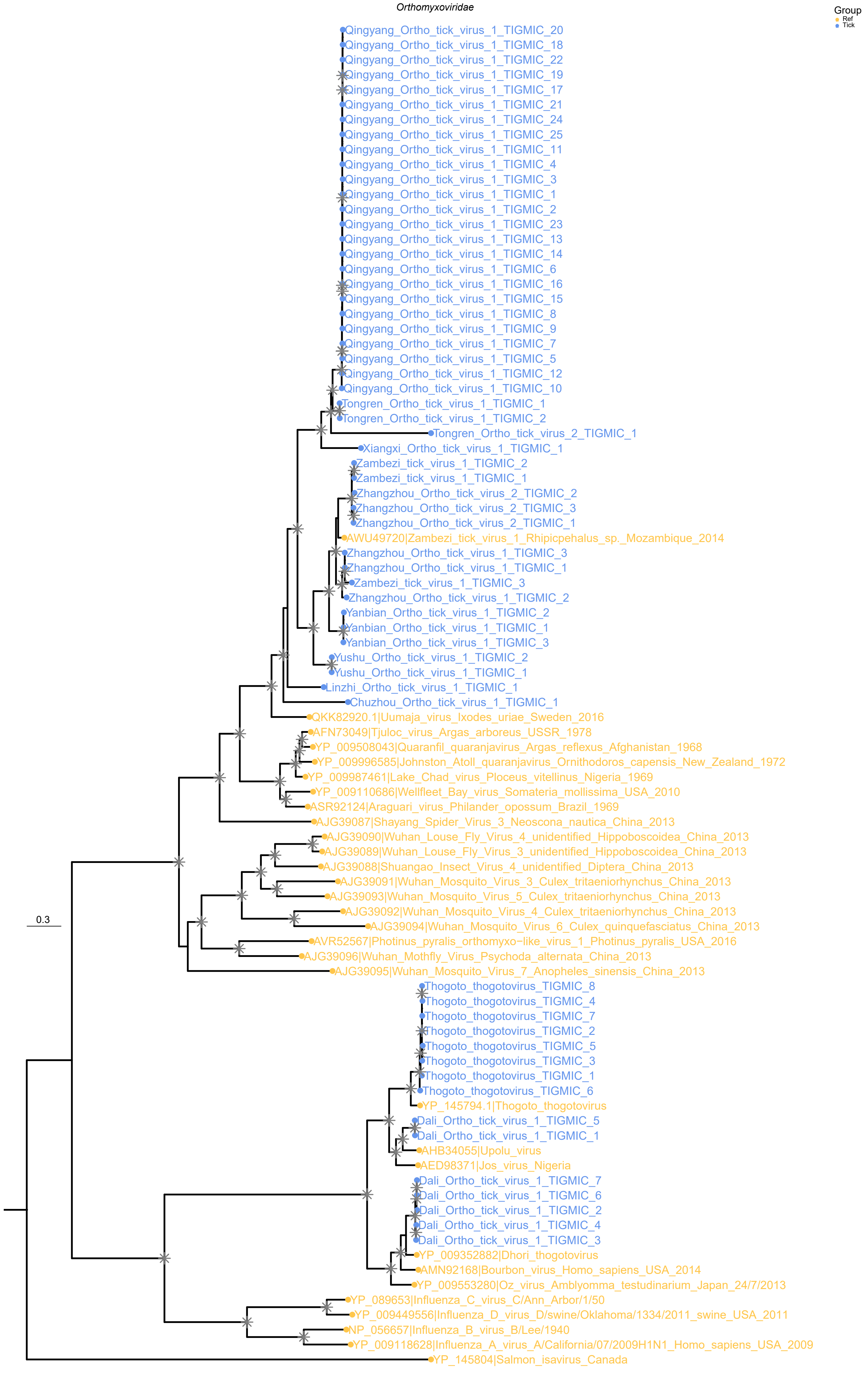
Orthomyxoviridae
Group
Ref
Tick
0.3
Qingyang_Ortho_tick_virus_1_TIGMIC_20
Qingyang_Ortho_tick_virus_1_TIGMIC_18
Qingyang_Ortho_tick_virus_1_TIGMIC_22
Qingyang_Ortho_tick_virus_1_TIGMIC_19
Qingyang_Ortho_tick_virus_1_TIGMIC_17
Qingyang_Ortho_tick_virus_1_TIGMIC_21
Qingyang_Ortho_tick_virus_1_TIGMIC_24
Qingyang_Ortho_tick_virus_1_TIGMIC_25
Qingyang_Ortho_tick_virus_1_TIGMIC_11
Qingyang_Ortho_tick_virus_1_TIGMIC_4
Qingyang_Ortho_tick_virus_1_TIGMIC_3
Qingyang_Ortho_tick_virus_1_TIGMIC_1
Qingyang_Ortho_tick_virus_1_TIGMIC_2
Qingyang_Ortho_tick_virus_1_TIGMIC_23
Qingyang_Ortho_tick_virus_1_TIGMIC_13
Qingyang_Ortho_tick_virus_1_TIGMIC_14
Qingyang_Ortho_tick_virus_1_TIGMIC_6
Qingyang_Ortho_tick_virus_1_TIGMIC_16
Qingyang_Ortho_tick_virus_1_TIGMIC_15
Qingyang_Ortho_tick_virus_1_TIGMIC_8
Qingyang_Ortho_tick_virus_1_TIGMIC_9
Qingyang_Ortho_tick_virus_1_TIGMIC_7
Qingyang_Ortho_tick_virus_1_TIGMIC_5
Qingyang_Ortho_tick_virus_1_TIGMIC_12
Qingyang_Ortho_tick_virus_1_TIGMIC_10
Tongren_Ortho_tick_virus_1_TIGMIC_1
Tongren_Ortho_tick_virus_1_TIGMIC_2
Tongren_Ortho_tick_virus_2_TIGMIC_1
Xiangxi_Ortho_tick_virus_1_TIGMIC_1
Zambezi_tick_virus_1_TIGMIC_2
Zambezi_tick_virus_1_TIGMIC_1
Zhangzhou_Ortho_tick_virus_2_TIGMIC_2
Zhangzhou_Ortho_tick_virus_2_TIGMIC_3
Zhangzhou_Ortho_tick_virus_2_TIGMIC_1
AWU49720|Zambezi_tick_virus_1_Rhipicpehalus_sp._Mozambique_2014
Zhangzhou_Ortho_tick_virus_1_TIGMIC_3
Zhangzhou_Ortho_tick_virus_1_TIGMIC_1
Zambezi_tick_virus_1_TIGMIC_3
Zhangzhou_Ortho_tick_virus_1_TIGMIC_2
Yanbian_Ortho_tick_virus_1_TIGMIC_2
Yanbian_Ortho_tick_virus_1_TIGMIC_1
Yanbian_Ortho_tick_virus_1_TIGMIC_3
Yushu_Ortho_tick_virus_1_TIGMIC_2
Yushu_Ortho_tick_virus_1_TIGMIC_1
Linzhi_Ortho_tick_virus_1_TIGMIC_1
Chuzhou_Ortho_tick_virus_1_TIGMIC_1
QKK82920.1|Uumaja_virus_Ixodes_uriae_Sweden_2016
AFN73049|Tjuloc_virus_Argas_arboreus_USSR_1978
YP_009508043|Quaranfil_quaranjavirus_Argas_reflexus_Afghanistan_1968
YP_009996585|Johnston_Atoll_quaranjavirus_Ornithodoros_capensis_New_Zealand_1972
YP_009987461|Lake_Chad_virus_Ploceus_vitellinus_Nigeria_1969
YP_009110686|Wellfleet_Bay_virus_Somateria_mollissima_USA_2010
ASR92124|Araguari_virus_Philander_opossum_Brazil_1969
AJG39087|Shayang_Spider_Virus_3_Neoscona_nautica_China_2013
AJG39090|Wuhan_Louse_Fly_Virus_4_unidentified_Hippoboscoidea_China_2013
AJG39089|Wuhan_Louse_Fly_Virus_3_unidentified_Hippoboscoidea_China_2013
AJG39088|Shuangao_Insect_Virus_4_unidentified_Diptera_China_2013
AJG39091|Wuhan_Mosquito_Virus_3_Culex_tritaeniorhynchus_China_2013
AJG39093|Wuhan_Mosquito_Virus_5_Culex_tritaeniorhynchus_China_2013
AJG39092|Wuhan_Mosquito_Virus_4_Culex_tritaeniorhynchus_China_2013
AJG39094|Wuhan_Mosquito_Virus_6_Culex_quinquefasciatus_China_2013
AVR52567|Photinus_pyralis_orthomyxo−like_virus_1_Photinus_pyralis_USA_2016
AJG39096|Wuhan_Mothfly_Virus_Psychoda_alternata_China_2013
AJG39095|Wuhan_Mosquito_Virus_7_Anopheles_sinensis_China_2013
Thogoto_thogotovirus_TIGMIC_8
Thogoto_thogotovirus_TIGMIC_4
Thogoto_thogotovirus_TIGMIC_7
Thogoto_thogotovirus_TIGMIC_2
Thogoto_thogotovirus_TIGMIC_5
Thogoto_thogotovirus_TIGMIC_3
Thogoto_thogotovirus_TIGMIC_1
Thogoto_thogotovirus_TIGMIC_6
YP_145794.1|Thogoto_thogotovirus
Dali_Ortho_tick_virus_1_TIGMIC_5
Dali_Ortho_tick_virus_1_TIGMIC_1
AHB34055|Upolu_virus
AED98371|Jos_virus_Nigeria
Dali_Ortho_tick_virus_1_TIGMIC_7
Dali_Ortho_tick_virus_1_TIGMIC_6
Dali_Ortho_tick_virus_1_TIGMIC_2
Dali_Ortho_tick_virus_1_TIGMIC_4
Dali_Ortho_tick_virus_1_TIGMIC_3
YP_009352882|Dhori_thogotovirus
AMN92168|Bourbon_virus_Homo_sapiens_USA_2014
YP_009553280|Oz_virus_Amblyomma_testudinarium_Japan_24/7/2013
YP_089653|Influenza_C_virus_C/Ann_Arbor/1/50
YP_009449556|Influenza_D_virus_D/swine/Oklahoma/1334/2011_swine_USA_2011
NP_056657|Influenza_B_virus_B/Lee/1940
YP_009118628|Influenza_A_virus_A/California/07/2009H1N1_Homo_sapiens_USA_2009
YP_145804|Salmon_isavirus_Canada

*Partitiviridae*

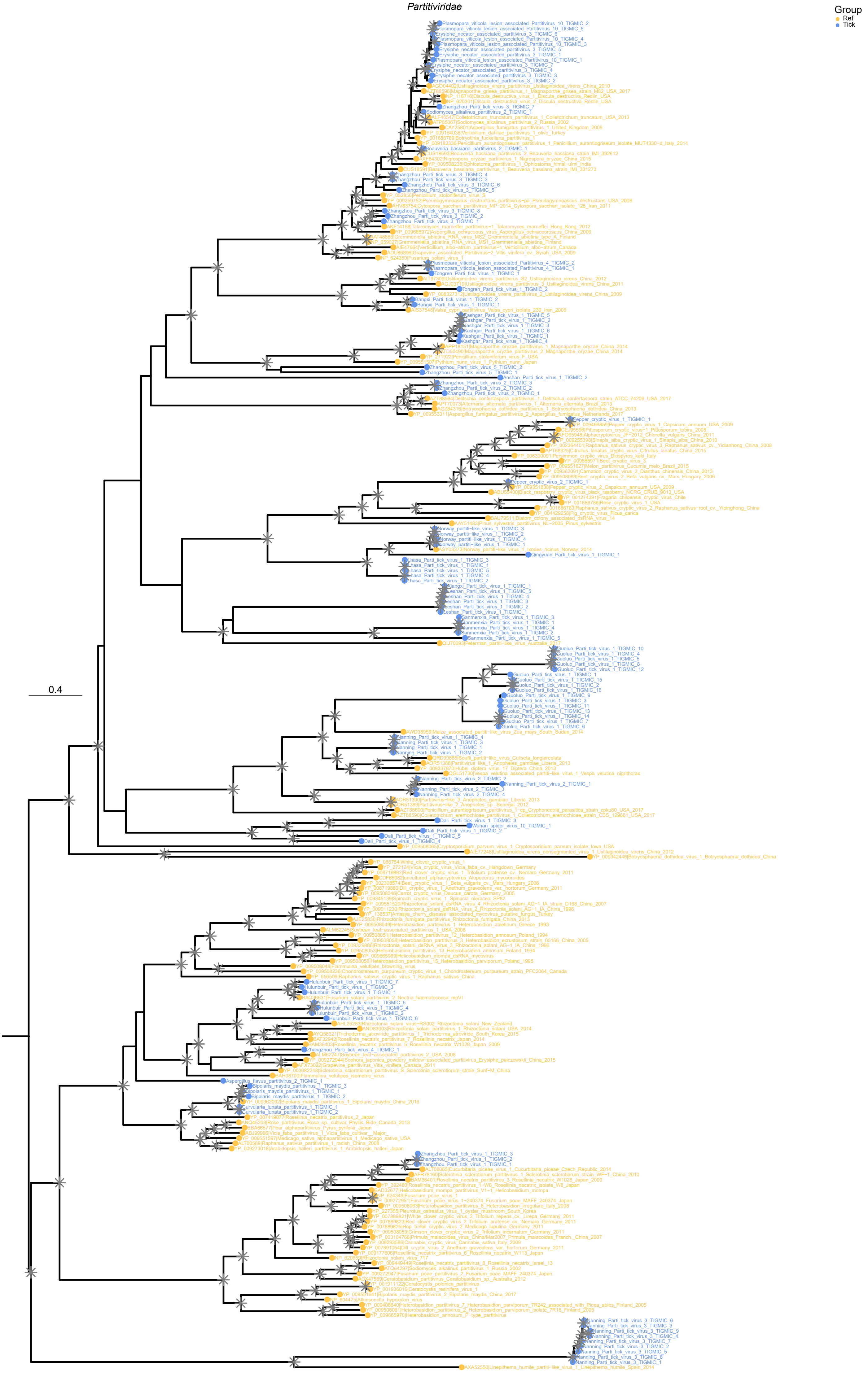

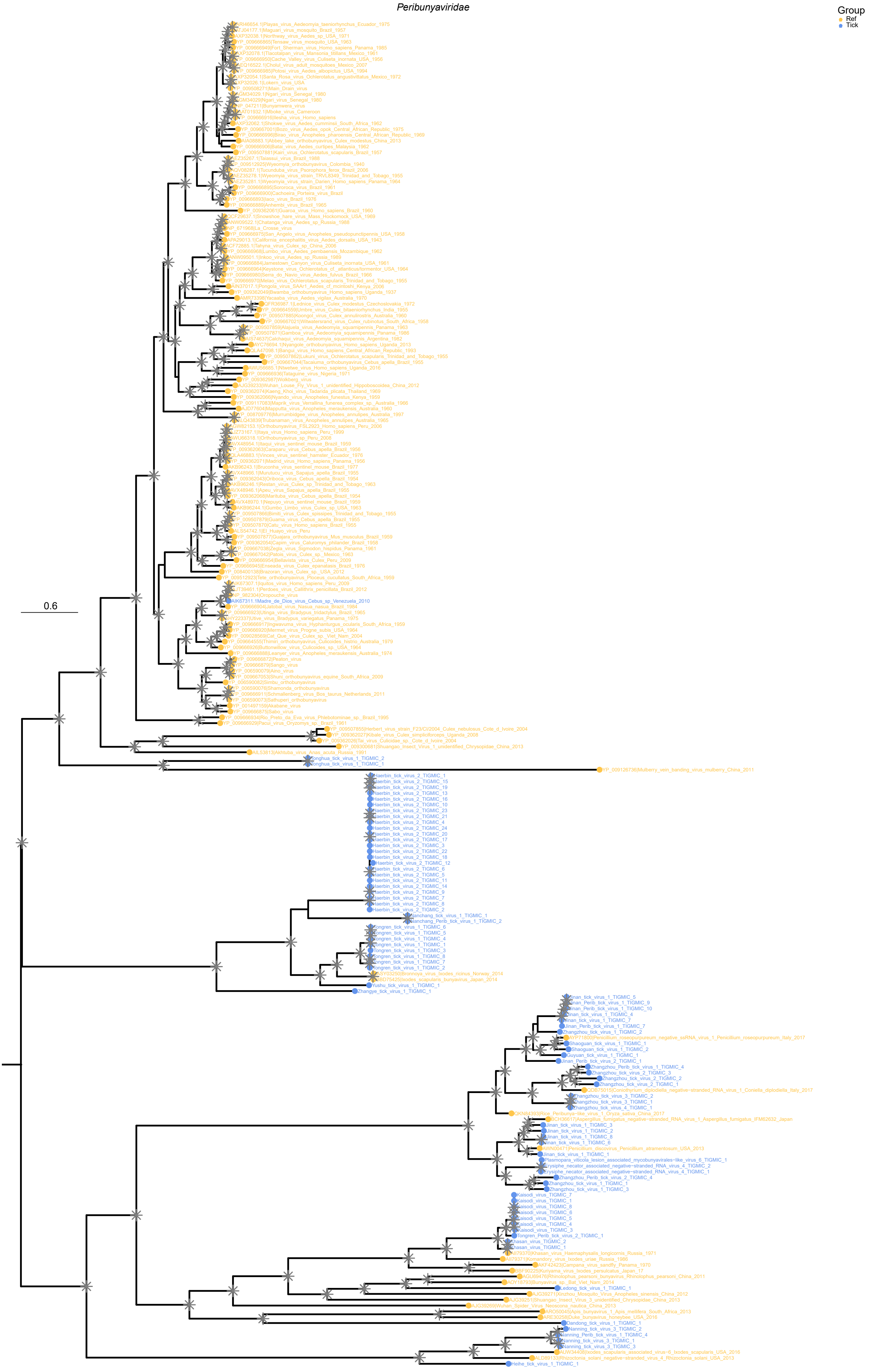
Peribunyaviridae
Group
Ref
Tick
0.6

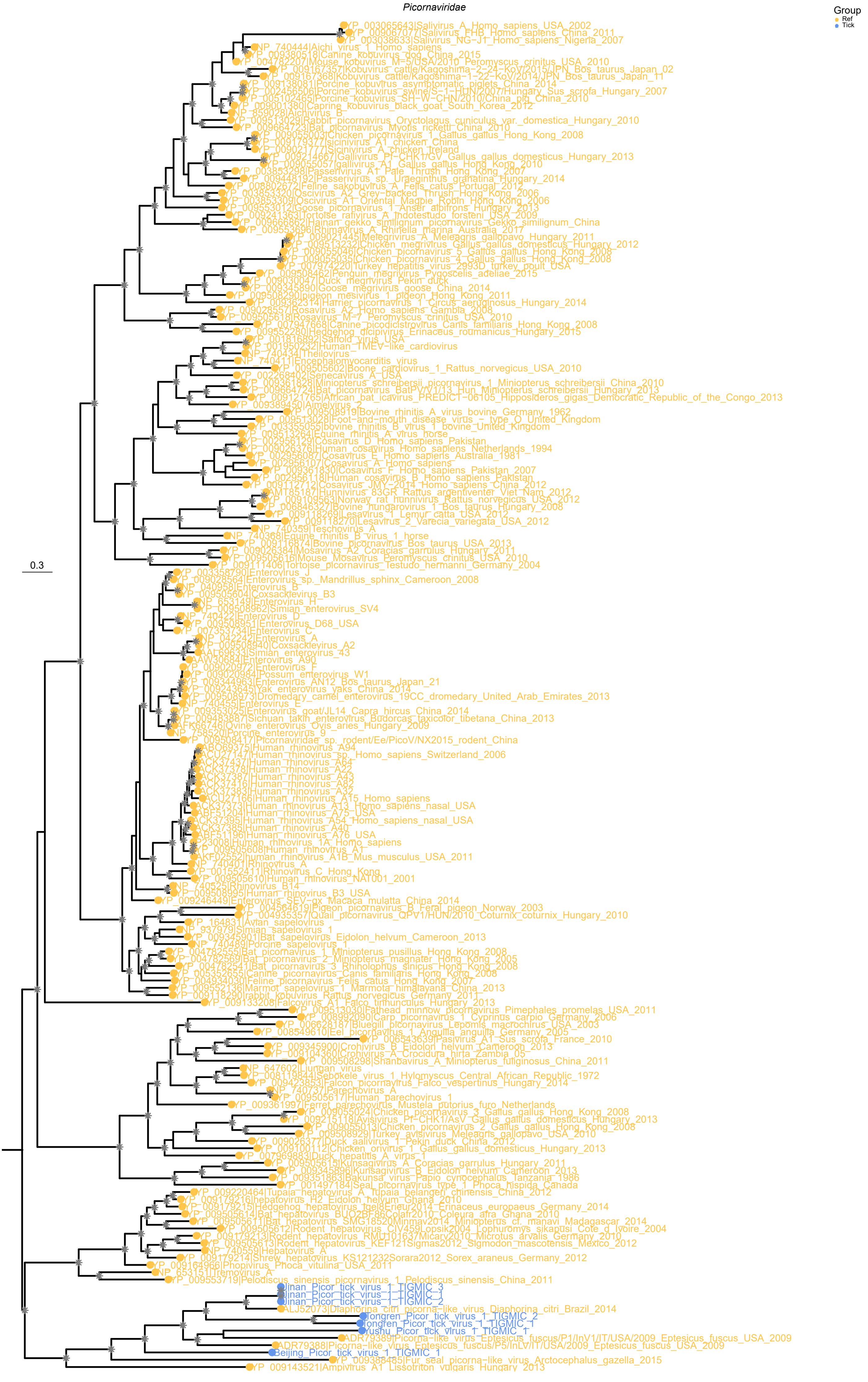
Picornaviridae
Group
Ref
Tick
0.3
YP_003065643|Salivirus_A_Homo_sapiens_USA_2002
YP_009067077|Salivirus_FHB_Homo_sapiens_China_2011
YP_003038633|Salivirus_NG-J1_Homo_sapiens_Nigeria_2007
NP_740444|Aichi_virus_1_Homo_sapiens
YP_009380518|Canine_kobuvirus_dog_China_2015
YP_004782207|Mouse_kobuvirus_M-5/USA/2010_Peromyscus_crinitus_USA_2010
YP_009167357|Kobuvirus_cattle/Kagoshima-2-24-KoV/2015/JPN_Bos_taurus_Japan_02
YP_009167368|Kobuvirus_cattle/Kagoshima-1-22-KoV/2014/JPN_Bos_taurus_Japan_11
YP_009138081|Porcine_kobuvirus_asymptomatic_piglets_China_2014
YP_002456506|Porcine_kobuvirus_swine/S-1-HUN/2007/Hungary_Sus_scrofa_Hungary_2007
YP_005102465|Porcine_kobuvirus_SH-W-CHN/2010/China_pig_China_2010
YP_009001380|Caprine_kobuvirus_black_goat_South_Korea_2012
YP_859029|Aichivirus_B
YP_009513029|Rabbit_picornavirus_Oryctolagus_cuniculus_var._domestica_Hungary_2010
YP_009664723|Bat_picornavirus_Myotis_ricketti_China_2010
YP_009055003|Chicken_picornavirus_1_Gallus_gallus_Hong_Kong_2008
YP_009179377|sicinivirus_A1_chicken_China
YP_009021777|Sicinivirus_A_chicken_Ireland
YP_009214667|Gallivirus_Pf-CHK1/GV_Gallus_gallus_domesticus_Hungary_2013
YP_009055057|gallivirus_A1_Gallus_gallus_Hong_Kong_2010
YP_003853298|Passerivirus_A1_Pale_Thrush_Hong_Kong_2007
YP_009448192|Passerivirus_sp._Uraeginthus_granatina_Hungary_2014
YP_008802672|Feline_sakobuvirus_A_Felis_catus_Portugal_2012
YP_003853320|Oscivirus_A2_Grey-backed_Thrush_Hong_Kong_2006
YP_003853309|Oscivirus_A1_Oriental_Magpie_Robin_Hong_Kong_2006
YP_009553012|Goose_picornavirus_1_Anser_albifrons_Hungary_2013
YP_009241363|Tortoise_rafivirus_A_Indotestudo_forsteni_USA_2009
YP_009666862|Hainan_gekko_similignum_picornavirus_Gekko_similignum_China
YP_009553696|Rhimavirus_A_Rhinella_marina_Australia_2017
YP_009021445|Melegrivirus_A_Meleagris_gallopavo_Hungary_2011
YP_009513232|Chicken_megrivirus_Gallus_gallus_domesticus_Hungary_2012
YP_009055046|Chicken_picornavirus_5_Gallus_gallus_Hong_Kong_2008
YP_009055035|Chicken_picornavirus_4_Gallus_gallus_Hong_Kong_2008
YP_007374220|Turkey_hepatitis_virus_2993D_turkey_poult_USA
YP_009508462|Penguin_megrivirus_Pygoscelis_adeliae_2015
YP_009030047|Duck_megrivirus_Pekin_duck
YP_009345890|Goose_megrivirus_goose_China_2014
YP_009508290|pigeon_mesivirus_1_pigeon_Hong_Kong_2011
YP_009362314|Harrier_picornavirus_1_Circus_aeruginosus_Hungary_2014
YP_009028557|Rosavirus_A2_Homo_sapiens_Gambia_2008
YP_009505618|Rosavirus_M-7_Peromyscus_crinitus_USA_2010
YP_007947668|Canine_picodicistrovirus_Canis_familiaris_Hong_Kong_2008
YP_009552280|Hedgehog_dicipivirus_Erinaceus_roumanicus_Hungary_2015
YP_001816892|Saffold_virus_USA
YP_001950232|Human_TMEV-like_cardiovirus
NP_740434|Theilovirus
NP_740411|Encephalomyocarditis_virus
YP_009505602|Boone_cardiovirus_1_Rattus_norvegicus_USA_2010
YP_002268402|Senecavirus_A_USA
YP_009361828|Miniopterus_schreibersii_picornavirus_1_Miniopterus_schreibersii_China_2010
YP_009664724|Bat_picornavirus_BatPV/V1/13_Hun_Miniopterus_schreibersii_Hungary_2013
YP_009121765|African_bat_icavirus_PREDICT-06105_Hipposideros_gigas_Democratic_Republic_of_the_Congo_2013
YP_009389450|Aimelvirus_2
YP_009508919|Bovine_rhinitis_A_virus_bovine_Germany_1962
YP_009513028|Foot-and-mouth_disease_virus_-_type_O_United_Kingdom
YP_003352055|Bovine_rhinitis_B_virus_1_bovine_United_Kingdom
YP_009513264|Equine_rhinitis_A_virus_horse
YP_002956129|Cosavirus_D_Homo_sapiens_Pakistan
YP_009026376|Human_cosavirus_Homo_sapiens_Netherlands_1994
YP_002956087|Cosavirus_E_Homo_sapiens_Australia_1981
YP_002956107|Cosavirus_A_Homo_sapiens
YP_009361830|Cosavirus_F_Homo_sapiens_Pakistan_2007
YP_002956118|Human_cosavirus_B_Homo_sapiens_Pakistan
YP_009112712|Cosavirus_JMY-2014_Homo_sapiens_China_2012
AMT85187|Hunnivirus_83GR_Rattus_argentiventer_Viet_Nam_2012
YP_009109563|Norway_rat_hunnivirus_Rattus_norvegicus_USA_2012
YP_006846327|Bovine_hungarovirus_1_Bos_taurus_Hungary_2008
YP_009118269|Lesavirus_1_Lemur_catta_USA_2012
YP_009118270|Lesavirus_2_Varecia_variegata_USA_2012
NP_740359|Teschovirus_A
NP_740368|Equine_rhinitis_B_virus_1_horse
YP_009116874|Bovine_picornavirus_Bos_taurus_USA_2013
YP_009026384|Mosavirus_A2_Coracias_garrulus_Hungary_2011
YP_009505616|Mouse_Mosavirus_Peromyscus_crinitus_USA_2010
YP_009111406|Tortoise_picornavirus_Testudo_hermanni_Germany_2004
YP_003358790|Enterovirus_J
YP_009028564|Enterovirus_sp._Mandrillus_sphinx_Cameroon_2008
NP_040958|Enterovirus_B
YP_009505604|Coxsackievirus_B3
NP_653149|Enterovirus_H
YP_009508962|Simian_enterovirus_SV4
NP_740422|Enterovirus_D
YP_009508951|Enterovirus_D68_USA
YP_007353734|Enterovirus_C
NP_042242|Enterovirus_A
YP_009508940|Coxsackievirus_A2
AAL69633|Simian_enterovirus_43
AAW30684|Enterovirus_A90
YP_009020972|Enterovirus_F
YP_009020984|Possum_enterovirus_W1
YP_009344963|Enterovirus_AN12_Bos_taurus_Japan_21
YP_009243645|Yak_enterovirus_yaks_China_2014
YP_009508973|Dromedary_camel_enterovirus_19CC_dromedary_United_Arab_Emirates_2013
NP_740455|Enterovirus_E
YP_009353025|Enterovirus_goat/JL14_Capra_hircus_China_2014
YP_009483887|Sichuan_takin_enterovirus_Budorcas_taxicolor_tibetana_China_2013
AFK66746|Ovine_enterovirus_Ovis_aries_Hungary_2009
NP_758520|Porcine_enterovirus_9
YP_009508417|Picornaviridae_sp._rodent/Ee/PicoV/NX2015_rodent_China
ABO69375|Human_rhinovirus_A94
ACU27147|Human_rhinovirus_sp._Homo_sapiens_Switzerland_2006
ACK37437|Human_rhinovirus_A64
ACK37378|Human_rhinovirus_A22
ACK37387|Human_rhinovirus_A43
ACK37419|Human_rhinovirus_A82
ACK37383|Human_rhinovirus_A32
ACU27166|Human_rhinovirus_A15_Homo_sapiens
ACK37373|Human_rhinovirus_A13_Homo_sapiens_nasal_USA
ABF51204|Human_rhinovirus_A75_USA
ACK37395|Human_rhinovirus_A54_Homo_sapiens_nasal_USA
ACK37385|Human_rhinovirus_A40
ABF51196|Human_rhinovirus_A76_USA
AAA23008|Human_rhinovirus_1A_Homo_sapiens
YP_009505608|Human_rhinovirus_A1
AKF02552|human_rhinovirus_A1B_Mus_musculus_USA_2011
NP_740401|Rhinovirus_A
YP_001552411|Rhinovirus_C_Hong_Kong
YP_009505610|Human_rhinovirus_NAT001_2001
NP_740525|Rhinovirus_B14
YP_009508995|Human_rhinovirus_B3_USA
YP_009246449|Enterovirus_SEV-gx_Macaca_mulatta_China_2014
YP_004564619|Pigeon_picornavirus_B_Feral_pigeon_Norway_2003
YP_004935357|Quail_picornavirus_QPV1/HUN/2010_Coturnix_coturnix_Hungary_2010
YP_164831|Avian_sapelovirus
NP_937979|Simian_sapelovirus_1
YP_009345901|Bat_sapelovirus_Eidolon_helvum_Cameroon_2013
NP_740489|Porcine_sapelovirus_1
YP_004782555|Bat_picornavirus_1_Miniopterus_pusillus_Hong_Kong_2008
YP_004782569|Bat_picornavirus_2_Miniopterus_magnater_Hong_Kong_2005
YP_004782541|Bat_picornavirus_3_Rhinolophus_sinicus_Hong_Kong_2008
YP_005352655|Canine_picornavirus_Canis_familiaris_Hong_Kong_2008
YP_004934030|Feline_picornavirus_Felis_catus_Hong_Kong_2007
YP_009552136|Marmot_sapelovirus_1_Marmota_himalayana_China_2013
YP_009118290|rabbit_kobuvirus_Rattus_norvegicus_Germany_2011
YP_009133208|Falcovirus_A1_Falco_tinnunculus_Hungary_2013
YP_009513030|Fathead_minnow_picornavirus_Pimephales_promelas_USA_2011
YP_008992090|Carp_picornavirus_1_Cyprinus_carpio_Germany_2006
YP_006628187|Bluegill_picornavirus_Lepomis_macrochirus_USA_2003
YP_008549610|Eel_picornavirus_1_Anguilla_anguilla_Germany_2005
YP_006543639|Pasivirus_A1_Sus_scrofa_France_2010
YP_009345900|Crohivirus_B_Eidolon_helvum_Cameroon_2013
YP_009104360|Crohivirus_A_Crocidura_hirta_Zambia_05
YP_009508298|Shanbavirus_A_Miniopterus_fuliginosus_China_2011
NP_647602|Ljungan_virus
YP_008119844|Sebokele_virus_1_Hylomyscus_Central_African_Republic_1972
YP_009423853|Falcon_picornavirus_Falco_vespertinus_Hungary_2014
NP_740737|Parechovirus_A
YP_009505617|Human_parechovirus_1
YP_009361997|Ferret_parechovirus_Mustela_putorius_furo_Netherlands
YP_009055024|Chicken_picornavirus_3_Gallus_gallus_Hong_Kong_2008
YP_009215118|Avisivirus_Pf-CHK1/AsV_Gallus_gallus_domesticus_Hungary_2013
YP_009055013|Chicken_picornavirus_2_Gallus_gallus_Hong_Kong_2008
YP_009508929|Turkey_avisivirus_Meleagris_gallopavo_USA_2010
YP_009026377|Duck_aalivirus_1_Pekin_duck_China_2012
YP_009100112|Chicken_orivirus_1_Gallus_gallus_domesticus_Hungary_2013
YP_007969883|Duck_hepatitis_A_virus_1
YP_009505615|Kunsagivirus_A_Coracias_garrulus_Hungary_2011
YP_009345896|Kunsagivirus_B_Eidolon_helvum_Cameroon_2013
YP_009351863|Bakunsa_virus_Papio_cynocephalus_Tanzania_1986
YP_001497184|Seal_picornavirus_type_1_Phoca_hispida_Canada
YP_009220464|Tupaia_hepatovirus_A_Tupaia_belangeri_chinensis_China_2012
YP_009179216|hepatovirus_H2_Eidolon_helvum_Ghana_2010
YP_009179215|Hedgehog_hepatovirus_Igel8Erinaceus2014_Erinaceus_europaeus_Germany_2014
YP_009505614|Bat_hepatovirus_BUO2BF86Colafr2010_Coleura_afra_Ghana_2010
YP_009505611|Bat_hepatovirus_SMG18520Minmav2014_Miniopterus_cf._manavi_Madagascar_2014
YP_009505612|Rodent_hepatovirus_CIV459Lopsik2004_Lophuromys_sikapusi_Cote_d_Ivoire_2004
YP_009179213|Rodent_hepatovirus_RMU101637Micarv2010_Microtus_arvalis_Germany_2010
YP_009505613|Rodent_hepatovirus_KEF121Sigmas2012_Sigmodon_mascotensis_Mexico_2012
NP_705553|Hepatovirus_A
YP_009179214|Shrew_hepatovirus_KS121232Sorara2012_Sorex_araneus_Germany_2012
YP_009164966|Phopivirus_Phoca_vitulina_USA_2011
NP_653151|Tremovirus_A
YP_009553719|Pelodiscus_sinensis_picornavirus_1_Pelodiscus_sinensis_China_2011
Jinan_Picor_tick_virus_1_TIGMIC_3
Jinan_Picor_tick_virus_1_TIGMIC_1
Jinan_Picor_tick_virus_1_TIGMIC_2
ALJ52073|Diaphorina_citri_picorna-like_virus_Diaphorina_citri_Brazil_2014
Tongren_Picor_tick_virus_1_TIGMIC_2
Tongren_Picor_tick_virus_1_TIGMIC_1
Yushu_Picor_tick_virus_1_TIGMIC_1
ADR79389|Picorna-like_virus_Eptesicus_fuscus/P1/InV1/IT/USA/2009_Eptesicus_fuscus_USA_2009
ADR79388|Picorna-like_virus_Eptesicus_fuscus/P5/InLV/IT/USA/2009_Eptesicus_fuscus_USA_2009
Beijing_Picor_tick_virus_1_TIGMIC_1
YP_009388485|Fur_seal_picorna-like_virus_Arctocephalus_gazella_2015
YP_009143521|Ampivirus_A1_Lissotriton_vulgaris_Hungary_2013

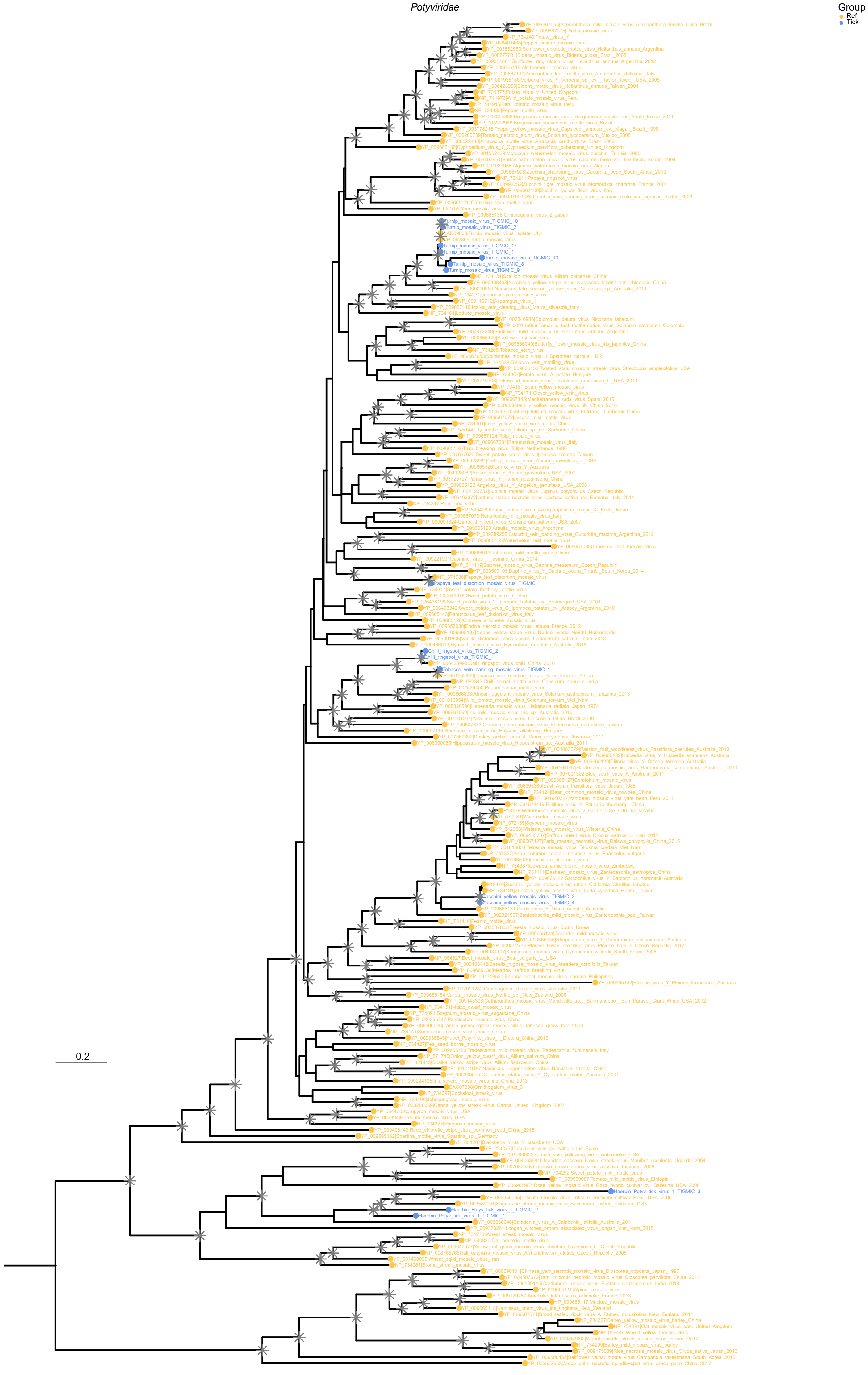
Potyviridae
Group
Ref
Tick
0.2

*Rhabdoviridae*

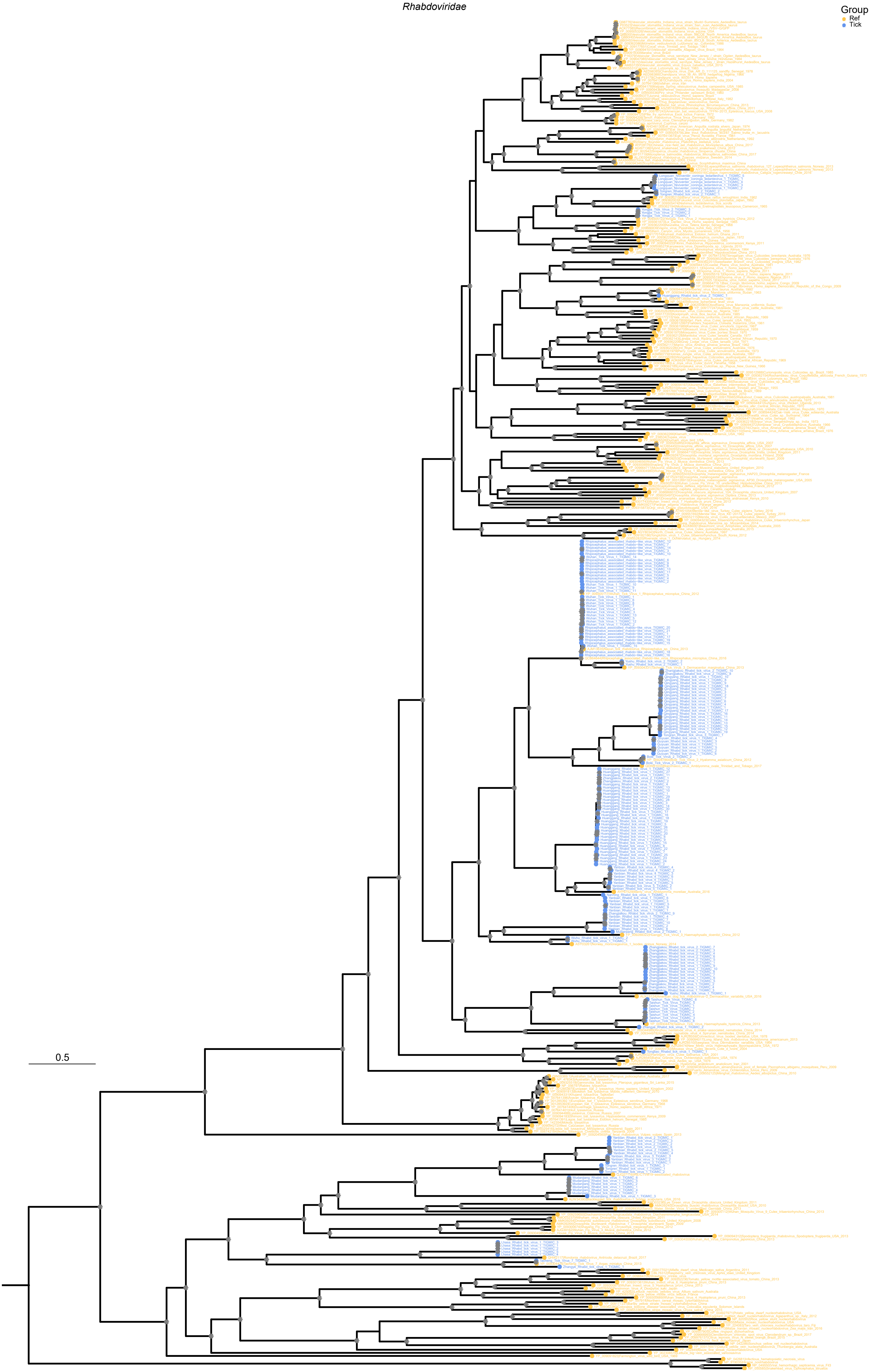

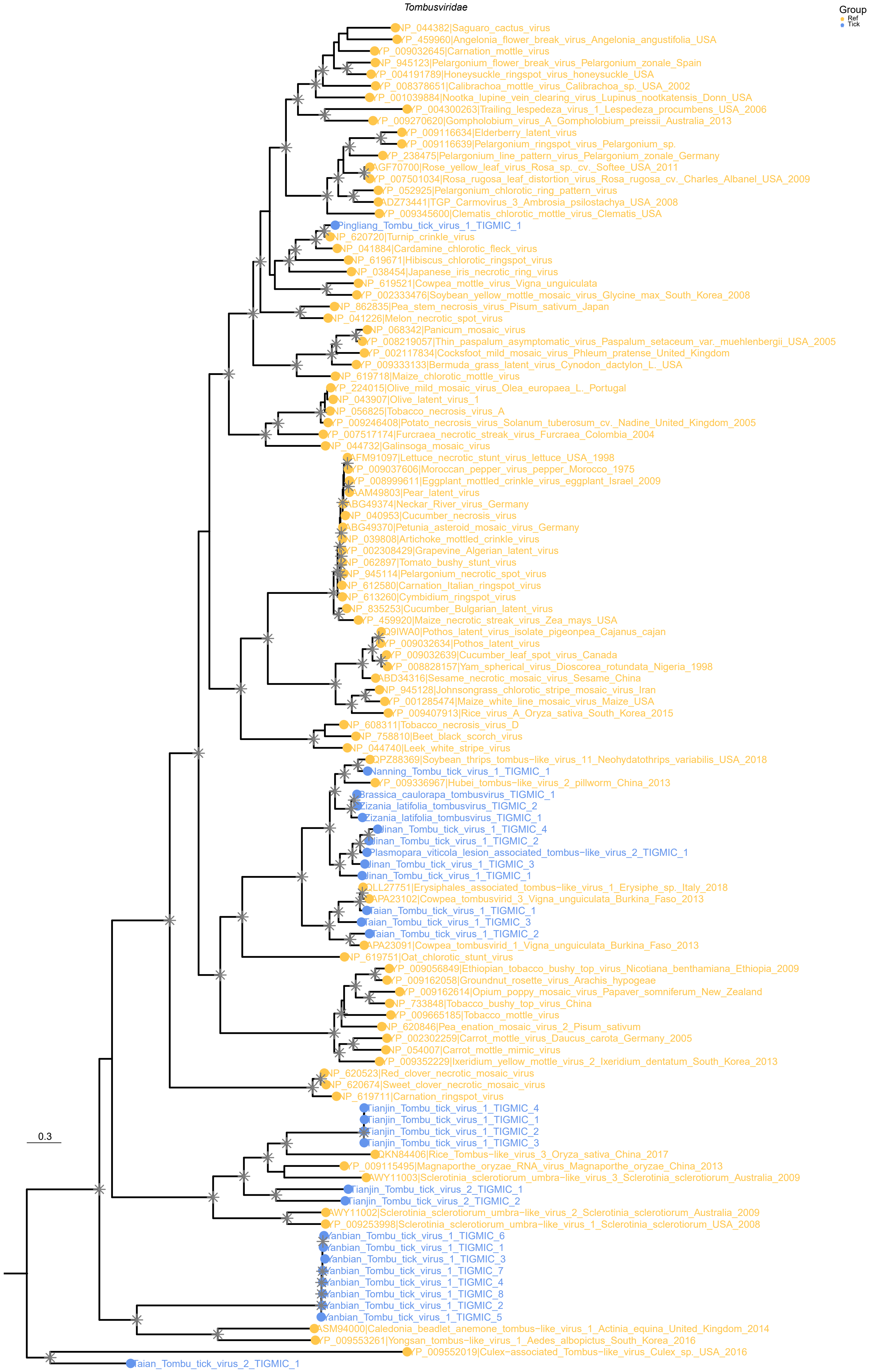
Tombusviridae
Group
Ref
Tick
0.3
NP_044382|Saguaro_cactus_virus
YP_459960|Angelonia_flower_break_virus_Angelonia_angustifolia_USA
YP_009032645|Carnation_mottle_virus
NP_945123|Pelargonium_flower_break_virus_Pelargonium_zonale_Spain
YP_004191789|Honeysuckle_ringspot_virus_honeysuckle_USA
YP_008378651|Calibrachoa_mottle_virus_Calibrachoa_sp._USA_2002
YP_001039884|Nootka_lupine_vein_clearing_virus_Lupinus_nootkatensis_Donn_USA
YP_004300263|Trailing_lespedeza_virus_1_Lespedeza_procumbens_USA_2006
YP_009270620|Gompholobium_virus_A_Gompholobium_preissii_Australia_2013
YP_009116634|Elderberry_latent_virus
YP_009116639|Pelargonium_ringspot_virus_Pelargonium_sp.
YP_238475|Pelargonium_line_pattern_virus_Pelargonium_zonale_Germany
AGF70700|Rose_yellow_leaf_virus_Rosa_sp._cv._Softee_USA_2011
YP_007501034|Rosa_rugosa_leaf_distortion_virus_Rosa_rugosa_cv._Charles_Albanel_USA_2009
YP_052925|Pelargonium_chlorotic_ring_pattern_virus
ADZ73441|TGP_Carmovirus_3_Ambrosia_psilostachya_USA_2008
YP_009345600|Clematis_chlorotic_mottle_virus_Clematis_USA
Pingliang_Tombu_tick_virus_1_TIGMIC_1
NP_620720|Turnip_crinkle_virus
NP_041884|Cardamine_chlorotic_fleck_virus
NP_619671|Hibiscus_chlorotic_ringspot_virus
NP_038454|Japanese_iris_necrotic_ring_virus
NP_619521|Cowpea_mottle_virus_Vigna_unguiculata
YP_002333476|Soybean_yellow_mottle_mosaic_virus_Glycine_max_South_Korea_2008
NP_862835|Pea_stem_necrosis_virus_Pisum_sativum_Japan
NP_041226|Melon_necrotic_spot_virus
NP_068342|Panicum_mosaic_virus
YP_008219057|Thin_paspalum_asymptomatic_virus_Paspalum_setaceum_var._muehlenbergii_USA_2005
YP_002117834|Cocksfoot_mild_mosaic_virus_Phleum_pratense_United_Kingdom
YP_009333133|Bermuda_grass_latent_virus_Cynodon_dactylon_L._USA
NP_619718|Maize_chlorotic_mottle_virus
YP_224015|Olive_mild_mosaic_virus_Olea_europaea_L._Portugal
NP_043907|Olive_latent_virus_1
NP_056825|Tobacco_necrosis_virus_A
YP_009246408|Potato_necrosis_virus_Solanum_tuberosum_cv._Nadine_United_Kingdom_2005
YP_007517174|Furcraea_necrotic_streak_virus_Furcraea_Colombia_2004
NP_044732|Galinsoga_mosaic_virus
AFM91097|Lettuce_necrotic_stunt_virus_lettuce_USA_1998
YP_009037606|Moroccan_pepper_virus_pepper_Morocco_1975
YP_008999611|Eggplant_mottled_crinkle_virus_eggplant_Israel_2009
AAM49803|Pear_latent_virus
ABG49374|Neckar_River_virus_Germany
NP_040953|Cucumber_necrosis_virus
ABG49370|Petunia_asteroid_mosaic_virus_Germany
NP_039808|Artichoke_mottled_crinkle_virus
YP_002308429|Grapevine_Algerian_latent_virus
NP_062897|Tomato_bushy_stunt_virus
NP_945114|Pelargonium_necrotic_spot_virus
NP_612580|Carnation_Italian_ringspot_virus
NP_613260|Cymbidium_ringspot_virus
NP_835253|Cucumber_Bulgarian_latent_virus
YP_459920|Maize_necrotic_streak_virus_Zea_mays_USA
Q9IWA0|Pothos_latent_virus_isolate_pigeonpea_Cajanus_cajan
YP_009032634|Pothos_latent_virus
YP_009032639|Cucumber_leaf_spot_virus_Canada
YP_008828157|Yam_spherical_virus_Dioscorea_rotundata_Nigeria_1998
ABD34316|Sesame_necrotic_mosaic_virus_Sesame_China
NP_945128|Johnsongrass_chlorotic_stripe_mosaic_virus_Iran
YP_001285474|Maize_white_line_mosaic_virus_Maize_USA
YP_009407913|Rice_virus_A_Oryza_sativa_South_Korea_2015
NP_608311|Tobacco_necrosis_virus_D
NP_758810|Beet_black_scorch_virus
NP_044740|Leek_white_stripe_virus
QPZ88369|Soybean_thrips_tombus-like_virus_11_Neohydatothrips_variabilis_USA_2018
Nanning_Tombu_tick_virus_1_TIGMIC_1
YP_009336967|Hubei_tombus-like_virus_2_pillworm_China_2013
Brassica_caulorapa_tombusvirus_TIGMIC_1
Zizania_latifolia_tombusvirus_TIGMIC_2
Zizania_latifolia_tombusvirus_TIGMIC_1
Jinan_Tombu_tick_virus_1_TIGMIC_4
Jinan_Tombu_tick_virus_1_TIGMIC_2
Plasmopara_viticola_lesion_associated_tombus-like_virus_2_TIGMIC_1
Jinan_Tombu_tick_virus_1_TIGMIC_3
Jinan_Tombu_tick_virus_1_TIGMIC_1
QLL27751|Erysiphales_associated_tombus-like_virus_1_Erysiphe_sp._Italy_2018
APA23102|Cowpea_tombusvirid_3_Vigna_unguiculata_Burkina_Faso_2013
Taian_Tombu_tick_virus_1_TIGMIC_1
Taian_Tombu_tick_virus_1_TIGMIC_3
Taian_Tombu_tick_virus_1_TIGMIC_2
APA23091|Cowpea_tombusvirid_1_Vigna_unguiculata_Burkina_Faso_2013
NP_619751|Oat_chlorotic_stunt_virus
YP_009056849|Ethiopian_tobacco_bushy_top_virus_Nicotiana_benthamiana_Ethiopia_2009
YP_009162058|Groundnut_rosette_virus_Arachis_hypogeae
YP_009162614|Opium_poppy_mosaic_virus_Papaver_somniferum_New_Zealand
NP_733848|Tobacco_bushy_top_virus_China
YP_009665185|Tobacco_mottle_virus
NP_620846|Pea_enation_mosaic_virus_2_Pisum_sativum
YP_002302259|Carrot_mottle_virus_Daucus_carota_Germany_2005
NP_054007|Carrot_mottle_mimic_virus
YP_009352229|Ixeridium_yellow_mottle_virus_2_Ixeridium_dentatum_South_Korea_2013
NP_620523|Red_clover_necrotic_mosaic_virus
NP_620674|Sweet_clover_necrotic_mosaic_virus
NP_619711|Carnation_ringspot_virus
Tianjin_Tombu_tick_virus_1_TIGMIC_4
Tianjin_Tombu_tick_virus_1_TIGMIC_1
Tianjin_Tombu_tick_virus_1_TIGMIC_2
Tianjin_Tombu_tick_virus_1_TIGMIC_3
QKN84406|Rice_Tombus-like_virus_3_Oryza_sativa_China_2017
YP_009115495|Magnaporthe_oryzae_RNA_virus_Magnaporthe_oryzae_China_2013
AWY11003|Sclerotinia_sclerotiorum_umbra-like_virus_3_Sclerotinia_sclerotiorum_Australia_2009
Tianjin_Tombu_tick_virus_2_TIGMIC_1
Tianjin_Tombu_tick_virus_2_TIGMIC_2
AWY11002|Sclerotinia_sclerotiorum_umbra-like_virus_2_Sclerotinia_sclerotiorum_Australia_2009
YP_009253998|Sclerotinia_sclerotiorum_umbra-like_virus_1_Sclerotinia_sclerotiorum_USA_2008
Yanbian_Tombu_tick_virus_1_TIGMIC_6
Yanbian_Tombu_tick_virus_1_TIGMIC_1
Yanbian_Tombu_tick_virus_1_TIGMIC_3
Yanbian_Tombu_tick_virus_1_TIGMIC_7
Yanbian_Tombu_tick_virus_1_TIGMIC_4
Yanbian_Tombu_tick_virus_1_TIGMIC_8
Yanbian_Tombu_tick_virus_1_TIGMIC_2
Yanbian_Tombu_tick_virus_1_TIGMIC_5
ASM94000|Caledonia_beadlet_anemone_tombus-like_virus_1_Actinia_equina_United_Kingdom_2014
YP_009553261|Yongsan_tombus-like_virus_1_Aedes_albopictus_South_Korea_2016
YP_009552019|Culex-associated_Tombus-like_virus_Culex_sp._USA_2016
Taian_Tombu_tick_virus_2_TIGMIC_1

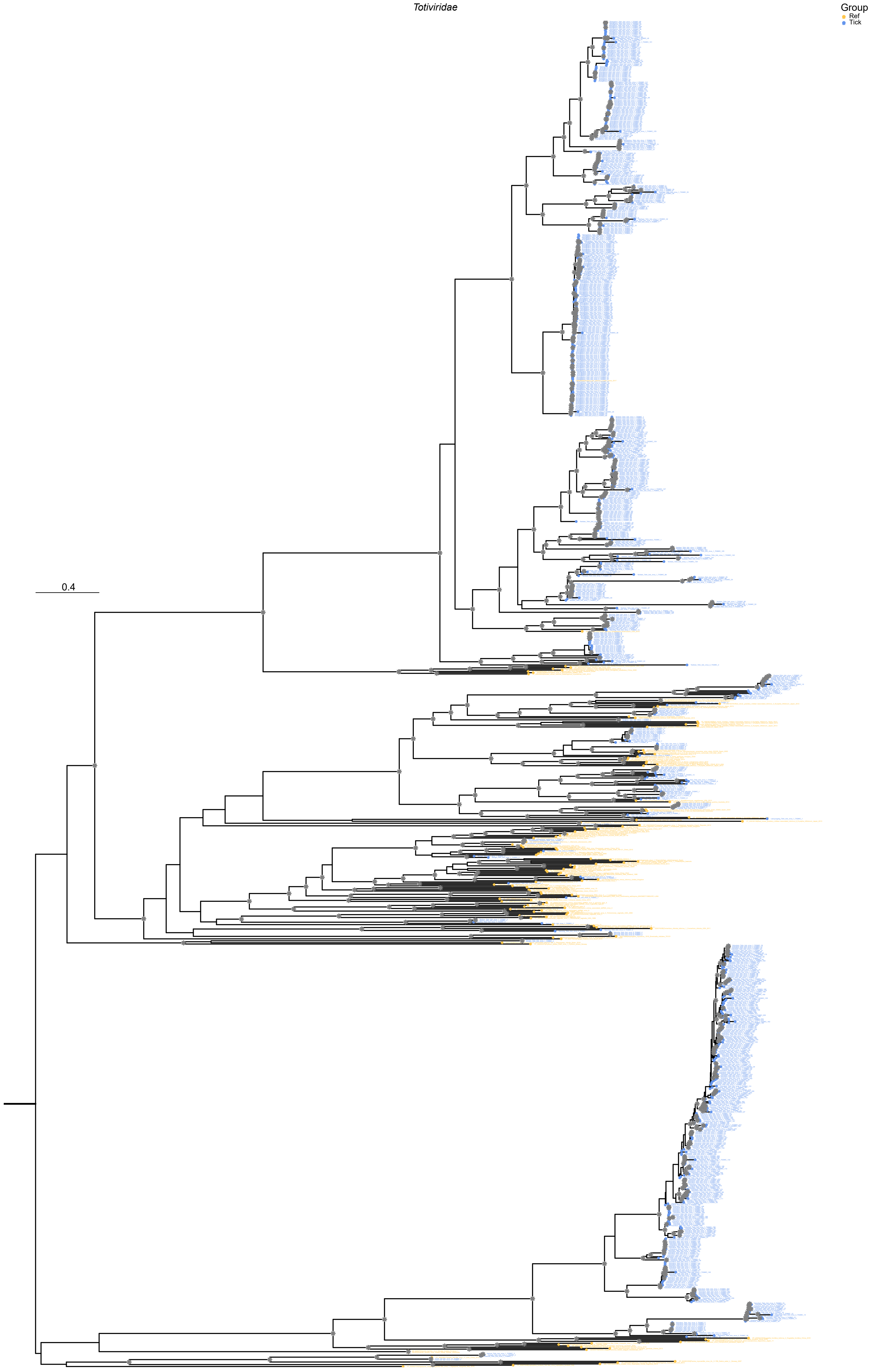
*Totiviridae*
Group
Ref
Tick
0.4
