# Supplementary material for: Metavirome of 31 tick species provides a compendium of 1,801 RNA virus genomes
Source: Nat Microbiol. 2023 Jan 5;8(1):162–73. doi: 10.1038/s41564-022-01275-w (PMC9816062; doi:10.1038/s41564-022-01275-w)
Supplement: Supplementary file 2 — Reporting Summary [file 41564_2022_1275_MOESM2_ESM.pdf]

nature portfolio

Corresponding author(s): Tommy Tsan-Yuk Lam, Na Jia and Wu-Chun Cao

Last updated by author(s): Mar 23, 2022

Reporting Summary

Nature Portfolio wishes to improve the reproducibility of the work that we publish. This form provides structure for consistency and transparency in reporting. For further information on Nature Portfolio policies, see our Editorial Policies and the Editorial Policy Checklist.

Statistics

For all statistical analyses, confirm that the following items are present in the figure legend, table legend, main text, or Methods section.

n/a	Confirmed	
☐	☒	The exact sample size (*n*) for each experimental group/condition, given as a discrete number and unit of measurement
☒	☐	A statement on whether measurements were taken from distinct samples or whether the same sample was measured repeatedly
☐	☒	The statistical test(s) used AND whether they are one- or two-sided<br>*Only common tests should be described solely by name; describe more complex techniques in the Methods section.*
☒	☐	A description of all covariates tested
☐	☒	A description of any assumptions or corrections, such as tests of normality and adjustment for multiple comparisons
☐	☒	A full description of the statistical parameters including central tendency (e.g. means) or other basic estimates (e.g. regression coefficient) AND variation (e.g. standard deviation) or associated estimates of uncertainty (e.g. confidence intervals)
☐	☒	For null hypothesis testing, the test statistic (e.g. *F*, *t*, *r*) with confidence intervals, effect sizes, degrees of freedom and *P* value noted<br>*Give P values as exact values whenever suitable.*
☒	☐	For Bayesian analysis, information on the choice of priors and Markov chain Monte Carlo settings
☒	☐	For hierarchical and complex designs, identification of the appropriate level for tests and full reporting of outcomes
☒	☐	Estimates of effect sizes (e.g. Cohen's *d*, Pearson's *r*), indicating how they were calculated

Our web collection on statistics for biologists contains articles on many of the points above.

Software and code

Policy information about availability of computer code

Data collection	No software was used for data collection.
Data analysis	Trimmomatic program v0.39, Trinity v2.8.5 program,BLAST tool, DIAMOND version 0.9.24, Lasergene package v7.1, Bowtie2 v2.4.3, TransDecoder v5.5.0, CD-HIT v4.8.1, Python package "skbio" v0.5.6, SPSS v 20.0, "tsne" v 0.1-3, GATK 4.0 pipeline, IQ-Tree v.1.6.1, HTSeq v-0.6.0, R package "edgeR" v3.34.1 , topGO package v2.3.4, MAFFT v7.490, TrimAl v1.2, ggtree package version 3.0.4, Python package ETE toolkits- v3.1.2, TreeCluster v1.0.3, MrBayes v.3.2, BaTS program v1.0, ArcGIS 10.2

For manuscripts utilizing custom algorithms or software that are central to the research but not yet described in published literature, software must be made available to editors and reviewers. We strongly encourage code deposition in a community repository (e.g. GitHub). See the Nature Portfolio guidelines for submitting code & software for further information.

Data

Policy information about availability of data

All manuscripts must include a data availability statement. This statement should provide the following information, where applicable:

- Accession codes, unique identifiers, or web links for publicly available datasets
- A description of any restrictions on data availability
- For clinical datasets or third party data, please ensure that the statement adheres to our policy

The sequencing data have been deposited to SRA under Bioproject PRJNA841744, and the assembled virus sequences have been submitted to GenBank (accession no. ON746331-ON746566, ON811696-ON813070, ON811604-ON811608, ON872591-ON872654), and BIGD (https://ngdc.cncb.ac.cn/gsub/) under the Project (PRJCA008467) (accession no. GWHBHNN00000000, GWHBHNO00000000, GWHBHNP00000000, GWHBHNQ00000000, GWHBHNR00000000, GWHBHNS00000000, GWHBHNT00000000, GWHBHNU00000000).

Field-specific reporting

Please select the one below that is the best fit for your research. If you are not sure, read the appropriate sections before making your selection.

☐ Life sciences ☐ Behavioural & social sciences ☒ Ecological, evolutionary & environmental sciences

For a reference copy of the document with all sections, see nature.com/documents/nr-reporting-summary-flat.pdf

Ecological, evolutionary & environmental sciences study design

All studies must disclose on these points even when the disclosure is negative.

Study description	This study is a tick meta-transcriptomics research on 31 tick species of 2 families from 148 sites throughout 30 provinces of mainland China using RNA sequencing approach, to comprehensively profile the tick virome, assess factors shaping the virome compostion, and identify the evolutionary role of ticks on virus diversification.
Research sample	Around 8100 adult ticks of 31 tick species of 2 families from 148 sites throughout 30 provinces of mainland were prepared.
Sampling strategy	More than 20 ticks of the same tick species from the same sampling location were collected to ensure the sufficient samples for the downstream statistical analysis.
Data collection	10-30 ticks were polled for RNA sequencing, and the total of 607 libraries were prepared. The sequencing depth waa around 10 G for each library .
Timing and spatial scale	We collected samples from March 2016 to October 2019, the frequency is around 10 times for each month during the peak period of ticks (from March to October).
Data exclusions	No data were excluded from the analyses.
Reproducibility	PCR assays were performed to confirm the important sequencing findings.
Randomization	All samples were randomly allocated into different groups according their biological characteristics, such as tick species, gender, bloodmeal status, ect.
Blinding	All data were analyzed blindingly without grouping factors informed, only statistical comparison were taken according to grouping information.

Did the study involve field work? ☒ Yes ☐ No

Field work, collection and transport

Field conditions	The 148 sampling sites throughout 30 provinces of mainland China covered seven ecological types including coniferous forest, steppe, farmland, desert, shrubland, and tropical forest.
Location	Latitude and longitude were recorded for each sampling sites.
Access & import/export	We only enter the permitted habitats for sample collection.
Disturbance	No disturbance were caused.

Reporting for specific materials, systems and methods

We require information from authors about some types of materials, experimental systems and methods used in many studies. Here, indicate whether each material, system or method listed is relevant to your study. If you are not sure if a list item applies to your research, read the appropriate section before selecting a response.

Materials & experimental systems

n/a	Involved in the study	
☒	☐	Antibodies
☒	☐	Eukaryotic cell lines
☒	☐	Palaeontology and archaeology
☐	☒	Animals and other organisms
☒	☐	Human research participants
☒	☐	Clinical data
☒	☐	Dual use research of concern

Methods

n/a	Involved in the study	
☒	☐	ChIP-seq
☒	☐	Flow cytometry
☒	☐	MRI-based neuroimaging

Animals and other organisms

Policy information about studies involving animals; ARRIVE guidelines recommended for reporting animal research

Laboratory animals	*For laboratory animals, report species, strain, sex and age OR state that the study did not involve laboratory animals.*
Wild animals	*Provide details on animals observed in or captured in the field; report species, sex and age where possible. Describe how animals were caught and transported and what happened to captive animals after the study (if killed, explain why and describe method; if released, say where and when) OR state that the study did not involve wild animals.*
Field-collected samples	A total of 8,182 ticks of 31 species were collected from 148 sampling sites throughout 30 provinces of mainland China.
Ethics oversight	*Identify the organization(s) that approved or provided guidance on the study protocol, OR state that no ethical approval or guidance was required and explain why not.*

Note that full information on the approval of the study protocol must also be provided in the manuscript.
